# Synthesis, Crystal Structures and Spectroscopic Properties of Triazine-Based Hydrazone Derivatives; A Comparative Experimental-Theoretical Study

**DOI:** 10.3390/molecules20045851

**Published:** 2015-04-03

**Authors:** Muhammad Nadeem Arshad, Aisha Bibi, Tariq Mahmood, Abdullah M. Asiri, Khurshid Ayub

**Affiliations:** 1 Chemistry Department, Faculty of Science, King Abdulaziz University, P.O. Box 80203, Jeddah 21589, Saudi Arabia; E-Mail: aasiri2@gmail.com; 2 Department of Chemistry, COMSATS Institute of Information Technology, University Road, Tobe Camp, 22060 Abbottabad, Pakistan; E-Mails: aishabibi657@gmail.com (A.B.); khurshid@ciit.net.pk (K.A.); 3 Center of Excellence for Advanced Materials Research (CEAMR), King Abdulaziz University, P.O. Box 80203, Jeddah 21589, Saudi Arabia; 4 Department of Chemistry, College of Science, King Faisal University, Al-Hafouf 31982, Saudi Arabia

**Keywords:** triazine, hydrazone, X-ray, DFT, MEP, first hyperpolarizability

## Abstract

We report here a comparative theoretical and experimental study of four triazine-based hydrazone derivatives. The hydrazones are synthesized by a three step process from commercially available benzil and thiosemicarbazide. The structures of all compounds were determined by using the UV-Vis., FT-IR, NMR (^1^H and ^13^C) spectroscopic techniques and finally confirmed unequivocally by single crystal X-ray diffraction analysis. Experimental geometric parameters and spectroscopic properties of the triazine based hydrazones are compared with those obtained from density functional theory (DFT) studies. The model developed here comprises of geometry optimization at B3LYP/6-31G (d, p) level of DFT. Optimized geometric parameters of all four compounds showed excellent correlations with the results obtained from X-ray diffraction studies. The vibrational spectra show nice correlations with the experimental IR spectra. Moreover, the simulated absorption spectra also agree well with experimental results (within 10–20 nm). The molecular electrostatic potential (MEP) mapped over the entire stabilized geometries of the compounds indicated their chemical reactivates. Furthermore, frontier molecular orbital (electronic properties) and first hyperpolarizability (nonlinear optical response) were also computed at the B3LYP/6-31G (d, p) level of theory.

## 1. Introduction

**Figure 1:**
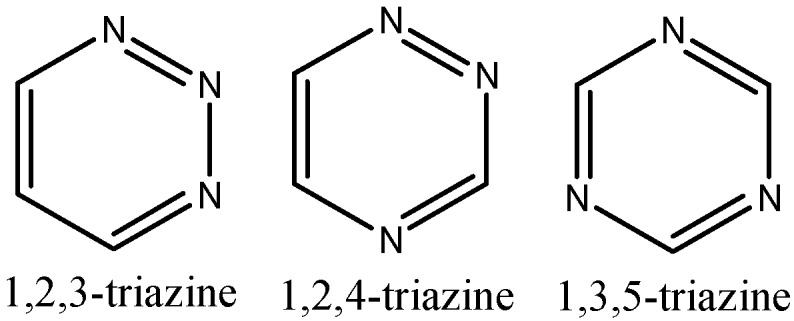
Basic skeleton of different types of triazines.

Heterocyclic chemistry is essential to biology and medicine. It is not questionable to say that we are living in the age of heterocyclic chemistry. Triazines are heterocyclic analogue of benzene containing three nitrogen atoms with moelcular formula C_3_H_3_N_3_. Three isomeric triazines are shown in Figure 1.

In 1776, Scheele synthesized triazine through pyrolysis of uric acid, and later Serullas repeated the work of Scheele however, the structure was first confirmed by Leibig and Wohler [1]. Molecules containing triazine skeletons show considerable biological and pharmaceutical activities [2,3]. 2-Ethoxymethyl-6-ethyl-2,3,4,5-tetrahydro-1,2,4-triazine-3,5-dione was synthesized and evaluated for its antimicrobial activity [4] and later its molecular and vibrational spectra were investigated by applying density functional theory methods [5]. Hydrazides of 1,2,4-triazine have been used as precursors for the synthesis of various biologically active compounds [3]. Moreover, these molecules are also efficient in dye sensitized solar cells (DSSC) [6].

Hydrazones are another class of compounds well known to possess various kinds of biological activities [7]. They have been used as an intermediate for the preparation of heterocycles displaying useful activities [8,9]. Recently Jani *et al.* has reported the synthesis and antimicrobial activities of complexes prepared using ligands containing triazine and hydrazone functionalities [10]. The vast range of pharmacological applications of these classes of compounds encouraged us to synthesize compounds containing both triazine and hydrazone moieties. We report here the synthesis, crystal structures and spectroscopic properties of some 1,2,4-triazine-based hydrazone derivatives. In continuation to our on-going studies of various class of heterocyclic molecules [11,12,13], density functional theory (DFT) studies were also performed not only to validate the experimental results, but also to explore further structural properties as well.

## 2. Results and Discussion

### 2.1. Synthesis

**Scheme 1:**
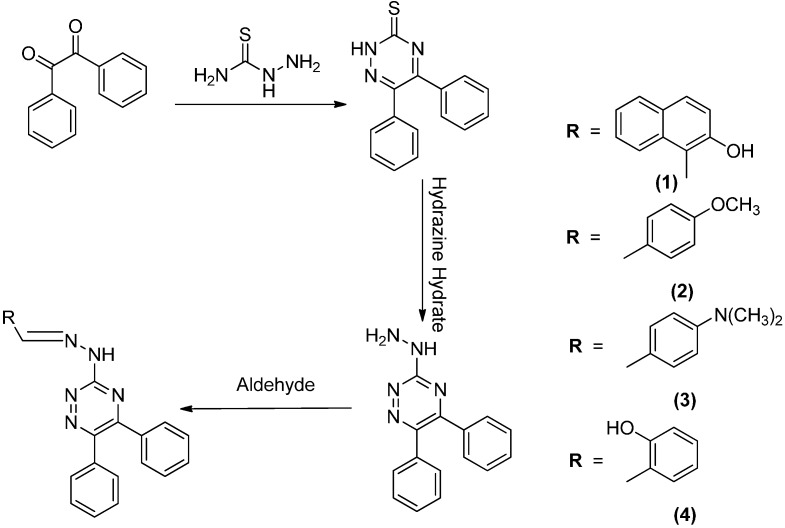
Synthetic scheme of target compounds **1**–**4**.

The synthesis of all four triazine based hydrazone derivatives **1**–**4** was performed in three steps starting from commercially available benzil. The first step was condensation of benzil with thiosemicarbazide followed by another condensation of the intermediate with hydrazine hydrate to construct the triazine ring. The hydrazine intermediate was then reacted with the respective aldehydes to deliver hydrazones **1**–**4** as the final products (Scheme 1).

### 2.2. Crystallographic Studies

**Figure 2:**
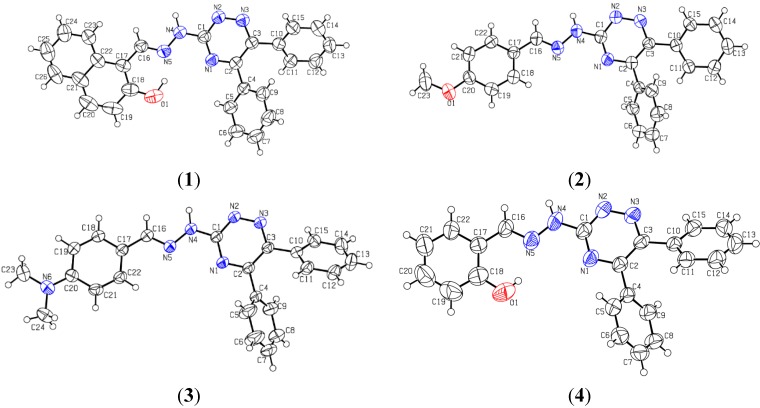
ORTEP diagrams of compounds **1**–**4**. Thermal ellipsoids were drawn at 50% probability level.

All four compounds were crystalized from dimethyl sulfoxide (DMSO) solution while standing in the lab at room temperature for about three months. In order to establish the X-ray structures suitable crystals were picked under a microscope. The structures of the compounds were established in order to understand their three dimensional interactions in a unit cell. Complete crystallographic parameters of all compounds **1**–**4** are provided in Table 1. The ORTEP diagrams of all compounds are shown in Figure 2.

**Table 1:** Crystal data and structure refinement parameters.

Identification Code	1	2	3	4
Empirical formula	C_26_H_19_N_5_O	C_23_H_19_N_5_O	C_24_H_22_N_6_	C_22_H_17_N_5_O
Formula weight	417.46	381.43	394.48	367.41
Temperature/K	296.15	296.15	296.15	296.15
Crystal system	monoclinic	monoclinic	triclinic	monoclinic
Space group	P2_1_/n	P2_1_/c	P-1	P2_1_/c
a/Å	6.5745(2)	5.9756(18)	8.8933(10)	14.9753(17)
b/Å	19.9133(6)	21.573(6)	11.1477(11)	6.1220(6)
c/Å	15.9805(5)	14.980(4)	11.7484(13)	21.038(2)
α/°	90.00	90.00	91.212(9)	90.00
β/°	96.369(3)	93.45(2)	106.044(10)	103.992(11)
γ/°	90.00	90.00	108.519(9)	90.00
Volume/Å^3^	2079.25(11)	1927.7(9)	1053.8(2)	1871.6(3)
Z	4	4	2	4
ρ_calc_mg/mm^3^	1.334	1.314	1.243	1.304
m/mm^−1^	0.677	0.084	0.077	0.084
F(000)	872.0	800.0	416.0	768.0
Crystal size/mm^3^	0.48 × 0.16 × 0.15	0.48 × 0.08 × 0.05	0.32 × 0.26 × 0.17	0.43 × 0.15 × 0.05
2θ range for data collection	7.12 to 152°	5.76 to 58.66°	5.62 to 58.9°	5.6 to 59.14°
Index ranges	−6 ≤ h ≤ 8,	−7 ≤ h ≤ 8,	−11 ≤ h ≤ 9,	−19 ≤ h ≤ 19,
	−25 ≤ k ≤ 21,	−27 ≤ k ≤ 24,	−12 ≤ k ≤ 14,	−8 ≤ k ≤ 7,
	−20 ≤ l ≤ 19	−20 ≤ l ≤ 18	−16 ≤ l ≤ 15	−29 ≤ l ≤ 26
Reflections collected	12339	11554	9379	11939
Independent reflections	4304[R(int) = 0.0185]	4608[R(int) = 0.0727]	5004[R(int) = 0.0252]	4597[R(int) = 0.0620]
Data/restraints/parameters	4304/0/290	4608/1/266	5004/0/274	4597/1/257
Goodness-of-fit on F^2^	1.036	1.012	1.038	0.961
Final R indexes [I ≥ 2σ (I)]	R_1_ = 0.0428,	R_1_ = 0.0626,	R_1_ = 0.0541,	R_1_ = 0.0601,
	wR_2_ = 0.1156	wR_2_ = 0.0925	wR_2_ = 0.1221	wR_2_ = 0.1236
Final R indexes [all data]	R_1_ = 0.0546,	R_1_ = 0.1988,	R_1_ = 0.0840,	R_1_ = 0.1880,
	wR_2_ = 0.1273	wR_2_ = 0.1358	wR_2_ = 0.1423	wR_2_ = 0.1670
Largest diff. peak/hole/e Å^−3^	0.19/−0.20	0.16/−0.19	0.25/−0.16	0.13/−0.16

**Figure 3:**
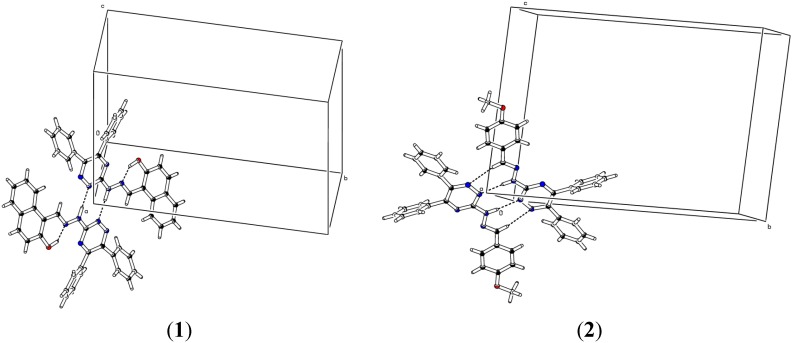

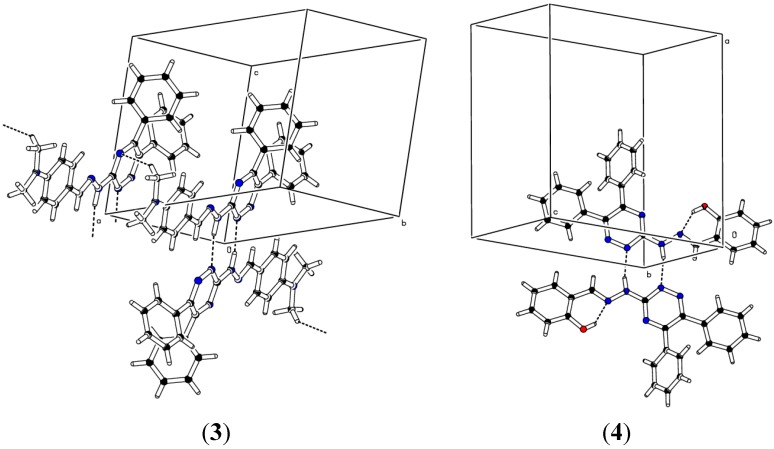
Unit cell diagrams of compounds **1**–**4** showing the inter- and intramolecular hydrogen bonding.

Compounds **1**–**4** are structurally similar regarding the triazine moiety and the two phenyl rings attached to it. However, these hydrazones differ in the hydrazomethyl moieties attached the central triazine ring. The triazine ring is planar mainly because all atoms are sp^2^ hybridized. Root mean square deviation values of the fitted atoms of ring are 0.0285(2) Å, 0.0215(1) Å, 0.0187(1) Å and 0.0264 Å for molecules **1**–**4**, respectively. The dihedral angles between the triazine ring and substituted phenyl rings are 37.99(5)° and 43.08(6)° in **1**, 60.39(7)° and 31.77(1)° in **2**, 61.08(8)° and 44.91(6)° in **3** and 50.02(7)° and 34.70(1)° in compound **4**. The two aromatic rings attached to the triazine moiety are twisted at dihedral angles of 55.01(5)°, 61.85(7)°, 56.46(9)° and 57.22(7)° in all compounds **1**–**4** respectively. The different dihedral angles reflect that all molecules have different spatial environment as well as Vander Walls’ interactions in their unit cells (Figure 3). Hydrogen bond parameters of all compounds have been listed in Table 2.

Compound **1** and **4** formed inverted dimers through only one carboxylic type N-H…N interaction. The compound **2** formed inverted dimers via N-H…N classical type interactions (two). Compound **3** involved the formation of dimers through classical N-H…N interaction. These dimers are further connected through non-classical C-H…N interaction in zig-zag manner (Figure 3). Triazine moiety is not planer with the substituted phenyl rings (C17-C22) and dihedral angles of compounds **1**–**4** are 14.99(7)°, 5.13(2)°, 3.27(1)° and 14.04(2)°, respectively. Intramolecular hydrogen bonding has been observed in compound **1** and **4** bearing hydroxyl groups at the *ortho* positions to produce an *S*(6) six membered ring motif.

**Table 2:** Hydrogen bond parameters of compounds **1**–**4**.

D	H	A	d(D-H)/Å	d(H-A)/Å	d(D-A)/Å	D-H-A/°
**(1)**						
N4	H2	N2^1^	0.97	1.91	2.8750(18)	171.9
**(2)**						
C16	H16	N3^2^	0.93	2.58	3.496(4)	168.5
N4	H1	N2^2^	0.893(10)	2.047(11)	2.939(3)	176(3)
**(3)**						
N4	H1	N2^3^	0.97	2.03	2.995(2)	168.8
C24	H24C	N1^4^	0.96	2.59	3.449(3)	148.7
**(4)**						
O1	H1O	N5	0.82	1.93	2.646(3)	146.1
N4	H2	N2^5^	0.889(10)	2.033(12)	2.907(4)	167(3)
^1^2 − X, −Y, −Z, ^2^1 − X, 1 − Y, 1 − Z, ^3^−X, −Y, −Z, ^4^1 + X, +Y, +Z, ^5^−X, 2 − Y, −Z						

### 2.3. Geometries Optimization

**Figure 4:**
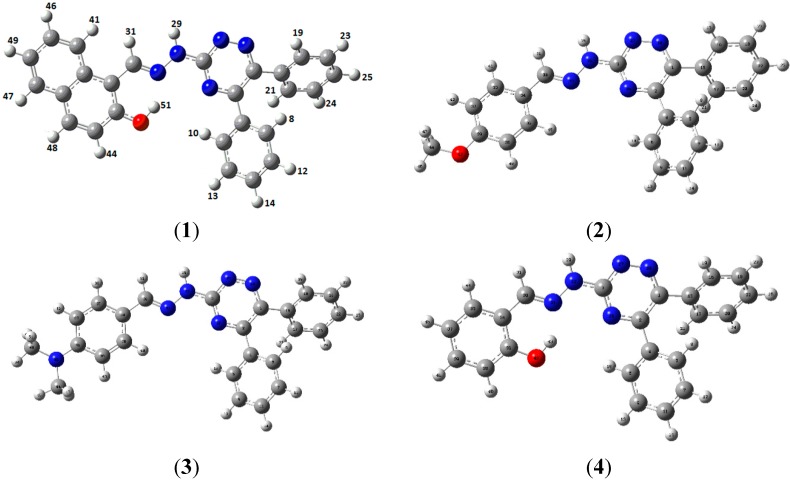
Optimized geometries of compounds (**1**–**4**) at B3LYP/6-31G (d, p) level of DFT.

Theoretical studies have been performed to compare the geometric parameters with those obtained from X-ray diffraction studies. The geometric parameters of title compounds **1**–**4** were optimized using Gaussian 09 program at B3LYP/6-31G (d, p) level of DFT. Optimized geometries are shown in Figure 4 and comparison of simulated bond lengths and bond angles of compounds **1**–**4** along with X-ray values are listed in Table 3, Table 4, Table 5 and Table 6, respectively.

**Table 3:** Selected molecular structure parameters of compound **1**.

**Bond Lengths (A°) Experimental B3LYP/6-31G (d, p)**		
O1-C18	1.353(2)	1.430
N1-C1	1.341(18)	1.335
N1-C2	1.327(18)	1.335
N2-N3	1.328(17)	1.315
N2-C1	1.338(19)	1.354
N3-C3	1.336(17)	1.347
N4-N5	1.364(18)	1.354
N4-C1	1.356(19)	1.368
N5-C16	1.286(19)	1.294
**Bond Angles (°)**		
C2-N1-C1	116.2(12)	116.3
N3-N2-C1	118.1(12)	116.9
N2-N3-C3	120.2(12)	121.2
C1-N4-N5	119.4(12)	123.1
C16-N5-N4	117.9(13)	116.6
N1-C1-N4	119.3(13)	120.6
N2-C1-N1	125.4(13)	125.7
N2-C1-N4	115.2(13)	113.5
N1-C2-C3	120.0(12)	119.4
N1-C2-C4	114.6(12)	115.1
N3-C3-C2	119.4(13)	118.9
N3-C3-C10	115.0(12)	114.7
O1-C18-C19	116.1(16)	118.9

**Table 4:** Selected molecular structure parameters of compound **2**.

**Bond Lengths (A°) Experimental B3LYP/6-31G (d, p)**		
O1-C20	1.377(3)	1.362
O1-C23	1.426(3)	1.419
N1-C1	1.332(3)	1.334
N1-C2	1.330(3)	1.335
N2-N3	1.343(3)	1.316
N2-C1	1.353(3)	1.353
N3-C3	1.334(3)	1.346
N4-N5	1.387(3)	1.351
N4-C1	1.372(3)	1.370
**Bond Angles (°)**		
C2-N1-C1	115.6(2)	116.3
N3-N2-C1	117.3(2)	116.7
N2-N3-C3	120.2(2)	121.2
C1-N4-N5	121.9(2)	121.9
C16-N5-N4	112.5(2)	119.1
N1-C1-N4	122.1(2)	119.7
N2-C1-N1	126.3(2)	126.1
N2-C1-N4	111.6(3)	114.0
N1-C2-C3	120.9(3)	119.1
N1-C2-C4	115.5(2)	115.3
N3-C3-C2	119.4(2)	119.1
N3-C3-C10	113.9(2)	114.7
C20-O1-C23	117.6(3)	118.5

**Table 5:** Selected molecular structure parameters of compound **3**.

**Bond Lengths (A°) Experimental B3LYP/6-31G (d, p)**		
O1-C1	1.347(2)	1.521
N1-C2	1.325(2)	1.402
N2-N3	1.333(2)	1.540
N2-C1	1.351(2)	1.395
N3-C3	1.336(2)	1.402
N4-N5	1.381(2)	1.540
N4-C1	1.349(2)	1.540
N5-C16	1.282(2)	1.540
N6-C20	1.372(2)	1.383
**Bond Angles (°)**		
C2-N1-C1	115.3(14)	116.3
N3-N2-C1	118.3(13)	119.9
N2-N3-C3	119.6(14)	119.6
C1-N4-N5	122.4(14)	109.4
C16-N5-N4	113.8(14)	109.4
N1-C1-N4	120.7(15)	120.9
N2-C1-N1	125.4(15)	118.1
N2-C1-N4	113.7(14)	120.9
N1-C2-C3	121.2(15)	120.4
N1-C2-C4	116.5(15)	119.1
N3-C3-C2	119.7(14)	120.4
N3-C3-C10	116.5(14)	119.1

**Table 6:** Selected molecular structure parameters of compound **4**.

**Bond Lengths (A°) Experimental B3LYP/6-31G (d)**		
O1-C18	1.357(4)	1.341
N1-C1	1.336(3)	1.334
N1-C2	1.337(3)	1.336
N2-N3	1.338(3)	1.317
N2-C1	1.338(3)	1.350
N3-C3	1.339(3)	1.346
N4-N5	1.383(3)	1.351
N4-C1	1.360(3)	1.371
N5-C16	1.271(3)	1.291
**Bond Angles (°)**		
C2-N1-C1	115.9(2)	116.3
N3-N2-C1	117.9(2)	116.7
N2-N3-C3	120.4(2)	121.2
C1-N4-N5	122.5(3)	121.8
C16-N5-N4	115.1(2)	119.2
N1-C1-N4	120.9(3)	119.6
N2-C1-N1	126.0(3)	126.1
N2-C1-N4	113.1(3)	114.0
N1-C2-C3	120.4(2)	119.0
N1-C2-C4	116.0(2)	115.3
N3-C3-C2	119.1(2)	119.1
N3-C3-C10	114.3(2)	114.6
O1-C18-C19	118.3(3)	115.8

The results listed in Table 3, Table 4, Table 5 and Table 6 show that X-ray and simulated bond lengths/angles of all atoms in compounds **1**–**4** correlated nicely. Deviation in the selected bond lengths/angles of compound **1** was observed in the range of 0.006–0.077 Å/0.1–3.7°. Maximum deviation in bond length was 0.077 Å for O1-C18. Similarly maximum deviation in bond angle was 3.7° for C1-N4-N5. For **2** deviation in selected bond lengths/angles was found in the range 0–0.036 Å/0–6.6°. Similarly the maximum deviation in bond lengths/angles of compound **3** and **4** was observed in the range 0.011–0.258 Å/0–7.3° and 0.001–0.032 Å/0–4.1°.

Comparative analyses of the geometric data of compounds **1**–**4** also indicate that the intramolecular hydrogen bonding affect the geometric parameters, particularly the C1-N2, of compound **1** and **4**. The C1-N2 bond length decreases with the increase in the strength of intramolecular hydrogen bonding. The intramolecular hydrogen bonding is stronger in compound **1** compared to compound **4**, and it is reflected in shortened O1-C18 bond length (1.353) of compound **1**. Moreover, N4-N5 bond length is also shortened for compound **1** (strong intramolecular hydrogen bonding). The bond lengths in the triazine central ring depend not only on the strength of the intramolecular hydrogen bonding but also on the strength of mesomeric electron donation. In general, relatively shorter bond lengths are observed for compound **1** whereas compound **2** shows relatively larger bond lengths. Relatively shorter bond lengths for compounds **3** compared to **2** can be attributed to relatively strong conjugation of NMe_2_ group (strong donor than methoxy through resonance) with the triazine skeleton.

### 2.4. Vibrational Analysis

**Figure 5:**
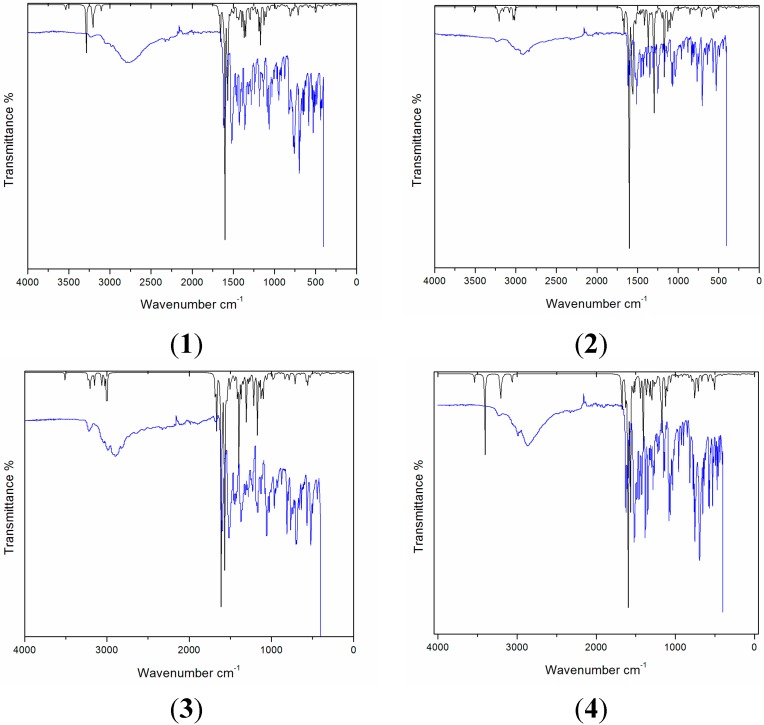
Simulated (balck) and experimental (blue) vibrational spectra of compounds **1**–**4**.

Vibrational spectroscopy is a spectroscopic technique used in chemistry for the identification of functional groups in order to elucidate the structure of a target molecule, kinetics of chemical reactions, *etc.* Both experimental and simulated spectra are shown in the Figure 5. Comparison of prominent frequencies of compounds **1**–**4** has given in Table 7.

For the correction of theoretical errors in this work, the theoretical harmonic frequencies above 1700 cm^−1^ were scaled by a scaling factor of 0.958, and frequencies less than 1700 cm^−1^ were scaled by 0.9627 [14]. Compounds **1**–**4** mainly have NH, N=CH and aromatic ring functional groups. These compounds have very comparable structures regarding functional groups except compound **3** which bears a dimethyl amino group. Therefore, the vibrations in compounds **1**–**4** are very comparable. For example, C=C aromatic vibrations are observed at 1611 (**1**), 1611 (**2**), 1593 (**3**) and 1621 cm^−1^ (**4**). The lower stretching vibration for compound **3** compared to **1**, **2** and **4** may be attributed to the better conjugation of the dimethylamino group with the triazine skeleton. Despite similar vibrations, characteristic distinguishing vibrations could be seen for each compound. For example, methyl groups in **3** appear at 2950 (asymmetric stretching), 2947 (asymmetric stretching), 2891 (symmetric stretching), 2884 cm^−1^ (symmetric stretching). Moreover, the C-N stretching vibration is observed at 1341 cm^−1^. Compound **2** was characterized by an O-Ph stretching vibration at 1244 cm^−1^.

**Table 7:** Prominent experimental and simulated vibrational (cm^−1^) values of **1**–**4**.

1			2			3			4		
Exp. B3LYP Assignment			Exp. B3LYP Assignment			Exp. B3LYP Assignment			Exp. B3LYP Assignment		
1611 1517	1595	ѴC=C_arom_	1611 1513	1605	ѴC=C	1593 1507	1605	ѴC=C	1621 1515	1613	ѴC=C_arom_
	1542	ѴC=N_arom_		1542	ѴC=N_arom_		1550	ѴC=N		1565	ѴC=C_arom_
	1512	ѴC=N_arom_		1511	ѴC=N_arom_		1538	ѴC=N_arom_		1536	ѴC=N_arom_
1367 1280	1506	ѴN=C_arom_	1350 1247	1499	ѴC=C_arom_	1428 1363	1511	ѴN=C_arom_	1375 1279	1510	ѴC=N_arom_
	1319	ѴC-C_arom_		1318	ѴC-C_arom_		1508	ѴC=N_arom_		1353	βCH_arom_
	1301	ѴC-C_arom_		1250	ѴO-Ph		1341	ѴNPh		1251	ѴC-C_arom_
1239	1252	ѴC-C_arom_	1167	1127	βCH_arom_	1277	1257	ѴC-C_arom_	1133	1127	ѴC=N_arom_
1179	1127	ѴC=N_arom_		1126	ѴC=N_arom_		1171	βCH_arom_	1078	1081	βCH_arom_
1062	1061	βCH_arom_	1060	1080	βCH_arom_	1160	1126	ѴC=N_arom_	1062	1061	βCH_arom_
756	775	βCH_arom_		1060	βCH_arom_	1052	1079	βCH_arom_	750	724	γCH_arom_
			764	759	γCH_arom_	807	803	γCH_arom_	688	686	γCH_arom_
						516	540	γCH_arom_			

From Table 7 it is clear that an excellent agreement exists among simulated and experimental vibrations. In compound **1**, a strong simulated C=C_arom._ stretching vibration appeared at 1595 cm^−1^ and showed nice agreement with the experimental 1611 cm^−1^. Aromatic C=C stretching vibrations in **2** depicted at 1605 cm^−1^ theoretically, and correlates with the experimental 1611 cm^−1^. Similarly the computed aromatic C=C vibrations in compound **3** and **4** showed very nice agreement between theory and experiment. Prominent C=N stretching vibrations of **1** found at 1542 cm^−1^, 1506 cm^−1^ and 1127 cm^−1^ in simulated spectrum also shows strong agreement with the experimental counterparts appearing at 1517 cm^−1^, 1179 cm^−1^. The C=N stretching vibrations of **2**, **3** and **4** also correlated excellently between theory and experiment (for individual values see Table 7).

### 2.5. Nuclear Magnetic Resonance Studies (^1^H-NMR)

The versatility in nuclear magnetic resonance spectroscopy makes it an unavoidable tool for the structural identification of compounds. Experimentally, NMR (both ^1^H and ^13^C) of all compounds **1**–**4** were recorded in DMSO and spectra are shown in the (Figure S1, Figure S2, Figure S3, Figure S4, Figure S5, Figure S6, Figure S7 and Figure S8, Supplementary Information). ^1^H-NMR chemical shift calculations were performed by using the fully optimized geometries at B3LYP/6-311+G (2d, p) level by adopting the GIAO method using the internal reference standard *i.e.*, tetramethylsilane. The detailed simulated ^1^H-NMR chemical shift values are listed in Table 8.

All compounds have NH protons that appeared at 8.43 ppm (**1**), 8.49 ppm (**2**), 8.42 ppm (**3**) and 8.34 ppm (**4**) theoretically, whereas the experimental peaks appeared at 12.31 ppm (**1**), 11.80 ppm (**2**), 11.62 ppm (**3**), and 12.26 ppm (**4**). NH protons are solvent and environment dependent; therefore, it is very hard to compare the theoretical values with the experimental ones [15]. Compounds **1** and **4** contain OH functional groups and are involved in intramolecular hydrogen bonding as depicted from X-ray studies, therefore, these protons appeared theoretically at 12.87 ppm (**1**), 11.34 ppm (**4**), and show excellent correlation with the experimental values at 12.77 ppm (**1**), 11.47 ppm (**4**). Computed chemical shift values of azomethine protons appeared at 9.39 ppm (**1**), 8.00 ppm (**2**), 7.93 ppm (**3**) and 8.28 ppm (**4**), whereas experimental values appeared at 9.33 ppm (**1**), 8.22 ppm (**2**), 9.65 ppm (**3**) and 8.45 ppm (**4**) respectively. Aromatic protons of all compounds showed very excellent correlation between experimental and theoretically calculated chemical shifts. Simulated aromatic protons of **1** appeared in the range 8.98–7.28 ppm, which agrees very well with the experimental chemical shifts at 8.23–7.25 ppm. For compound 2, theoretical chemical shifts of aromatic protons (8.79–6.83 ppm) also correlate very nicely with the experimental chemical shifts (7.61–7.00 ppm). Similarly theoretical aromatic protons of **3** and **4** appeared in the range 8.87–6.74 ppm and 8.87–6.99 ppm, respectively which correlate nicely with the experimental chemical shifts at 7.68–6.74 ppm and 7.67–7.00 ppm. Theoretical data of all compounds showed close agreement with the experimental data.

**Table 8:** Simulated ^1^H-NMR chemical shifts of **1**–**4** (ppm) at B3LYP/6-311+G (2d, p) level (atomic labels are with reference to Figure 4).

(1)	B3LYP (ppm)	(2)	B3LYP (ppm)	(3)	B3LYP (ppm)	(4)	B3LYP (ppm)
51H	12.87	10H	8.88	10H	8.87	43H	11.34
31H	9.39	19H	8.79	19H	8.82	10H	8.87
10H	8.98	48H	8.67	40H	8.64	19H	8.76
19H	8.67	29H	8.49	29H	8.42	29H	8.34
29H	8.43	31H	8.00	31H	7.93	31H	8.28
41H	8.39	13H	7.84	13H	7.82	13H	7.90
48H	8.06	23H	7.83	23H	7.80	23H	7.85
47H	8.05	14H	7.63	14H	7.62	14H	7.68
13H	7.97	25H	7.62	8H	7.60	25H	7.66
23H	7.89	8H	7.57	25H	7.59	41H	7.58
46H	7.83	41H	7.44	38H	7.37	8H	7.57
14H	7.64	24H	7.35	24H	7.33	44H	7.46
8H	7.64	12H	7.31	12H	7.31	24H	7.37
25H	7.63	40H	7.29	21H	7.20	40H	7.37
49H	7.62	21H	7.21	43H	6.87	12H	7.33
44H	7.55	42H	6.83	42H	6.74	21H	7.25
24H	7.40	46H	4.11	49H	3.41	45H	6.99
21H	7.36	47H	3.71	46H	3.40		
12H	7.28	45H	3.71	47H	3.08		
				51H	3.06		
				45H	2.75		
				50H	2.71		

Since these hydrazones are structurally similar regarding the triazine skeleton therefore most of the chemical shifts appear similar in the NMR spectra. However, distinct differences have been observed in the chemical shifts arising from differences in the hydrazomethyl fragment. For example, the naphthalene moiety in **1** can easily be identified by a downfield chemical shift at 9.39 ppm (31H) which arises due to peri-hydrogens. Moreover, increased number of downfield chemical shifts between 8–9 ppm also supported the naphthalene part of compound **1**. The *ortho* hydroxyl groups in compound **1** and **4** were supported by downfield chemical shifts at 12.77 ppm (1) and 11.47 ppm (4) (*vide supra*). The characteristic methoxy group of compound **2** appeared at 3.79 ppm in the experimental NMR spectrum which correlates nicely with the theoretical chemical shift at 4.11–3.71 ppm. Similarly, the characteristic dimethyl amino group in 3 was confirmed by chemical shift at 3.1 ppm, corresponding to six protons.

### 2.6. Frontier Molecular Orbital Analysis (FMO) and UV-Vis. Absorption Studies

FMO analysis is a physical property used to determine, ability to absorb light, electronic as well as optical properties of organic compounds [16]. In molecular interaction, the highest occupied molecular orbital (HOMO) and lowest unoccupied molecular orbital (LUMO) play the key role. HOMO is the orbital which has ability to donate electrons and its energy corresponds to ionization potential (I. P.), while LUMO has electrons accepting ability, and its energy corresponds to electron affinity (E. A.).

**Figure 6:**
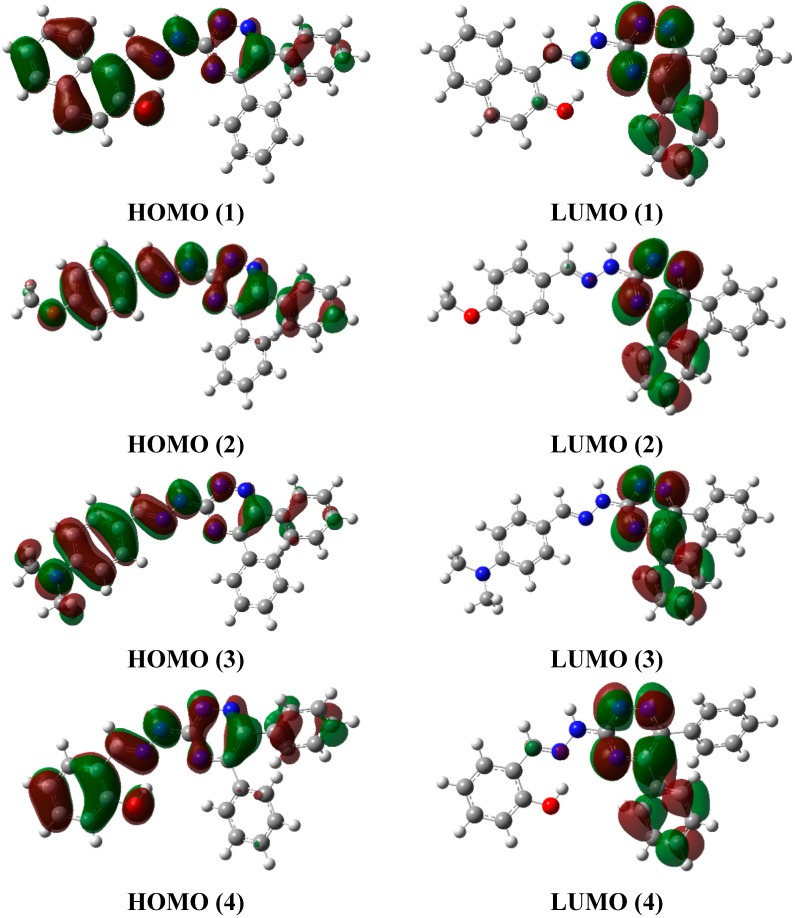
HOMO, LUMO surfaces of compounds **1**–**4** simulated at B3LYP/6-31G (d, p).

FMO analysis was computed at the same level as used for optimization along with additional keyword pop = full, and HOMO-LUMO surfaces of all four molecules **1**–**4** shown in Figure 6 and energies are listed in the Table 9. The FMO analysis showed that the HOMO is mainly concentrated on the triazine moiety, azomethine and aromatic ring attached to the azomethine moiety. The LUMO is mainly concentrated on the triazine and a phenyl ring attached to it. The HOMO-LUMO energy gap for **4** was the highest and equal to 0.133 eV and for **3** it was the lowest and equal to 0.119 a. u. (3.24 eV). The isodensities of HOMO and LUMO not only help explain the HOMO-LUMO gap but also deliver useful information regarding the stability of a compound. The isodensity in the HOMO gives a maximum spread in compound **4** (it also encompasses a benzene ring) which reflects the higher conjugation in **4**. These observations indicate the HOMO in **4** should have the lowest energy among all four compounds studied, and indeed this is the case. The extended conjugation in **4** would render it more stability towards ionization (*vide infra*). On the other hand, HOMO is **3** has the minimum density on the hydrazone scaffold which renders it the compound with the highest energy (0.18 eV). The higher energy of HOMO in **3** is expected due to the presence of a dimethylamino group. The situation is slightly different for the LUMOs; the extent of conjugation in LUMO in all four compounds is similar (Figure 6). However, the intensities are slightly different. Relatively higher intensities are observed for compounds **1** and **4** which render them of lower energy compared to the LUMO in compounds **2** and **3**. It is interesting to note that the compounds **1** and **4** have intramolecular hydrogen bonding. It can be argued that intramolecular hydrogen bonding provides low energy to the LUMO. The HOMO-LUMO gap is the sum of the effect on HOMO and LUMO. For example, in compound **4**, both HOMO and LUMO are stabilized but the effect on the HOMO is larger than on the LUMO, and it ultimately results in an increased band gap. On the other hand, both HOMO and LUMO are destabilized in **3** but, the effect on the former is more pronounced than the latter, and it ultimately leads to a decrease in the band gap.

**Figure 7:**
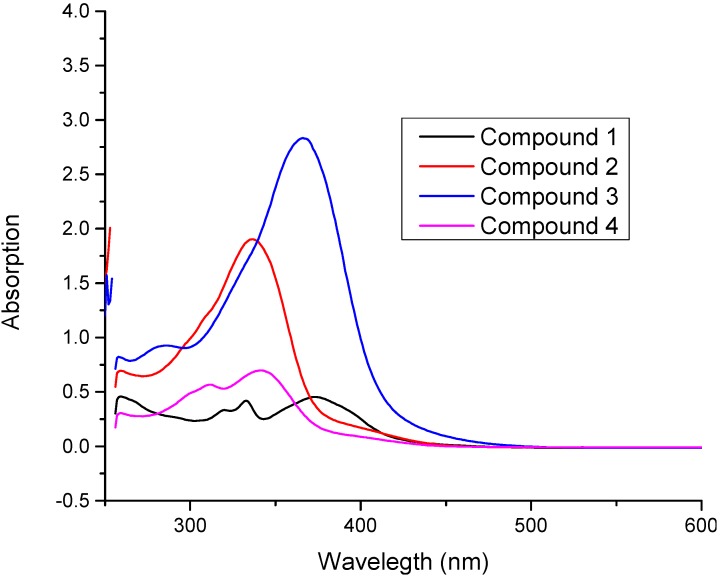
Combined experimental UV-vis. spectra of all compounds **1**–**4**.

The experimental UV–Vis. absorption spectra of all compounds **1**–**4** were recorded within 200–600 nm range in dimethyl sulphoxide (DMSO), and the combined spectra are shown in Figure 7.

The theoretical absorption studies were also carried out by using TD-DFT method at B3LYP/6-31G (d, p) level of theory in gas phase, and polarizable continuum model (PCM) was applied to account for solvent effect (For simulated UV-Vis. spectra see Figure S9 and Figure S10). A comparison of characteristic experimental and simulated UV–vis. absorption wavelengths (λ_max_) of **1**–**4** is given in Table 10.

**Table 9:** Frontier orbital energy (a. u).

Compound	E (HOMO)	E (LUMO)	ΔE (LUMO‒HOMO)
**1**	−0.196	−0.0713	0.124
**2**	−0.196	−0.064	0.132
**3**	−0.18	−0.060	0.119
**4**	−0.204	−0.072	0.133

The experimental UV-vis. spectrum of compound **1** showed an absorption maximum at 374 nm, which is in excellent agreement with the computed values of 380 nm in the gas phase and 390.3 nm in DMSO. The experimental absorption maxima of **2** appeared at 337 nm, and shows excellent correlation with simulated values at 354.2 nm (gas phase) and 358.2 nm (DMSO). Similarly the theoretical and experimental absorption maxima’s of **3** and **4** showed very excellent correlations (for individual values see Table 10). The observed absorption maxima of compounds **1**–**4** can also be correlated with the HOMO-LUMO band gap. Compounds **2** and **4** have the highest band gaps (Table 9) and this is consistent with the UV results that these compounds have the highest energy of absorption (shorter absorption maximum). The band gap in compound **4** is 0.13 a. u. which corresponds to 350 nm (3.536 eV). This is identical to theoretical absorption maximum of compound **4** at 350.2 nm. Therefore, it can be concluded that the π‒π* transition in these compounds is a HOMO-LUMO transition. It is also interesting to note that compounds having intra-molecular hydrogen bonding (**1** and **4**) have the low molar absorptivity (Figure 7).

**Table 10:** Experimental and simulated (B3LYP/6-31G (d, p)) UV-vis. λ_max_ (nm) values of title compounds **1**–**4**.

Experimental		Theoretical (TD-SCF/B3LYP/6-31G (d, p))		
Compound	λ_max_ (abs.) (DMSO)	λ_max_ (osc. Strength), Gas Phase	λ_max_ (osc. Strength) DMSO	Assignment
(1)	333 (0.419), 374 (0.454)	380.8 (0.782)	390.3 (0.945)	π‒π*
(2)	337 (1.903)	345.2 (1.189)	358.2 (1.401)	π‒π*
(3)	366 (2.834)	370.4 (1.147)	395.5 (1.196)	π‒π*
(4)	312 (0.567), 342 (0.697)	350.2 (0.739)	356.1 (1.007)	π‒π*

### 2.7. Molecular Electrostatic Potential (MEP)

One of the most interesting features of quantum chemistry is the ability to explain the reactivity of compounds under investigation. In terms of reactivity the electrostatic potential plays an important role. It determines the reactivity of a chemical system by predicting electrophilic as well as nucleophilic sites in target molecules [17]. This is one of the basic properties, which affect the behavior of a whole target molecule [18]. Mathematically, MEP can be defined by the following equation:

(1) $$V(r)=\sum (Z_{A}R_{A}-r)\int (\rho (r´)/r´-r)dr´$$

**Figure 8:**
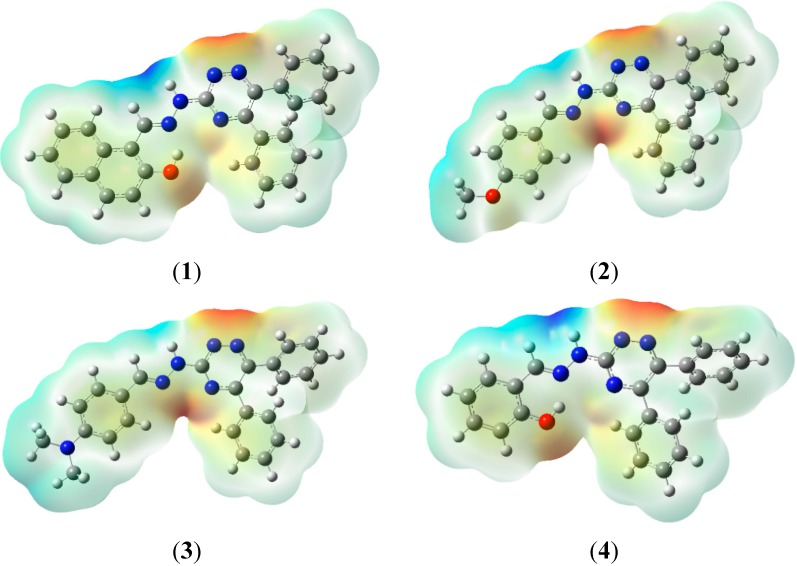
MEP surfaces of compounds **1**–**4**.

Summation (∑) runs over all nuclei, Z*_A_* is charge of nucleus which is located at R_A_ and ρ(r') is electron density. MEP is also proved very useful in structural biology to determine ligand-substrate interactions, change in energy upon interactions and determination of local reactivity of large target molecules under investigation, drug receptor and in enzyme-substrate interactions. The molecular electrostatic potential of compounds **1**–**4** was computed at the B3LYP/6-31G (d, p) level of DFT and the corresponding surfaces are shown in Figure 8.

During MEP mapping two basic regions appear *i.e.*, red and blue, the preferred nucleophilic site is represented by red color and the preferred electrophilic site is represented by blue color. According to the MEP analysis of compound **1**, a negative potential is −5.243 × 10^−2^ concentrated on the oxygen of the -OH group attached to the naphthalene moiety and on nitrogens of the triazine ring (N_27_, N_28_), this is the preferred site for electrophilic attack as well as for metal atoms. Positive potential reflecting a nucleophilic site was 5.243 × 10^−2^ and was concentrated on the proton attached to N_32_ and on the proton of the azomethine moiety (–C_32_=N_33_). For compound **2**, the negative potential is −5.691 × 10^−2^ concentrated on the triazine ring, whereas the corresponding positive potential value is 5.691 × 10^−2^. An almost similar trend was observed during the MEP analysis of **3**, having a negative potential value −6.094 × 10^−2^ and positive value of 6.094 × 10^−2^. Compound **4** showed a negative potential value of −5.240 × 10^−2^ concentrated on the oxygen of the -OH and the nitrogens of the triazine ring (N_27_, N_28_). The value of its positive potential is 5.240 × 10^−2^, concentrated on the protons attached to the azomethine moiety (–C_32_=N_33_) and showing the nucleophilic site.

Very similar to the other properties mentioned above, compounds **1**–**4** can be divided into two distinct groups based on MEP: (a) with intramolecular hydrogen bonding (b) without intramolecular hydrogen bonding. The intramolecular hydrogen bonding confers to the oxygen of the hydroxyl group a negative potential and also makes it a suitable site for electrophilic attack. The more pronounced effect in compound **1** and **4** is observed for proton attached to N4 (the proton becomes more acidic). These analyses clearly reveal that small changes in the structure can lead to dramatic changes in the reactivity

### 2.8. Hyperpolarizability and Non-Linear Optical Properties

Molecules having asymmetric polarization because of the presence of electron donor and electron acceptor groups in a π-conjugated system are strong candidates for NLO applications. Materials with high NLO properties are vital in optoelectronic and non-linear optics and have great effect in information technology and other industries [19]. With the advancements in laser technology, NLO materials are used as important materials for photonic communication and digital memory, in industry, national defense and medicine [20]. In order to establish the relation between molecular structure and NLO properties, first the hyperpolarizibility of compounds **1**–**4** was computed at the DFT- B3LYP/6-31G (d, p) level of theory along with additional key word POLAR and mathematically calculated by following Equation (2):

β_tot_ = [(β_xxx_ + β_xyy_ + β_xzz_)^2^ + (β_yyy_ + β_yzz_ + β_yxx_)^2^ + (β_zzz_ + β_zxx_ + β_zyy_)^2^]^1/2^ (2)

The value of first hyperpolarazibility appears in a. u. and converted to e.s.u using Equation (2) a. u. = 8.6393 × 10^−33^ e. s. u. The calculated first hyperpolarizibility parameters of compounds **1**–**4** are listed in Table 11.

**Table 11:** First hyperpolarizability parameters of **1**–**4**.

Compound	1	2	3	4
β_xxx_	2300.433	3639.217	8152.091	1281.031
β_xxy_	6.139	41.559	−559.94	223.445
β_xyy_	−520.79	207.2331	−62.360	−303.73
β_yyy_	−244.06	−402.645	−357.94	−459.49
β_xxz_	584.743	17.3065	−46.037	40.0394
β_xyz_	33.763	64.606	94.261	42.973
β_yyz_	1.283	−3.485	−15.970	0.236
β_xzz_	39.428	1.523	−8.302	34.723
β_yzz_	14.681	4.147	37.649	17.0493
β_zzz_	9.201	9.715	−8.129	7.664
**β_tot._ (esu)**	**16.647**	**29.797**	**70.233**	**8.957**

Organic molecules having extended π-conjugation systems and electron donating groups possess higher NLO properties. As reflected from Table 11, the values of the first hyperpolarizibility show the same trend with as electron donating capacity and extended π-conjugation pattern, that is **3** > **2** > **1** > **4**. Moreover, the first hyperpolarizability can also be correlated to the band gap. Compound **4** (with the highest band gap) has the lowest hyperpolarizability. On the other hand, the easy flow of electrons from one terminus of molecule to the other renders a lower hyperpolarizability value in compound **3**. From the Table, it is clear that **3** has the highest value compared to others and can be a potential NLO candidate when compared to **1**, **2** and **4**.

### 2.9. Chemical Reactivity

Global chemical reactivity indices such as total energy, chemical hardness (η), electrophilicity (ω), electronic chemical potential, and dipole moment (µ) are used to describe the reactivity as well as stability of any chemical compounds [21]. The chemical hardness can be defined in term of following equation (3):

η = (E_HOMO_ − E_LUMO_)/2 (3)

Using above equation chemical hardness of compound **1**–**4** were calculated and listed in Table 12 along with other parameters.

**Table 12:** Reactivity indices of compounds **1**–**4**.

Properties	1	2	3	4
E_Total_ (eV)	0.124	0.132	0.119	0.133
E_HOMO_	−0.196	−0.196	−0.18	−0.204
E_LUMO_	−0.0713	−0.064	−0.060	−0.072
η (eV)	0.062	0.066	0.059	0.066
µ (eV)	−0.133	−0.13	−0.120	−0.138
ω (eV)	0.142	0.128	0.119	0.143
µ_(Debye)_	1.01	3.12	5.37	0.57

Compounds having a high HOMO-LUMO energy gap are stable and chemically harder than compounds having a small HOMO-LUMO energy gap. From Table 12 it is clear that **4** is hard and more stable (less reactive), while **3** is soft and least stable of all (more reactive). The electrophilicity index (ω) is based on thermodynamic properties and measures the favorable change in energy when a chemical system attains saturation by addition of electrons. It can be defined as the decrease in energy due to flow of electrons from the HOMO to the LUMO in molecules. It also plays an important role in determining the chemical reactivity of system and mathematically defined by the following equation (4) [22]:

ω = µ^2^/2η (4)

where, µ is the electronic chemical potential, η is chemical hardness. Results from Table 12 show that **4** is strong electrophilic while **3** is nucleophilic in nature. Electronic chemical potential (µ) describes the charge transfer within a system in the ground state. It is define as the negative of the electronegitivity [23] and defined mathematically by the following equation (5):

µ = (E_HOMO_ + E_LUMO_)/2 (5)

Physically it is defined as the tendency of electrons to escape from the equilibrium state. Compounds having greater values of chemical potential are most reactive than ones with small electronic chemical potential. From the Table 12, it is clear that **3** is most reactive while **4** is least reactive of all.

## 3. Experimental

### 3.1. General Information

All chemicals and solvents were purchased from BDH (Poole, UK), and used without further purification. A Stuart Scientific SMP3, version 5.0 melting point apparatus (Bibby Scientific Limited, Staffordshire, UK) was used to record the melting points, and the reported m.p. are uncorrected. ^1^H-NMR spectra were recorded on an AVANCE-III 600 MHz instrument (Bruker, Fallanden, Switzerland) at 300 K, and chemical shifts are reported in ppm with reference to the residual solvent signal. FT-IR spectra were recorded neat conditions on a Thermo Scientific NICOLET iS 50 FT-IR spectrometer (Thermo Scientific, Madison, WI, USA). UV-vis. studies were performed by using Evolution 300UV/VIS spectrophotometer (Thermo Scientific).

### 3.2. Synthesis of 5,6-Diphenyl-4H-[1,2,4]triazine-3-thione

Benzil (6 g, 28.5 mmol) was dissolved in glacial acetic acid (150 mL) and added to a solution of thiosemicarbazide (2.59 g, 28.5 mmol) in hot water (100 mL). The mixture was refluxed for 4 h, and the precipitate that appeared was filtered while the solution was hot. The orange crystals obtained were recrystallized from ethanol to give reddish crystals (yield: 5.27 g, 87.82%, m.p. 222–224 °C).

### 3.3. Synthesis of 3-Hydrazinyl-5,6-diphenyl-1,2,4-triazine

A mixture of 5,6-diphenyl-4*H*-[1,2,4]triazine-3-thione (5 g, 18.84 mmol) and hydrazine hydrate (10 mL) in isopropyl alcohol (20 mL) was refluxed for 4–6 h, until no more H_2_S was evolved. Acetic acid was added dropwise into mixture till neutralization to remove the excess of hydrazine. The mixture was cooled. The solid obtained was filtered off and crystalized from ethanol to give yellowish crystals (84% yield, m.p. 175–178 °C).

### 3.4. Synthesis of Compounds **1**–**4**

In an oven dried flask containing ethanol (15 mL), 3-hydrazinyl-5,6-diphenyl-1,2,4-triazine (0.1 g, 0.379 mmol) and the respective aldehyde (0.379 mmol) were mixed together. The mixture was refluxed for 2 h with stirring and then cooled. Reaction progress was monitored by TLC. The precipitates obtained were then filtered off to yield the final products.

*(Z)-((2-(5,6-Diphenyl-1,2,4-triazin-3-yl)hydrazono)methyl)naphthalene-2-ol* (**1**): IR (neat, cm^−1^): ν = 756, 1062, 1179, 1239, 1280, 1367, 1517, 1611, 2810; ^1^H-NMR (DMSO, ppm): 12.77, (1H, s, OH,), 12.31 (1H, b, NH), 9.33 (1H, s, =CH-), 8.22 (1H, d, Ar-H, *J* = 8.4 Hz), 7.90 (2H, dd, Ar-H, *J* = 6.0 Hz, 4.2 Hz), 7.61 (1H, t, Ar-H, *J* = 8.4 Hz), 7.52 (2H, d, Ar-H, *J* = 7.2 Hz), 7.47 (1H, d, Ar-H, *J* = 7.8 Hz, 7.2 Hz), 7.38–7.44 (8H, m, Ar-H), 7.31 (1H, d, Ar-H, *J* = 9 Hz); ^13^C-NMR (DMSO, ppm): 157.28, 156.47, 142.87, 136.06, 135.81, 131.94, 131.29, 130.50, 129.45, 129.08, 128.95, 128.56, 128.33, 127.82, 127.62, 123.49, 120.58, 118.95, 108.97, UV-vis. λ_max_ (DMSO) = 333 nm, 374 nm,; m.p. 310–312 °C.

*(Z)-3-(2-(4-Methoxybenzylidene)hydrazinyl)methyl)-5,6-diphenyl-1,2,4-triazine* (**2**): IR (neat, cm^−1^): 764, 1060, 1167, 1247, 1350, 1513, 1612; ^1^H-NMR (DMSO, ppm): 11.80, (1H, b, NH,), 8.22 (1H, s, =CH-), 7.66 (2H, d, Ar-H, *J* = 9 Hz), 7.46 (3H, dd, Ar-H, *J* = 7.8 Hz, 7.2 Hz), 7.36 (7H, m, Ar-H), 7.00 (2H, d, Ar-H, *J* = 9 Hz), 3.77 (3H, s, CH_3_); ^13^C-NMR (DMSO, ppm): 160.35, 158.56, 156.36, 150.59, 143.96, 136.17, 135.99, 130.20, 129.41, 128.92, 128.36, 128.26, 128.24, 128.17, 127.32, 114.31, 55.25; UV-vis. λ_max_ (DMSO) = 337 nm; m.p. 267–268 °C.

*(Z)-4-((2-(5,6-Diphenyl-1,2,4-triazin-3-yl)hydrazono)methyl)-N,N-dimethylaniline* (**3**): IR (neat, cm^−1^): 516, 807, 1052, 1160, 1277, 1363, 1428, 1507, 1593; ^1^H-NMR (DMSO, ppm): 11.62, (1H, b, NH), 9.65 (1H, s, =CH-), 7.53 (2H, d, Ar-H, *J* = 9 Hz), 7.45 (3H, dd, Ar-H, *J* = 7.8, 7.2 Hz), 7.35–7.38 (7H, m, Ar-H), 6.75 (2H, d, Ar-H *J* = 9 Hz), 2.96 (6H, s, CH_3_); ^13^C-NMR (DMSO, ppm): 189.89, 156.28, 154.21, 151.13, 150.18, 145.07, 136.05, 130.16, 129.38, 128.96, 128.29, 128.25, 128.23, 127.95, 124.48, 122.13, 111.87, 111.05; UV-vis. λ_max_ (DMSO) = 366 nm; m.p. 263–265 °C.

*(Z)-2-((2-(5,6-Diphenyl-1,2,4-triazin-3-yl)hydrazono)methyl)-phenol* (**4**): IR (neat, cm^−1^): 688, 750, 1062, 1078, 1133, 1279, 1375, 1515, 1621; ^1^H-NMR (DMSO, ppm): 12.26, (1H, b, NH,), 11.47, (1H, s, OH,), 8.45 (1H, s, =CH-), 7.48 (3H, d, Ar-H, *J* = 7.8 Hz), 7.45 (1H, dd, Ar-H, *J* = 7.8 Hz, 7.2 Hz), 7.41–7.42 (2H, m, Ar-H), 7.36–7.49 (5H, m, Ar-H), 7.26 (1H, t, Ar-H *J* = 8.4 Hz), 6.90–6.94 (2H, m, Ar-H); ^13^C-NMR (DMSO, ppm): 158.08, 157.13, 156.44, 151.14, 145.00, 136.05, 135.80, 130.64, 130.43, 129.46, 129.06, 128.52, 128.29, 119.30, 118.88, 116.39; UV-vis. λ_max_ (DMSO) = 312 nm, 342 nm; m.p. 273–274 °C.

### 3.5. Crystallography

Compounds were crystalized in dimethyl sulfoxide (DMSO) while keeping their solution at room temperature *i.e.*, 25 °C for about three months. Suitable crystals were selected under microscope and fixed on glass tip using glue supported by copper tube and magnetic base. These were mounted on an Agilent SuperNova (dual source) diffractometer (Agilent Technologies, Santa Clara, CA, USA) equipped with graphite-monochromatic Cu/Mo Kα radiation for data collection. The data collection was accomplished using CrysAlisPro software [24] at 296 K under the Mo Kα radiation. The structure solution was performed using SHELXS–97 and refined by full–matrix least–squares methods on F^2^ using SHELXL–97 [25], in-built with X-Seed [26]. All non–hydrogen atoms were refined anisotropically by full–matrix least squares methods [25]. The C-H hydrogen atoms were positioned geometrically and treated as riding atoms where C–H = 0.93 Å with Uiso(H) = 1.2 Ueq(C) for aromatic carbon atoms and C–H = 0.96 Å with Uiso(H) = 1.5 Ueq(C) for methyl carbon atoms. H atoms bonded to N in all four compounds were located in a difference maps and refined isotropically 0.89 (1) for N-H using DFIX commands. The O-H hydrogen atoms in **1** & **4** were refined geometrically with O-H = 0.82 and Uiso(H) = 1.5 Ueq(O) for oxygen atom. The figures were drawn using ORTEP-3 [27] and PLATON [28] programs inbuilt with WINGX [27]. The cifs for these compounds have been submitted to CCDC to obtain CCDC numbers (1028227, 1028228, 1028229 and 1034522 for compounds **1**–**4** respectively). These data files can be obtained free of charges on application to CCDC 12 Union Road, Cambridge CB21 EZ, UK. (Fax: (+44) 1223 336-033; e-mail: data_request@ccdc.cam. ac.uk).

### 3.6. Computational Details

Theoretical studies were performed using the Gaussian 09 software at the density functional theory (DFT) level as instituted in the program [29]. The visualization of the results/optimized geometries was achieved by using Gauss View 05 [30]. The energy minima optimization of all compounds was carried out at B3LYP/6-31G (d, p) levels of theory. The B3LYP method provides a nice balance between cost and accuracy, and it is known to perform very well for the prediction of geometries of a number of synthetic [31,32] and natural products [33,34]. Frequency simulations were performed at the same level, to confirm the optimized geometries as a true minimum (no imaginary frequency was observed). In addition, frequency simulations at B3LYP/6-311G (d, p) level were used for vibrational analysis. Nuclear magnetic resonance studies were performed at B3LYP/6-311+G (2d, p) level, by adopting GIAO method. Chemical shift values were referred by using the internal reference standard *i.e.*, tetramethylsilane. UV-vis. absorption studies were simulated by using TD-DFT method and at B3LYP/6-31G (d, p) level of theory. MEP, FMO and first hyperpolarizability analyses were simulated at B3LYP/6-31G (d, p) level of DFT.

## 4. Conclusions

In this study, four triazine-based hydrazone derivatives have been synthesized in good yields, and characterized using FT-IR, UV-Vis., ^1^H- and ^13^C-NMR spectroscopic techniques. The structures of these hydrazones were also confirmed unequivocally by single crystal X-ray diffraction studies. The DFT studies showed a strong agreement between the simulated and experimental results. Intra-molecular hydrogen bonding in compounds **1** and **4** results in a decrease in certain bond lengths in the triazine core. The structural variation in the hydroazamethyl part has a significant effect on the electronic properties of compounds **1**–**4**. The molar absorptivity of transitions in the UV-Vis spectra (compounds **1** and **4**) is considerably decreased by the intramolecular hydrogen bonding. The absorption at the longest wavelength in the UV-Vis spectrum is a π–π* transition from the HOMO to the LUMO, and correlates with the calculated HOMO-LUMO band gap. Frontier molecular orbital analysis showed that compounds **1** and **3** have very low HOMO-LUMO energy gaps, and therefore are kinetically less stable. The molecular electrostatic potential investigations revealed that the electronegative region in all compounds was spread over the triazine moiety. Chemical reactivity indices predict the highest and lowest activity for **3** and **4**, respectively. The lowest band gap is calculated for compound **3**, which gives it interesting electronic properties. The first hyperpolarizability analysis of all compounds was performed and compound **3** again showed the highest value compared to **1**, **2** and **4**. This indicates that **3** may have a very good nonlinear optical response. Triazine-based hydrazone derivatives have very wide applications not only in the clinical field but also in other areas of chemistry and hopefully the results of this study will increase the interest of researchers working in this field.

## Supplementary Materials

Cartesian co-ordinates of optimized geometries and cif files of all compounds **1**–**4** are given in supporting information. Experimental ^1^H-, ^13^C-NMR are also provided in the Supporting Information along with simulated UV-vis. spectra. Supplementary materials can be accessed at: http://www.mdpi.com/1420-3049/20/04/5851/s1.
