# Supplementary material for: Synthesis, Crystal Structures and Spectroscopic Properties of Triazine-Based Hydrazone Derivatives; A Comparative Experimental-Theoretical Study
Source: Molecules. 2015 Apr 3;20(4):5851–74. doi: 10.3390/molecules20045851 (PMC6272468; doi:10.3390/molecules20045851)
Supplement: Supplementary file 1 [file molecules-20-05851-s001.pdf]

Supplementary Materials

CartesianCo−ordinates of optimized geometry at B3LYP/6−31G (d, p) (1)

C	3.19696900	−1.18947100	−0.11479500
C	2.33950900	−0.05164900	−0.16975400
C	0.59856500	−1.50038200	−0.30643000
C	2.78752700	1.36261900	−0.23987600
C	3.95114700	1.73798200	−0.93139500
C	1.98366100	2.36206800	0.33313900
C	4.30492300	3.08110900	−1.03612200
H	4.56977600	0.98124500	−1.39986500
C	2.35039300	3.70227700	0.24078200
H	1.07112200	2.07482100	0.84343500
C	3.51180600	4.06586600	−0.44369400
H	5.20024200	3.35867900	−1.58418000
H	1.72512000	4.46342400	0.69760100
H	3.79455800	5.11168900	−0.52051900
C	4.65206700	−1.17580900	0.18198500
C	5.51081400	−2.04730800	−0.50675000
C	5.18399100	−0.36263000	1.19587700
C	6.87043100	−2.08726300	−0.20393300
H	5.09699000	−2.69340400	−1.27320200
C	6.54296100	−0.40907500	1.50008200
H	4.53038100	0.30052900	1.75305700
C	7.39187600	−1.26729500	0.79846300
H	7.52323800	−2.76132900	−0.75085100
H	6.93771500	0.22158000	2.29121900
H	8.45135200	−1.30013500	1.03501100
N	1.01704000	−0.23682000	−0.21723000
N	1.37988100	−2.58962700	−0.46653400
N	2.67810600	−2.41301700	−0.32840000
H	−1.01266900	−2.74938800	−0.38051600
C	−2.92449600	−1.11360300	−0.12704800
H	−3.21999700	−2.16274300	−0.20490000
N	−0.74499500	−1.77426200	−0.27995700
N	−1.66932600	−0.79652000	−0.15581500
C	−3.95181400	−0.09899800	0.00753500
C	−5.35210500	−0.45602600	0.04782300
C	−3.58936400	1.26055600	0.10290500
C	−5.82548000	−1.79337500	−0.03700100
C	−6.33475600	0.57862900	0.18139000
C	−4.58220800	2.26595800	0.23421300
C	−7.17296400	−2.08695900	0.00646800

H	−5.12749400	−2.61655400	−0.13766400
C	−7.71205400	0.24621700	0.22287800
C	−5.90847400	1.93344700	0.27185300
H	−4.24557500	3.29479900	0.30181700
C	−8.13307600	−1.06173200	0.13743700
H	−7.49598900	−3.12181200	−0.06113300
H	−8.43412800	1.05235700	0.32461200
H	−6.66185100	2.71017300	0.37236400
H	−9.19042000	−1.30519700	0.17036100
O	−2.31975800	1.69150800	0.07643300
H	−1.71834000	0.90999600	−0.02875200

CartesianCo−ordinates of optimized geometry at B3LYP/6−31G (d, p) (2)

C	2.97758400	−1.06480900	−0.09537300
C	2.00152200	−0.02876400	−0.15747600
C	0.42175000	−1.65181800	−0.26638600
C	2.29672100	1.42541900	−0.24219000
C	3.39498300	1.92055700	−0.96353700
C	1.40729700	2.33481900	0.35282600
C	3.60249400	3.29365700	−1.07506400
H	4.07769000	1.23259800	−1.44922900
C	1.62851000	3.70633100	0.25465600
H	0.54415900	1.94850900	0.88354000
C	2.72652200	4.19004900	−0.45948800
H	4.44898800	3.66348100	−1.64586500
H	0.94003500	4.39847000	0.73046800
H	2.89593500	5.25978500	−0.54099400
C	4.42430900	−0.89107000	0.19121200
C	5.36809800	−1.68409600	−0.48115900
C	4.87273500	−0.00090900	1.18046700
C	6.72560600	−1.57267600	−0.18689900
H	5.02178700	−2.39013800	−1.22797300
C	6.23034700	0.10482800	1.47589800
H	4.15529700	0.60311100	1.72605700
C	7.16250200	−0.67647100	0.79044800
H	7.44338600	−2.18826500	−0.72135700
H	6.55915600	0.79412400	2.24814100
H	8.22059700	−0.59109000	1.02007300
N	0.70595200	−0.35014300	−0.19525400
N	1.31879700	−2.65316100	−0.41897300
N	2.59064900	−2.33934600	−0.29159500

H	−1.00728200	−3.08845300	−0.32895800
C	−3.10493300	−1.77478900	−0.13127900
H	−3.23685800	−2.86304800	−0.21367200
N	−0.87856100	−2.08342300	−0.23101400
N	−1.93255400	−1.24324100	−0.12873300
C	−4.31923400	−0.97297700	−0.02781900
C	−5.56954600	−1.60360300	−0.02722200
C	−4.28137000	0.43414800	0.07092300
C	−6.75572300	−0.87470200	0.06817100
C	−5.44944500	1.16741600	0.16610200
C	−6.69875800	0.51990100	0.16571200
H	−5.43100400	2.24966400	0.24124700
H	−5.62205800	−2.68703500	−0.10272300
H	−7.70431700	−1.39760000	0.06539900
O	−7.78448500	1.33702700	0.26308100
C	−9.07539300	0.74585000	0.26445200
H	−9.20874500	0.06843200	1.11734300
H	−9.78381100	1.57104100	0.34719600
H	−9.26874300	0.19548300	−0.66497600
H	−3.31778200	0.93216900	0.06876700

CartesianCo−ordinates of optimized geometry at B3LYP/6−31G (d, p) (3)

C	3.33597000	−1.04160900	−0.08827100
C	2.32521000	−0.04079300	−0.15910700
C	0.80016200	−1.71692300	−0.25983700
C	2.57097100	1.42248800	−0.25338100
C	3.65237500	1.95027900	−0.97687100
C	1.65039600	2.30506000	0.33439100
C	3.81324200	3.32893800	−1.09734100
H	4.35884400	1.28287600	−1.45728900
C	1.82473800	3.68271400	0.22718200
H	0.79989300	1.89275300	0.86596800
C	2.90651300	4.19913900	−0.48884000
H	4.64748400	3.72348000	−1.66970800
H	1.11226500	4.35403300	0.69761100
H	3.03953500	5.27349900	−0.57725700
C	4.77538500	−0.81768000	0.19963500
C	5.74649700	−1.58419700	−0.46481000
C	5.19299300	0.09463800	1.18220800
C	7.09908400	−1.42553000	−0.16969000
H	5.42491800	−2.30754200	−1.20610500

C	6.54596200	0.24821600	1.47818700
H	4.45504800	0.67834000	1.72253700
C	7.50478000	−0.50718900	0.80053800
H	7.83764900	−2.02151500	−0.69810700
H	6.85036700	0.95445600	2.24517100
H	8.55913100	−0.38479300	1.03075600
N	1.04136600	−0.40508300	−0.19829700
N	1.73349900	−2.68818200	−0.40587700
N	2.99253900	−2.33013900	−0.27862900
H	−0.57574400	−3.20121400	−0.31434900
C	−2.71834100	−1.96866600	−0.12744500
H	−2.80075900	−3.06259800	−0.20588100
N	−0.48193200	−2.19184300	−0.22177800
N	−1.56820100	−1.38797800	−0.12467600
C	−3.96726900	−1.22781300	−0.02975200
C	−5.19222500	−1.91261200	−0.03359300
C	−4.00789300	0.17600800	0.06316900
C	−6.40517500	−1.24306100	0.05462700
H	−5.19693300	−2.99749700	−0.11173100
C	−5.20904300	0.85760700	0.15270600
H	−3.07250400	0.72590800	0.05826400
C	−6.44814800	0.16669100	0.16071700
H	−7.32095900	−1.82030600	0.04008000
H	−5.18830000	1.93847700	0.21469500
C	−7.66726900	2.29774300	0.22333800
H	−8.69242500	2.64662700	0.35304700
H	−7.06629800	2.73069600	1.03152800
H	−7.28869000	2.69497300	−0.73044800
C	−8.90035400	0.12301100	0.12520600
H	−8.99411000	−0.66715200	0.87961400
H	−9.73208300	0.81393600	0.26731800
H	−9.00655500	−0.34191300	−0.86659400
N	−7.64886300	0.84547100	0.27398400

CartesianCo−ordinates of optimized geometry at B3LYP/6−31G (d, p) (4)

C	2.33411600	−1.13082900	−0.11277400
C	1.36791600	−0.08347600	−0.16627100
C	−0.22262500	−1.69677700	−0.27897700
C	1.67422800	1.36742200	−0.24721200
C	2.77808800	1.85213500	−0.96789600
C	0.79149800	2.28521100	0.34571600

C	2.99683500	3.22303000	−1.08155600
H	3.45532100	1.15797800	−1.45226200
C	1.02414100	3.65452500	0.24476400
H	−0.07595600	1.91192400	0.87857200
C	2.12701000	4.12750100	−0.46889500
H	3.84686200	3.58488400	−1.65209400
H	0.33950800	4.35237300	0.71724600
H	2.30478600	5.19563300	−0.55280700
C	3.78292500	−0.97037000	0.17134100
C	4.71860800	−1.76567200	−0.50930400
C	4.23925900	−0.09137300	1.16687100
C	6.07743800	−1.66711100	−0.21656300
H	4.36569100	−2.46282400	−1.26135100
C	5.59804800	0.00076500	1.46107700
H	3.52760000	0.51440800	1.71805900
C	6.52259600	−0.78246900	0.76752400
H	6.78953600	−2.28378400	−0.75718200
H	5.93373200	0.68100700	2.23827000
H	7.58165100	−0.70739300	0.99610800
N	0.06976800	−0.39784000	−0.19972800
N	0.66028600	−2.70480900	−0.43797400
N	1.93645400	−2.40087900	−0.31244800
H	−1.70283300	−3.09955400	−0.34277800
C	−3.76503400	−1.65600200	−0.09715800
H	−3.98847200	−2.72678900	−0.18127500
N	−1.53343800	−2.10242200	−0.24376700
N	−2.54894500	−1.22138200	−0.11910700
C	−4.88434700	−0.74286500	0.04263000
C	−6.19635300	−1.25410500	0.06415800
C	−4.69486100	0.66243100	0.16276200
C	−7.29692600	−0.42086800	0.20037900
C	−5.81341500	1.49637400	0.30085400
C	−7.09554700	0.96083200	0.31944600
H	−5.64343800	2.56404500	0.39049100
H	−7.94826500	1.62501800	0.42690800
O	−3.47823900	1.23777000	0.15006400
H	−2.79512900	0.53595300	0.03285300
H	−6.33549300	−2.32870500	−0.02847200
H	−8.29965400	−0.83485800	0.21487100

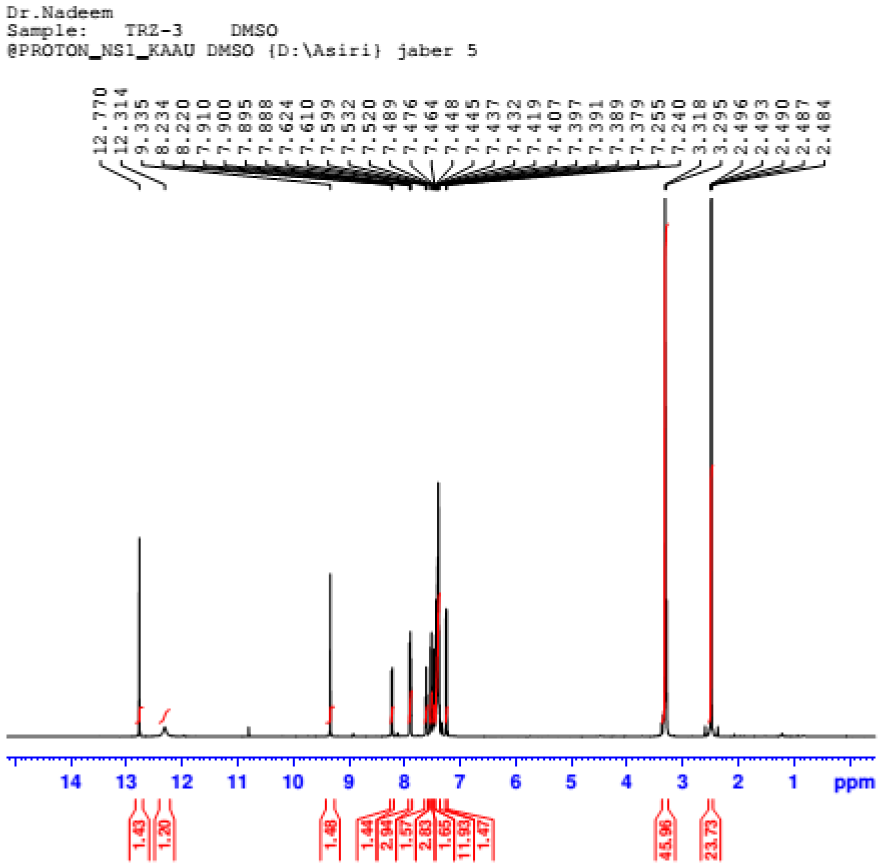

Figure S1. Experimental ^{1}H-NMR spectrum of **1** measured in *d*6-DMSO.

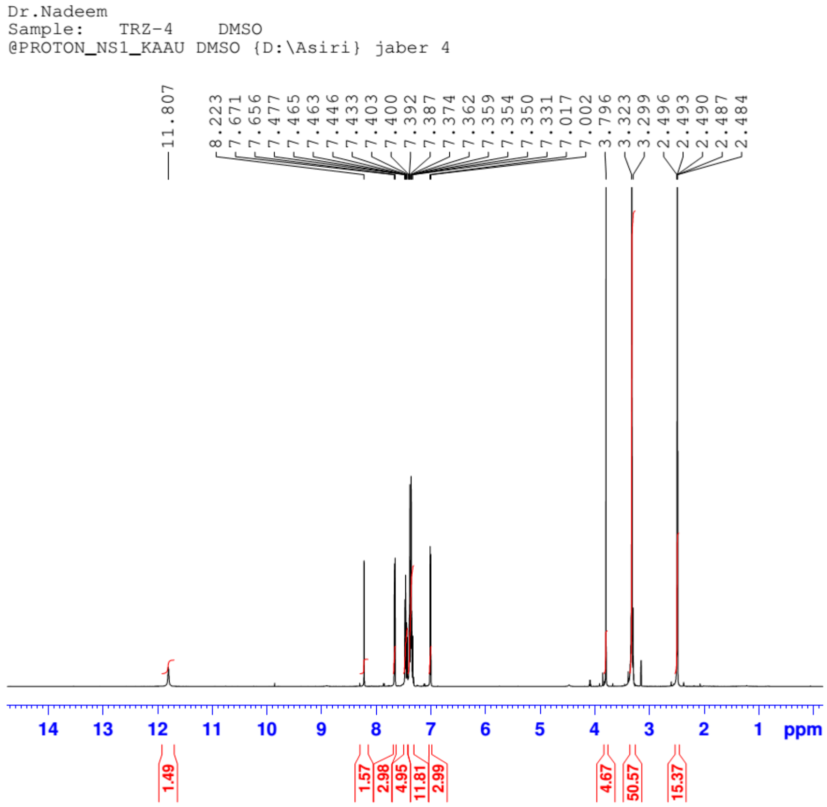

Figure S2. Experimental ^{1}H-NMR spectrum of **2** measured in *d*6-DMSO.

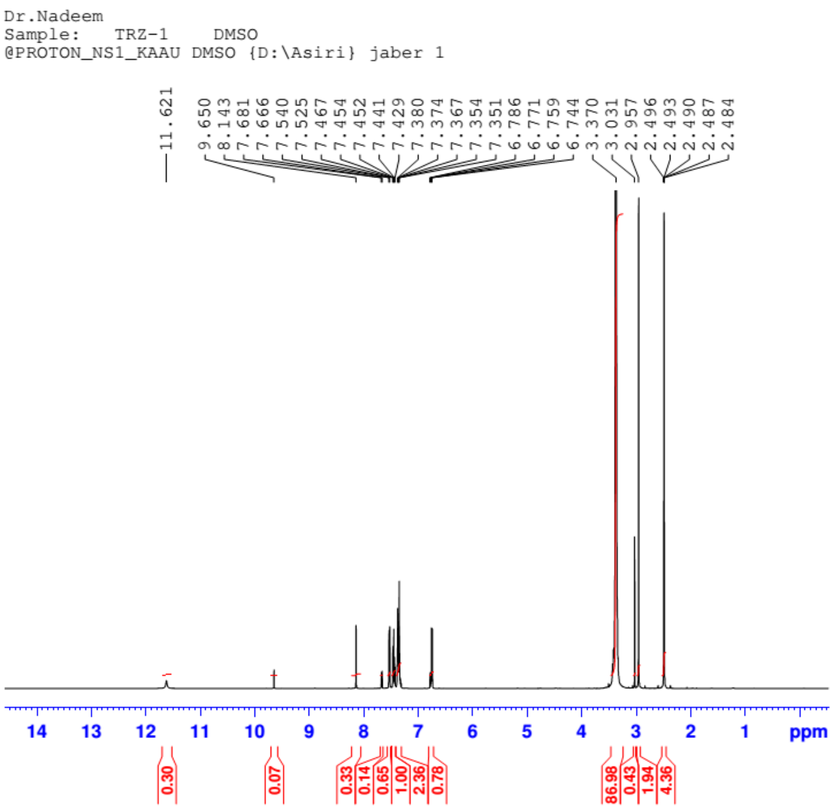

Figure S3. Experimental ^{1}H-NMR spectrum of **3** measured in d_6-DMSO.

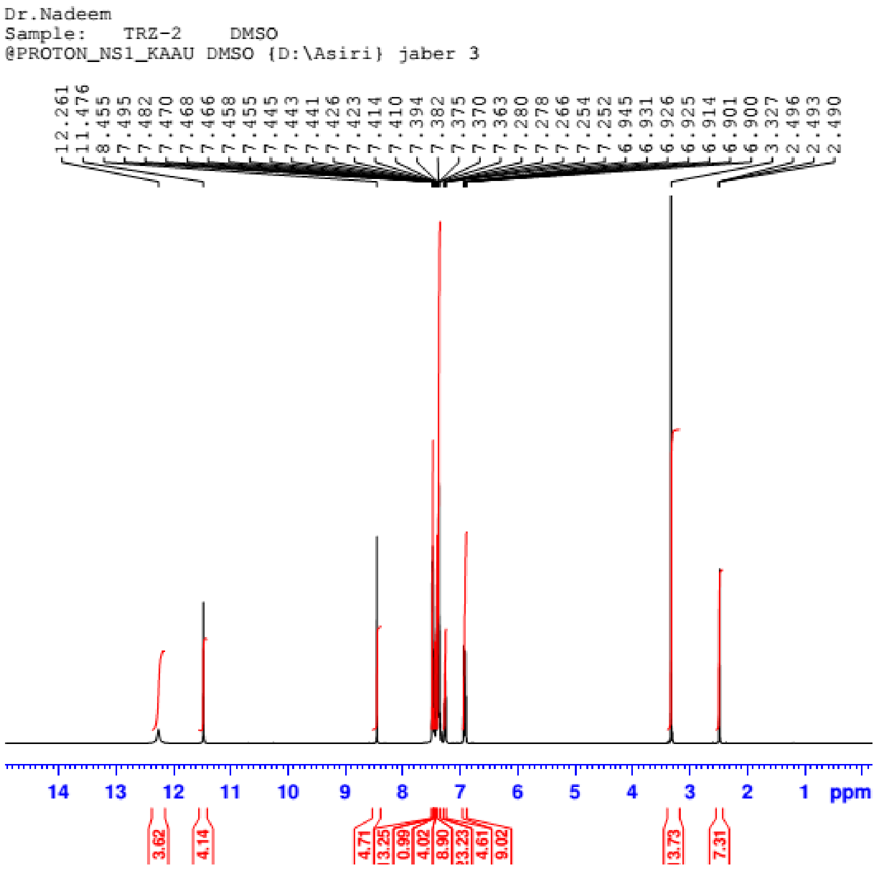

Figure S4. Experimental ^{1}H-NMR spectrum of **4** measured in d_6-DMSO.

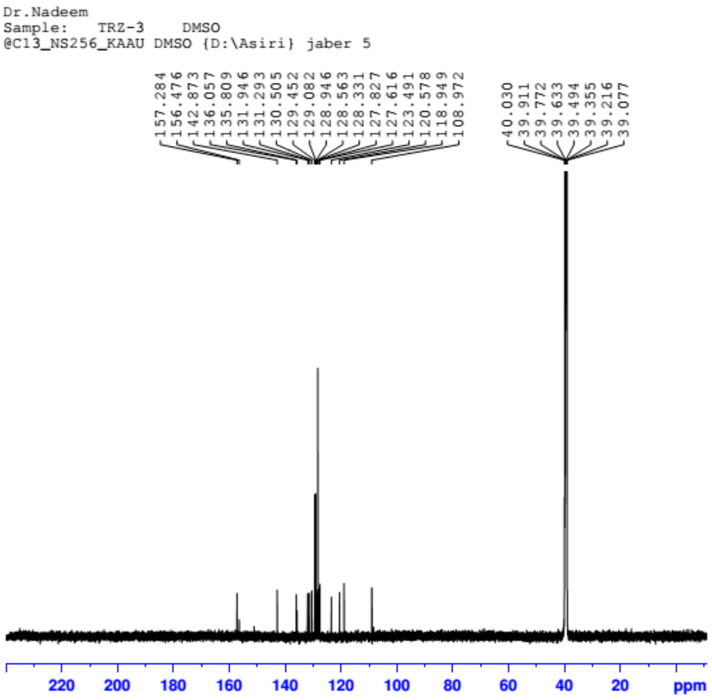

Figure S5. Experimental ^{13}C-NMR spectrum of **1** measured in d_6-DMSO.

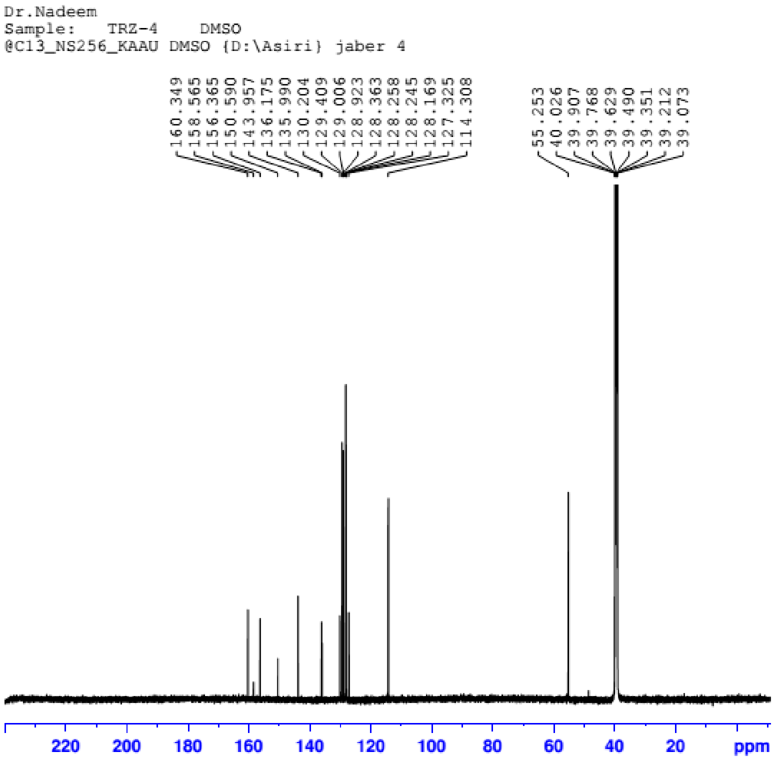

Figure S6. Experimental ^{13}C-NMR spectrum of **2** measured in d_6-DMSO.

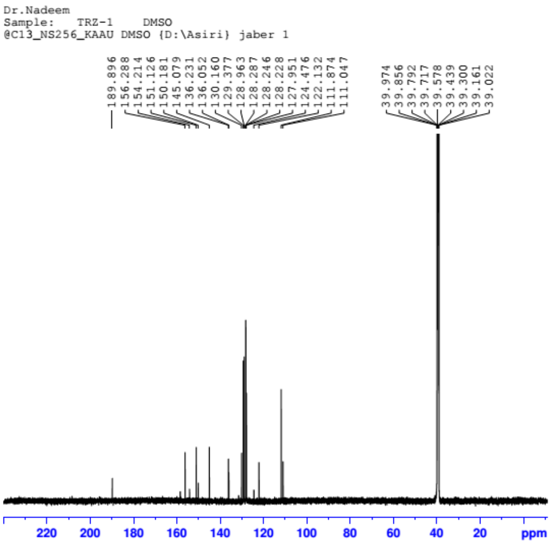

Figure S7. Experimental ^{13}C-NMR spectrum of **3** measured in d_6-DMSO.

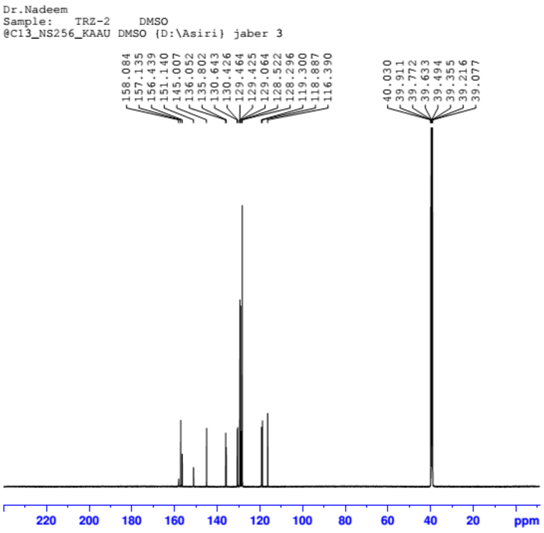

Figure S8. Experimental ^{13}C-NMR spectrum of **4** measured in d_6-DMSO.

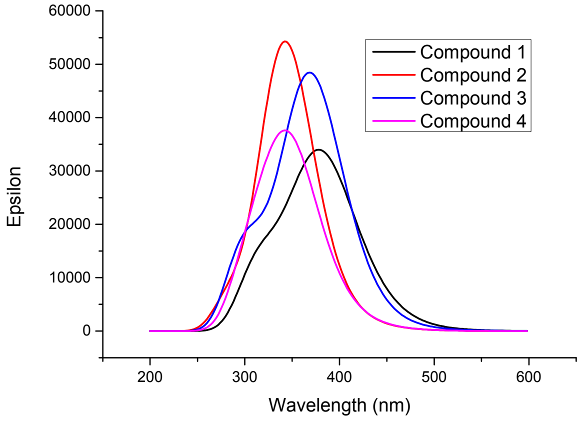

Figure S9. Combined simulated UV–vis. spectra of (**1**–**4**) measured at TD–B3LYP/6–31G (d, p) level in gas phase.

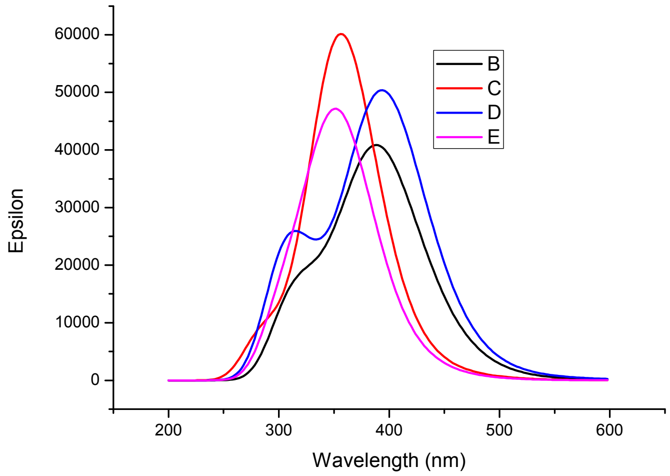

Figure S10. Combined simulated UV–vis. spectra of Compounds (**1**–**4**) measured at TD–B3LYP/6–31G (d, p) level measured in DMSO.

Molecule 1Cif

data_c:\nadeem

_audit_creation_method SHELXL−97
_chemical_name_systematic
;
1−[(5,6−Diphenyl−[1,2,4]triazin−3−yl)−hydrazonomethyl]−naphthalen−2−ol
;
_chemical_name_common
;
1−[(5,6−Diphenyl−[1,2,4]triazin−3−yl)−hydrazonomethyl]−naphthalen−2−ol
;
_chemical_melting_point ?
_chemical_formula_moiety 'C26H19N5 O'
_chemical_formula_sum
'C26H19N5 O'
_chemical_formula_weight 417.46

loop_
_atom_type_symbol
_atom_type_description
_atom_type_scat_dispersion_real
_atom_type_scat_dispersion_imag
_atom_type_scat_source
'C' 'C' 0.0181 0.0091
'International Tables VolC Tables 4.2.6.8 and 6.1.1.4'

```
'H' 'H' 0.0000 0.0000
'International Tables VolC Tables 4.2.6.8 and 6.1.1.4'
'N' 'N' 0.0311 0.0180
'International Tables VolC Tables 4.2.6.8 and 6.1.1.4'
'O' 'O' 0.0492 0.0322
'International Tables VolC Tables 4.2.6.8 and 6.1.1.4'

_symmetry_cell_setting            'Monoclinic'
_symmetry_space_group_name_H−M    'P2 1/n'
_symmetry_space_group_name_Hall   '−P2yn'

loop_
 _symmetry_equiv_pos_as_xyz
 'x, y, z'
 '−x+1/2, y+1/2, −z+1/2'
 '−x, −y, −z'
 'x−1/2, −y−1/2, z−1/2'

_cell_length_a                    6.5745(2)
_cell_length_b                    19.9133(6)
_cell_length_c                    15.9805(5)
_cell_angle_alpha                 90.00
_cell_angle_beta                  96.369(3)
_cell_angle_gamma                 90.00
_cell_volume                      2079.25(11)
_cell_formula_units_Z             4
_cell_measurement_temperature     296(2)
_cell_measurement_reflns_used     4636
_cell_measurement_theta_min       3.5567
_cell_measurement_theta_max       75.8131
_exptl_crystal_description        'rod'
_exptl_crystal_colour             'orange'
_exptl_crystal_size_max           0.48
_exptl_crystal_size_mid           0.16
_exptl_crystal_size_min           0.15
_exptl_crystal_density_meas       ?
_exptl_crystal_density_diffrn     1.334
_exptl_crystal_density_method     'not measured'
_exptl_crystal_F_000              872
_exptl_absorpt_coefficient_mu     0.677
_exptl_absorpt_correction_T_min   0.71210
_exptl_absorpt_correction_T_max   1.00000
```

```
_exptl_absorpt_correction_type          'multi−scan'
_exptl_absorpt_process_details
;
CrysAlisPro, Agilent Technologies,
Version 1.171.36.20 (release 27−06−2012CrysAlis171 .NET)
(compiled Jul 11 2012,15:38:31)
Empirical absorptionCorrection using sphericalHarmonics,
 implemented in SCALE3 ABSPACK scaling algorithm.
;

_exptl_special_details          ?

_diffrn_ambient_temperature       296(2)
_diffrn_radiation_wavelength      1.54184
_diffrn_radiation_type          CuK\a
_diffrn_radiation_source         'fine−focus sealed tube'
_diffrn_radiation_monochromator   graphite
_diffrn_measurement_device_type   'SuperNova, Dual,Cu at zero, Atlas,CCD'
_diffrn_measurement_method        '\w scans'
_diffrn_detector_area_resol_mean  ?
_diffrn_standards_number         0
_diffrn_standards_interval_count  .
_diffrn_standards_interval_time   .
_diffrn_standards_decay_%        ?
_diffrn_reflns_number           12339
_diffrn_reflns_av_R_equivalents   0.0185
_diffrn_reflns_av_sigmaI/netI     0.0160
_diffrn_reflns_limit_h_min       −6
_diffrn_reflns_limit_h_max       8
_diffrn_reflns_limit_k_min       −25
_diffrn_reflns_limit_k_max       21
_diffrn_reflns_limit_l_min       −20
_diffrn_reflns_limit_l_max       19
_diffrn_reflns_theta_min         3.56
_diffrn_reflns_theta_max         76.00
_reflns_number_total            4304
_reflns_number_gt              3378
_reflns_threshold_expression      >2sigma(I)

_computing_data_collection     'CrysAlis PRO (Agilent, 2012)'
_computing_cell_refinement      'CrysAlis PRO (Agilent, 2012)'
_computing_data_reduction     'CrysAlis PRO (Agilent, 2012)'
```

```
_computing_structure_solution     'SHELXS−97 (Sheldrick, 1990)'
_computing_structure_refinement   'SHELXL−97 (Sheldrick, 1997)'
_computing_molecular_graphics     ?
_computing_publication_material   ?

_iucr_refine_instructions_details
;
TITL 14040 in P2(1)/n
CELL 1.54184 6.5745 19.9133 15.9805 90.000 96.369 90.000
ZERR 4.0000 0.0002 0.0006 0.0005 0.000 0.003 0.000
LATT 1
SYMM 0.5−X, 0.5+Y, 0.5−Z
SFACCHN O
UNIT 104 76 20 4
MERG 2
FMAP 2
GRID
PLAN 20
TEMP 23
SIZE 0.48 0.16 0.15
BOND $H
HTAB
CONF
L.S. 15
ACTA 52
WGHT 0.06050 0.31320 0.00000 0.00000 0.00000 0.33330
FVAR 5.19820
N1    3  0.65264  0.03442  0.14503 11.00000  0.05845  0.05648 =
      0.04739 −0.00239  0.01050  0.00118
C2    1  0.68311  0.00284  0.21855 11.00000  0.05185  0.04894 =
      0.04712 −0.00394  0.00665 −0.00510
N2    3  0.94170 −0.02273  0.10197 11.00000  0.06634  0.06351 =
      0.05186  0.00111  0.01592  0.00687
N3    3  0.96464 −0.05728  0.17355 11.00000  0.06178  0.05772 =
      0.05537 −0.00058  0.01294  0.00388
N5    3  0.60803  0.09574 −0.00542 11.00000  0.06318  0.06522 =
      0.05439 −0.00340  0.01108  0.00839
N4    3  0.75933  0.04938  0.01218 11.00000  0.07255  0.07502 =
      0.05289  0.00850  0.01935  0.01977
C16   1  0.60562  0.12956 −0.07406 11.00000  0.05662  0.05982 =
      0.05149 −0.00593  0.00817  0.00423
AFIX 43
```

```
H16    2   0.70769   0.12283  −0.10909  11.00000  −1.20000
AFIX 0
C17    1   0.44474   0.17814  −0.09724  11.00000   0.05206   0.05525 =
       0.06199  −0.01641   0.00025   0.00187
O1     4   0.26256   0.14727   0.01996  11.00000   0.07524   0.09976 =
       0.08665  −0.01813   0.02980   0.00230
C3     1   0.83687  −0.04733   0.23186  11.00000   0.05265   0.05078 =
       0.04977  −0.00361   0.00787  −0.00290
C1     1   0.78237   0.01918   0.08875  11.00000   0.06045   0.05600 =
       0.04846  −0.00303   0.01089   0.00130
C10    1   0.87174  −0.09157   0.30699  11.00000   0.06036   0.04663 =
       0.05235  −0.00234   0.00782   0.00138
C4     1   0.55179   0.02649   0.28247  11.00000   0.06050   0.04692 =
       0.05020  −0.00025   0.01421  −0.00241
C22    1   0.45290   0.22129  −0.16932  11.00000   0.06078   0.04820 =
       0.06547  −0.01198  −0.01089   0.00379
C15    1   1.06929  −0.10474   0.34288  11.00000   0.06098   0.07059 =
       0.07676   0.01028   0.00792   0.00616
AFIX 43
H15    2   1.17982  −0.08493   0.32082  11.00000  −1.20000
AFIX 0
C11    1   0.71025  −0.12195   0.34051  11.00000   0.06394   0.05434 =
       0.05893  −0.00102   0.00811  −0.00285
AFIX 43
H11    2   0.57684  −0.11354   0.31706  11.00000  −1.20000
AFIX 0
C21    1   0.28750   0.26597  −0.19321  11.00000   0.06510   0.05507 =
       0.09240  −0.01992  −0.01384   0.00837
C9     1   0.62712   0.03353   0.36682  11.00000   0.08251   0.05689 =
       0.05299  −0.00217   0.00973   0.00318
AFIX 43
H9     2   0.76115   0.02132   0.38527  11.00000  −1.20000
AFIX 0
C23    1   0.61896   0.22284  −0.21813  11.00000   0.06597   0.05851 =
       0.06810  −0.00148  −0.00348   0.00258
AFIX 43
H23    2   0.73035   0.19471  −0.20388  11.00000  −1.20000
AFIX 0
C5     1   0.35235   0.04602   0.25593  11.00000   0.06013   0.06297 =
       0.06367   0.00198   0.01554  −0.00088
AFIX 43
H5     2   0.30247   0.04287   0.19929  11.00000  −1.20000
```

```
AFIX 0
C18   1  0.27932  0.18373 −0.05053  11.00000   0.05927   0.06991 =
     0.07449  −0.02360   0.00956  −0.00230
C19   1  0.11700  0.22897 −0.07540  11.00000   0.05551   0.08524 =
     0.11061  −0.03928   0.01213   0.00461
AFIX 43
H19   2  0.00602  0.23186 −0.04421  11.00000  −1.20000
AFIX 0
C12   1  0.74536 −0.16476  0.40862  11.00000   0.08891   0.05719 =
     0.06847   0.00651   0.02176  −0.00066
AFIX 43
H12   2  0.63565 −0.18523  0.43051  11.00000  −1.20000
AFIX 0
C24   1  0.62003  0.26500 −0.28631  11.00000   0.09234   0.06469 =
     0.07831   0.00716  −0.00299  −0.00408
AFIX 43
H24   2  0.73275  0.26534 −0.31670  11.00000  −1.20000
AFIX 0
C6    1  0.22776  0.07007  0.31319  11.00000   0.06948   0.07181 =
     0.09393   0.00400   0.03362   0.00546
AFIX 43
H6    2  0.09361  0.08245  0.29521  11.00000  −1.20000
AFIX 0
C26   1  0.29389  0.30726 −0.26564  11.00000   0.09873   0.05040 =
     0.10238  −0.00167  −0.03025   0.01510
AFIX 43
H26   2  0.18335  0.33513 −0.28242  11.00000  −1.20000
AFIX 0
C20   1  0.12352  0.26795 −0.14449  11.00000   0.06813   0.06830 =
     0.11506  −0.02553  −0.01397   0.01567
AFIX 43
H20   2  0.01525  0.29704 −0.16007  11.00000  −1.20000
AFIX 0
C14   1  1.10248 −0.14725  0.41141  11.00000   0.07616   0.08202 =
     0.08226   0.01401  −0.00047   0.01825
AFIX 43
H14   2  1.23541 −0.15558  0.43552  11.00000  −1.20000
AFIX 0
C7    1  0.30152  0.07574  0.39652  11.00000   0.10837   0.06968 =
     0.08312  −0.00576   0.04938   0.00348
AFIX 43
H7    2  0.21637  0.09105  0.43519  11.00000  −1.20000
```

```
AFIX 0
C8    1   0.50102   0.05887   0.42313  11.00000   0.12174   0.06718 =
      0.05460  −0.00545   0.02563   0.00032
AFIX 43
H8    2   0.55165   0.06451   0.47938  11.00000  −1.20000
AFIX 0
C13    1   0.94141  −0.17718   0.44405  11.00000   0.10531   0.06408 =
      0.06807   0.01568   0.01221   0.01992
AFIX 43
H13    2   0.96473  −0.20582   0.49008  11.00000  −1.20000
AFIX 0
C25    1   0.45545   0.30703  −0.31044  11.00000   0.11083   0.06160 =
      0.09242   0.00879  −0.01289   0.00479
AFIX 43
H25    2   0.45689   0.33479  −0.35717  11.00000  −1.20000
H2    2   0.86974   0.04137  −0.02193  11.00000   0.08658
H1    2   0.39354   0.11887   0.03188  11.00000   0.12375
HKLF 4
;
_refine_special_details
;
 Refinement of F^2^ against ALL reflections.  The weighted R−factor wR and
 goodness of fit S are based on F^2^,Conventional R−factors R are based
 on F, with F set to zero forNegative F^2^. The threshold expression of
 F^2^ > 2sigma(F^2^) is used only forCalculating R−factors(gt) etc. and is
Not relevant to theChoice of reflections for refinement.  R−factors based
 on F^2^ are statistically about twice as large as those based on F, and R−
 factors based on ALL data will be even larger.
;

_refine_ls_structure_factor_coef  Fsqd
_refine_ls_matrix_type            full
_refine_ls_weighting_scheme       Calc
_refine_ls_weighting_details
 'calc w=1/[\s^2^(Fo^2^)+(0.0605P)^2^+0.3132P] where P=(Fo^2^+2Fc^2^)/3'
_atom_sites_solution_primary      direct
_atom_sites_solution_secondary    difmap
_atom_sites_solution_hydrogens    geom
_refine_ls_hydrogen_treatment     Constr
_refine_ls_extinction_method      None
_refine_ls_extinction_coef        ?
_refine_ls_number_reflns          4304
```

_refine_ls_number_parameters 291
_refine_ls_number_restraints 0
_refine_ls_R_factor_all 0.0546
_refine_ls_R_factor_gt 0.0428
_refine_ls_wR_factor_ref 0.1273
_refine_ls_wR_factor_gt 0.1156
_refine_ls_goodness_of_fit_ref 1.034
_refine_ls_restrained_S_all 1.034
_refine_ls_shift/su_max 0.000
_refine_ls_shift/su_mean 0.000

loop_
_atom_site_label
_atom_site_type_symbol
_atom_site_fract_x
_atom_site_fract_y
_atom_site_fract_z
_atom_site_U_iso_or_equiv
_atom_site_adp_type
_atom_site_occupancy
_atom_site_symetry_multiplicity
_atom_site_calc_flag
_atom_site_refinement_flags
_atom_site_disorder_assembly
_atom_site_disorder_group
N1N 0.65264(18) 0.03442(6) 0.14503(7) 0.0538(3) Uani 1 1 d . . .
C2C 0.6831(2) 0.00284(7) 0.21855(8) 0.0492(3) Uani 1 1 d . . .
N2N 0.9417(2) −0.02273(7) 0.10197(8) 0.0599(3) Uani 1 1 d . . .
N3N 0.96465(19) −0.05728(6) 0.17355(8) 0.0578(3) Uani 1 1 d . . .
N5N 0.6080(2) 0.09574(7) −0.00542(8) 0.0606(3) Uani 1 1 d . . .
N4N 0.7593(2) 0.04938(7) 0.01218(8) 0.0659(4) Uani 1 1 d . . .
C16C 0.6056(2) 0.12956(8) −0.07406(9) 0.0558(3) Uani 1 1 d . . .
H16H 0.7077 0.1228 −0.1091 0.067 Uiso 1 1Calc R . .
C17C 0.4447(2) 0.17814(8) −0.09724(10) 0.0569(4) Uani 1 1 d . . .
O1 O 0.2626(2) 0.14727(8) 0.01996(9) 0.0857(4) Uani 1 1 d . . .
C3C 0.8369(2) −0.04733(7) 0.23186(9) 0.0509(3) Uani 1 1 d . . .
C1C 0.7824(2) 0.01918(8) 0.08875(9) 0.0546(3) Uani 1 1 d . . .
C10C 0.8717(2) −0.09157(7) 0.30699(9) 0.0530(3) Uani 1 1 d . . .
C4C 0.5518(2) 0.02649(7) 0.28247(9) 0.0519(3) Uani 1 1 d . . .
C22C 0.4529(2) 0.22129(7) −0.16932(10) 0.0595(4) Uani 1 1 d . . .
C15C 1.0693(3) −0.10474(9) 0.34288(11) 0.0694(4) Uani 1 1 d . . .
H15H 1.1798 −0.0849 0.3208 0.083 Uiso 1 1Calc R . .

C11C 0.7102(2) −0.12195(8) 0.34051(10) 0.0590(4) Uani 1 1 d . . .
H11H 0.5768 −0.1135 0.3171 0.071 Uiso 1 1Calc R . .
C21C 0.2875(3) 0.26597(8) −0.19321(12) 0.0725(5) Uani 1 1 d . . .
C9C 0.6271(3) 0.03353(8) 0.36682(10) 0.0640(4) Uani 1 1 d . . .
H9H 0.7612 0.0213 0.3853 0.077 Uiso 1 1Calc R . .
C23C 0.6190(3) 0.22284(8) −0.21812(10) 0.0650(4) Uani 1 1 d . . .
H23H 0.7303 0.1947 −0.2039 0.078 Uiso 1 1Calc R . .
C5C 0.3524(2) 0.04601(8) 0.25593(11) 0.0616(4) Uani 1 1 d . . .
H5H 0.3025 0.0429 0.1993 0.074 Uiso 1 1Calc R . .
C18C 0.2793(2) 0.18373(9) −0.05053(11) 0.0677(4) Uani 1 1 d . . .
C19C 0.1170(3) 0.22897(11) −0.07540(15) 0.0836(6) Uani 1 1 d . . .
H19H 0.0060 0.2319 −0.0442 0.100 Uiso 1 1Calc R . .
C12C 0.7454(3) −0.16476(9) 0.40862(11) 0.0705(4) Uani 1 1 d . . .
H12H 0.6356 −0.1852 0.4305 0.085 Uiso 1 1Calc R . .
C24C 0.6200(3) 0.26500(9) −0.28631(12) 0.0794(5) Uani 1 1 d . . .
H24H 0.7328 0.2653 −0.3167 0.095 Uiso 1 1Calc R . .
C6C 0.2278(3) 0.07007(9) 0.31319(13) 0.0766(5) Uani 1 1 d . . .
H6H 0.0936 0.0824 0.2952 0.092 Uiso 1 1Calc R . .
C26C 0.2939(4) 0.30726(9) −0.26564(14) 0.0869(6) Uani 1 1 d . . .
H26H 0.1834 0.3351 −0.2824 0.104 Uiso 1 1Calc R . .
C20C 0.1235(3) 0.26795(10) −0.14449(15) 0.0856(6) Uani 1 1 d . . .
H20H 0.0153 0.2970 −0.1601 0.103 Uiso 1 1Calc R . .
C14C 1.1025(3) −0.14725(10) 0.41141(12) 0.0808(5) Uani 1 1 d . . .
H14H 1.2354 −0.1556 0.4355 0.097 Uiso 1 1Calc R . .
C7C 0.3015(4) 0.07574(10) 0.39652(13) 0.0842(6) Uani 1 1 d . . .
H7H 0.2164 0.0911 0.4352 0.101 Uiso 1 1Calc R . .
C8C 0.5010(4) 0.05887(9) 0.42313(11) 0.0800(5) Uani 1 1 d . . .
H8H 0.5517 0.0645 0.4794 0.096 Uiso 1 1Calc R . .
C13C 0.9414(3) −0.17718(9) 0.44406(12) 0.0790(5) Uani 1 1 d . . .
H13H 0.9647 −0.2058 0.4901 0.095 Uiso 1 1Calc R . .
C25C 0.4555(4) 0.30703(10) −0.31044(14) 0.0901(6) Uani 1 1 d . . .
H25H 0.4569 0.3348 −0.3572 0.108 Uiso 1 1Calc R . .
H2H 0.8697 0.0414 −0.0219 0.087(6) Uiso 1 1 d R . .
H1H 0.3935 0.1189 0.0319 0.124(9) Uiso 1 1 d R . .

loop_
_atom_site_aniso_label
_atom_site_aniso_U_11
_atom_site_aniso_U_22
_atom_site_aniso_U_33
_atom_site_aniso_U_23
_atom_site_aniso_U_13

_atom_site_aniso_U_12
N1 0.0584(6) 0.0565(7) 0.0474(6) −0.0024(5) 0.0105(5) 0.0012(5)
C2 0.0519(7) 0.0489(7) 0.0471(7) −0.0039(6) 0.0066(5) −0.0051(6)
N2 0.0663(7) 0.0635(7) 0.0519(7) 0.0011(6) 0.0159(5) 0.0069(6)
N3 0.0618(7) 0.0577(7) 0.0554(7) −0.0006(6) 0.0129(5) 0.0039(6)
N5 0.0632(7) 0.0652(8) 0.0544(7) −0.0034(6) 0.0111(5) 0.0084(6)
N4 0.0725(8) 0.0750(9) 0.0529(7) 0.0085(6) 0.0193(6) 0.0198(7)
C16 0.0566(8) 0.0598(8) 0.0515(8) −0.0059(6) 0.0082(6) 0.0042(7)
C17 0.0521(7) 0.0552(8) 0.0620(8) −0.0164(7) 0.0002(6) 0.0019(6)
O1 0.0752(8) 0.0998(10) 0.0866(9) −0.0181(8) 0.0298(7) 0.0023(7)
C3 0.0526(7) 0.0508(7) 0.0498(7) −0.0036(6) 0.0079(6) −0.0029(6)
C1 0.0605(8) 0.0560(8) 0.0485(7) −0.0030(6) 0.0109(6) 0.0013(7)
C10 0.0604(8) 0.0466(7) 0.0523(8) −0.0023(6) 0.0078(6) 0.0014(6)
C4 0.0605(8) 0.0469(7) 0.0502(7) −0.0003(6) 0.0142(6) −0.0024(6)
C22 0.0608(8) 0.0482(7) 0.0655(9) −0.0120(7) −0.0109(7) 0.0038(6)
C15 0.0610(9) 0.0706(10) 0.0768(11) 0.0103(8) 0.0079(8) 0.0062(8)
C11 0.0639(8) 0.0543(8) 0.0589(8) −0.0010(7) 0.0081(7) −0.0029(7)
C21 0.0651(9) 0.0551(9) 0.0924(12) −0.0199(9) −0.0138(9) 0.0084(7)
C9 0.0825(10) 0.0569(8) 0.0530(8) −0.0022(7) 0.0097(7) 0.0032(8)
C23 0.0660(9) 0.0585(9) 0.0681(10) −0.0015(7) −0.0035(7) 0.0026(7)
C5 0.0601(8) 0.0630(9) 0.0637(9) 0.0020(7) 0.0155(7) −0.0009(7)
C18 0.0593(9) 0.0699(10) 0.0745(11) −0.0236(9) 0.0096(8) −0.0023(8)
C19 0.0555(9) 0.0852(13) 0.1106(15) −0.0393(12) 0.0121(9) 0.0046(9)
C12 0.0889(12) 0.0572(9) 0.0685(10) 0.0065(8) 0.0218(9) −0.0007(8)
C24 0.0923(13) 0.0647(10) 0.0783(12) 0.0072(9) −0.0030(9) −0.0041(9)
C6 0.0695(10) 0.0718(11) 0.0939(13) 0.0040(10) 0.0336(9) 0.0055(9)
C26 0.0987(14) 0.0504(9) 0.1024(15) −0.0017(10) −0.0303(12) 0.0151(10)
C20 0.0681(11) 0.0683(11) 0.1151(16) −0.0255(11) −0.0140(11) 0.0157(9)
C14 0.0762(11) 0.0820(12) 0.0823(12) 0.0140(10) −0.0005(9) 0.0182(10)
C7 0.1084(15) 0.0697(11) 0.0831(13) −0.0058(10) 0.0494(12) 0.0035(11)
C8 0.1217(17) 0.0672(10) 0.0546(9) −0.0054(8) 0.0256(10) 0.0003(11)
C13 0.1053(14) 0.0641(10) 0.0681(10) 0.0157(8) 0.0122(10) 0.0199(10)
C25 0.1108(17) 0.0616(11) 0.0924(14) 0.0088(10) −0.0129(13) 0.0048(11)

_geom_special_details
;
All esds (except the esd in the dihedral angle between two l.s. planes)
are estimated using the fullCovariance matrix. TheCell esds are taken
into account individually in the estimation of esds in distances, angles
and torsion angles;Correlations between esds inCell parameters are only
used when they are defined byCrystal symmetry. An approximate (isotropic)
treatment ofCell esds is used for estimating esds involving l.s. planes.

;

loop_
 _geom_bond_atom_site_label_1
 _geom_bond_atom_site_label_2
 _geom_bond_distance
 _geom_bond_site_symmetry_2
 _geom_bond_publ_flag
N1C2 1.3280(18) . ?
N1C1 1.3412(18) . ?
C2C3 1.4204(19) . ?
C2C4 1.4854(19) . ?
N2N3 1.3289(17) . ?
N2C1 1.3378(19) . ?
N3C3 1.3367(17) . ?
N5C16 1.2858(19) . ?
N5N4 1.3633(18) . ?
N4C1 1.3568(19) . ?
N4H2 0.9681 . ?
C16C17 1.451(2) . ?
C16H16 0.9300 . ?
C17C18 1.390(2) . ?
C17C22 1.443(2) . ?
O1C18 1.355(2) . ?
O1H1 1.0301 . ?
C3C10 1.486(2) . ?
C10C11 1.381(2) . ?
C10C15 1.385(2) . ?
C4C5 1.388(2) . ?
C4C9 1.391(2) . ?
C22C23 1.411(2) . ?
C22C21 1.424(2) . ?
C15C14 1.382(2) . ?
C15H15 0.9300 . ?
C11C12 1.382(2) . ?
C11H11 0.9300 . ?
C21C20 1.399(3) . ?
C21C26 1.424(3) . ?
C9C8 1.385(2) . ?
C9H9 0.9300 . ?
C23C24 1.376(2) . ?
C23H23 0.9300 . ?

C5C6 1.380(2) . ?
C5H5 0.9300 . ?
C18C19 1.419(3) . ?
C19C20 1.354(3) . ?
C19H19 0.9300 . ?
C12C13 1.372(3) . ?
C12H12 0.9300 . ?
C24C25 1.388(3) . ?
C24H24 0.9300 . ?
C6C7 1.370(3) . ?
C6H6 0.9300 . ?
C26C25 1.345(3) . ?
C26H26 0.9300 . ?
C20H20 0.9300 . ?
C14C13 1.368(3) . ?
C14H14 0.9300 . ?
C7C8 1.375(3) . ?
C7H7 0.9300 . ?
C8H8 0.9300 . ?
C13H13 0.9300 . ?
C25H25 0.9300 . ?

loop_
 _geom_angle_atom_site_label_1
 _geom_angle_atom_site_label_2
 _geom_angle_atom_site_label_3
 _geom_angle
 _geom_angle_site_symmetry_1
 _geom_angle_site_symmetry_3
 _geom_angle_publ_flag
C2N1C1 116.19(12) . . ?
N1C2C3 120.04(12) . . ?
N1C2C4 114.59(12) . . ?
C3C2C4 125.33(12) . . ?
N3N2C1 118.10(12) . . ?
N2N3C3 120.21(12) . . ?
C16N5N4 118.05(13) . . ?
C1N4N5 119.47(12) . . ?
C1N4H2 114.8 . . ?
N5N4H2 124.8 . . ?
N5C16C17 120.44(14) . . ?
N5C16H16 119.8 . . ?

C17C16H16 119.8 . . ?
C18C17C22 118.78(15) . . ?
C18C17C16 120.53(16) . . ?
C22C17C16 120.68(14) . . ?
C18 O1H1 107.7 . . ?
N3C3C2 119.45(13) . . ?
N3C3C10 115.01(13) . . ?
C2C3C10 125.54(12) . . ?
N2C1N1 125.47(13) . . ?
N2C1N4 115.22(13) . . ?
N1C1N4 119.27(13) . . ?
C11C10C15 118.84(14) . . ?
C11C10C3 121.11(13) . . ?
C15C10C3 120.01(14) . . ?
C5C4C9 119.38(14) . . ?
C5C4C2 118.69(13) . . ?
C9C4C2 121.80(14) . . ?
C23C22C21 116.93(16) . . ?
C23C22C17 123.64(14) . . ?
C21C22C17 119.42(16) . . ?
C14C15C10 120.16(16) . . ?
C14C15H15 119.9 . . ?
C10C15H15 119.9 . . ?
C10C11C12 120.50(16) . . ?
C10C11H11 119.8 . . ?
C12C11H11 119.8 . . ?
C20C21C22 118.77(19) . . ?
C20C21C26 122.02(18) . . ?
C22C21C26 119.22(18) . . ?
C8C9C4 119.49(17) . . ?
C8C9H9 120.3 . . ?
C4C9H9 120.3 . . ?
C24C23C22 121.56(16) . . ?
C24C23H23 119.2 . . ?
C22C23H23 119.2 . . ?
C6C5C4 120.32(16) . . ?
C6C5H5 119.8 . . ?
C4C5H5 119.8 . . ?
O1C18C17 122.96(16) . . ?
O1C18C19 116.43(16) . . ?
C17C18C19 120.62(18) . . ?
C20C19C18 120.09(18) . . ?

C20C19H19 120.0 . . ?
C18C19H19 120.0 . . ?
C13C12C11 120.25(17) . . ?
C13C12H12 119.9 . . ?
C11C12H12 119.9 . . ?
C23C24C25 121.0(2) . . ?
C23C24H24 119.5 . . ?
C25C24H24 119.5 . . ?
C7C6C5 120.06(18) . . ?
C7C6H6 120.0 . . ?
C5C6H6 120.0 . . ?
C25C26C21 121.79(18) . . ?
C25C26H26 119.1 . . ?
C21C26H26 119.1 . . ?
C19C20C21 122.26(17) . . ?
C19C20H20 118.9 . . ?
C21C20H20 118.9 . . ?
C13C14C15 120.53(18) . . ?
C13C14H14 119.7 . . ?
C15C14H14 119.7 . . ?
C6C7C8 120.20(16) . . ?
C6C7H7 119.9 . . ?
C8C7H7 119.9 . . ?
C7C8C9 120.49(18) . . ?
C7C8H8 119.8 . . ?
C9C8H8 119.8 . . ?
C14C13C12 119.72(16) . . ?
C14C13H13 120.1 . . ?
C12C13H13 120.1 . . ?
C26C25C24 119.4(2) . . ?
C26C25H25 120.3 . . ?
C24C25H25 120.3 . . ?

loop_
 _geom_torsion_atom_site_label_1
 _geom_torsion_atom_site_label_2
 _geom_torsion_atom_site_label_3
 _geom_torsion_atom_site_label_4
 _geom_torsion
 _geom_torsion_site_symmetry_1
 _geom_torsion_site_symmetry_2
 _geom_torsion_site_symmetry_3

_geom_torsion_site_symmetry_4
_geom_torsion_publ_flag
C1N1C2C3 −4.29(19) . . . . ?
C1N1C2C4 173.48(12) . . . . ?
C1N2N3C3 −3.3(2) . . . . ?
C16N5N4C1 171.46(14) . . . . ?
N4N5C16C17 178.17(13) . . . . ?
N5C16C17C18 −6.9(2) . . . . ?
N5C16C17C22 173.35(13) . . . . ?
N2N3C3C2 −3.2(2) . . . . ?
N2N3C3C10 176.88(12) . . . . ?
N1C2C3N3 7.2(2) . . . . ?
C4C2C3N3 −170.28(13) . . . . ?
N1C2C3C10 −172.86(13) . . . . ?
C4C2C3C10 9.6(2) . . . . ?
N3N2C1N1 6.6(2) . . . . ?
N3N2C1N4 −175.59(13) . . . . ?
C2N1C1N2 −2.6(2) . . . . ?
C2N1C1N4 179.68(13) . . . . ?
N5N4C1N2 −176.42(14) . . . . ?
N5N4C1N1 1.5(2) . . . . ?
N3C3C10C11 −135.86(15) . . . . ?
C2C3C10C11 44.2(2) . . . . ?
N3C3C10C15 41.9(2) . . . . ?
C2C3C10C15 −137.96(16) . . . . ?
N1C2C4C5 35.75(19) . . . . ?
C3C2C4C5 −146.61(14) . . . . ?
N1C2C4C9 −140.12(14) . . . . ?
C3C2C4C9 37.5(2) . . . . ?
C18C17C22C23 176.02(14) . . . . ?
C16C17C22C23 −4.3(2) . . . . ?
C18C17C22C21 −3.1(2) . . . . ?
C16C17C22C21 176.66(13) . . . . ?
C11C10C15C14 −0.4(3) . . . . ?
C3C10C15C14 −178.31(16) . . . . ?
C15C10C11C12 0.0(2) . . . . ?
C3C10C11C12 177.79(14) . . . . ?
C23C22C21C20 −177.48(15) . . . . ?
C17C22C21C20 1.7(2) . . . . ?
C23C22C21C26 2.4(2) . . . . ?
C17C22C21C26 −178.44(14) . . . . ?
C5C4C9C8 1.0(2) . . . . ?

C2C4C9C8 176.88(15) . . . . ?
C21C22C23C24 −0.8(2) . . . . ?
C17C22C23C24 −179.87(15) . . . . ?
C9C4C5C6 −2.2(2) . . . . ?
C2C4C5C6 −178.14(14) . . . . ?
C22C17C18 O1 −177.32(14) . . . . ?
C16C17C18 O1 3.0(2) . . . . ?
C22C17C18C19 2.7(2) . . . . ?
C16C17C18C19 −177.00(15) . . . . ?
O1C18C19C20 179.07(16) . . . . ?
C17C18C19C20 −1.0(3) . . . . ?
C10C11C12C13 0.4(3) . . . . ?
C22C23C24C25 −1.0(3) . . . . ?
C4C5C6C7 1.0(3) . . . . ?
C20C21C26C25 177.43(18) . . . . ?
C22C21C26C25 −2.5(3) . . . . ?
C18C19C20C21 −0.5(3) . . . . ?
C22C21C20C19 0.1(3) . . . . ?
C26C21C20C19 −179.77(17) . . . . ?
C10C15C14C13 0.6(3) . . . . ?
C5C6C7C8 1.4(3) . . . . ?
C6C7C8C9 −2.5(3) . . . . ?
C4C9C8C7 1.3(3) . . . . ?
C15C14C13C12 −0.2(3) . . . . ?
C11C12C13C14 −0.3(3) . . . . ?
C21C26C25C24 0.7(3) . . . . ?
C23C24C25C26 1.0(3) . . . . ?

_diffrn_measured_fraction_theta_max 0.992
_diffrn_reflns_theta_full 66.50
_diffrn_measured_fraction_theta_full 0.998
_refine_diff_density_max 0.188
_refine_diff_density_min −0.206
_refine_diff_density_rms 0.033

Molecule 2Cif

data_c:\nadeem

_audit_creation_method SHELXL−97
_chemical_name_systematic
;
<i>N<i/>−(5,6−Diphenyl−[1,2,4]triazin−3−yl)−<i>N<i/>'−(4−methoxy−benzylidene)−

hydrazine
;
_chemical_name_common
;
<i>N<i/>−(5,6−Diphenyl−[1,2,4]triazin−3−yl)−<i>N<i/>'−(4−methoxy−benzylidene)−
hydrazine
;
_chemical_melting_point ?
_chemical_formula_moiety 'C23H19N5 O'
_chemical_formula_sum
'C23H19N5 O'
_chemical_formula_weight 381.43

loop_
_atom_type_symbol
_atom_type_description
_atom_type_scat_dispersion_real
_atom_type_scat_dispersion_imag
_atom_type_scat_source
'C' 'C' 0.0181 0.0091
'International Tables VolC Tables 4.2.6.8 and 6.1.1.4'
'H' 'H' 0.0000 0.0000
'International Tables VolC Tables 4.2.6.8 and 6.1.1.4'
'N' 'N' 0.0311 0.0180
'International Tables VolC Tables 4.2.6.8 and 6.1.1.4'
'O' 'O' 0.0492 0.0322
'International Tables VolC Tables 4.2.6.8 and 6.1.1.4'

_symmetry_cell_setting 'Monoclinic'
_symmetry_space_group_name_H−M 'P21/c'
_symmetry_space_group_name_Hall '−P2ybc'

loop_
_symmetry_equiv_pos_as_xyz
'x, y, z'
'−x, y+1/2, −z+1/2'
'−x, −y, −z'
'x, −y−1/2, z−1/2'

_cell_length_a 5.9756(18)
_cell_length_b 21.573(6)
_cell_length_c 14.980(4)

_cell_angle_alpha 90.00
_cell_angle_beta 93.45(2)
_cell_angle_gamma 90.00
_cell_volume 1927.7(9)
_cell_formula_units_Z 4
_cell_measurement_temperature 296(2)
_cell_measurement_reflns_used 1176
_cell_measurement_theta_min 2.8334
_cell_measurement_theta_max 29.2995

_exptl_crystal_description 'needle'
_exptl_crystal_colour 'Yellow'
_exptl_crystal_size_max 0.48
_exptl_crystal_size_mid 0.08
_exptl_crystal_size_min 0.05
_exptl_crystal_density_meas ?
_exptl_crystal_density_diffrn 1.314
_exptl_crystal_density_method 'not measured'
_exptl_crystal_F_000 800
_exptl_absorpt_coefficient_mu 0.084
_exptl_absorpt_correction_T_min 0.71210
_exptl_absorpt_correction_T_max 1.00000
_exptl_absorpt_correction_type 'multi−scan'
_exptl_absorpt_process_details
;
CrysAlisPro, Agilent Technologies,
Version 1.171.36.20 (release 27−06−2012CrysAlis171 .NET)
(compiled Jul 11 2012,15:38:31)
Empirical absorptionCorrection using sphericalHarmonics,
implemented in SCALE3 ABSPACK scaling algorithm.
;

_exptl_special_details ?

_diffrn_ambient_temperature 296(2)
_diffrn_radiation_wavelength 0.71073
_diffrn_radiation_type MoK\a
_diffrn_radiation_source 'fine−focus sealed tube'
_diffrn_radiation_monochromator graphite
_diffrn_measurement_device_type 'SuperNova, Dual,Cu at zero, Atlas,CCD'
_diffrn_measurement_method '\w scans'
_diffrn_detector_area_resol_mean ?

```
_diffrn_standards_number          0
_diffrn_standards_interval_count  .
_diffrn_standards_interval_time   .
_diffrn_standards_decay_%         ?
_diffrn_reflns_number             11554
_diffrn_reflns_av_R_equivalents   0.0727
_diffrn_reflns_av_sigmaI/netI     0.1262
_diffrn_reflns_limit_h_min        −7
_diffrn_reflns_limit_h_max        8
_diffrn_reflns_limit_k_min        −27
_diffrn_reflns_limit_k_max        24
_diffrn_reflns_limit_l_min        −20
_diffrn_reflns_limit_l_max        18
_diffrn_reflns_theta_min          2.88
_diffrn_reflns_theta_max          29.33
_reflns_number_total              4608
_reflns_number_gt                 1775
_reflns_threshold_expression      >2sigma(I)

_computing_data_collection     'CrysAlis PRO (Agilent, 2012)'
_computing_cell_refinement     'CrysAlis PRO (Agilent, 2012)'
_computing_data_reduction      'CrysAlis PRO (Agilent, 2012)'
_computing_structure_solution    'SHELXS−97 (Sheldrick, 1990)'
_computing_structure_refinement  'SHELXL−97 (Sheldrick, 1997)'
_computing_molecular_graphics    ?
_computing_publication_material  ?

_iucr_refine_instructions_details
;
TITL 14097 in P2(1)/c
CELL 0.71073 5.9756 21.5731 14.9805 90.000 93.447 90.000
ZERR 4.0000 0.0018 0.0056 0.0040 0.000 0.022 0.000
LATT 1
SYMM −X, 0.5+Y, 0.5−Z
SFACCHN O
UNIT 92 76 20 4
MERG 2
FMAP 2
GRID
PLAN 20
TEMP 23
SIZE 0.05 0.08 0.48
```

```
BOND $H
HTAB
CONF
L.S. 20
ACTA 52
WGHT 0.02180 0.00000 0.00000 0.00000 0.00000 0.33330
FVAR 1.69020
O1    4   1.60874   0.41310   0.94281  11.00000   0.07147   0.08726 =
      0.08046   0.00578  −0.02662  −0.00019
N1    3   0.40402   0.35009   0.58890  11.00000   0.04992   0.04967 =
      0.05303   0.00216  −0.00043  −0.00648
N2    3   0.30440   0.43955   0.50047  11.00000   0.05052   0.04896 =
      0.06466   0.00143  −0.00525  −0.00137
N3    3   0.12818   0.40936   0.46126  11.00000   0.04805   0.05515 =
      0.05971   0.00165  −0.00407   0.00092
N4    3   0.62325   0.44122   0.58888  11.00000   0.05869   0.05261 =
      0.06208   0.00461  −0.01612  −0.00987
AFIX 43
H4    2   0.64324   0.47722   0.56601  11.00000  −1.20000
AFIX 0
N5    3   0.77881   0.41939   0.65359  11.00000   0.05624   0.05530 =
      0.05242  −0.00253  −0.00885   0.00159
C1    1   0.43856   0.40747   0.56000  11.00000   0.04820   0.05064 =
      0.04908  −0.00425   0.00275  −0.00286
C2    1   0.22268   0.32186   0.55276  11.00000   0.04271   0.04990 =
      0.04181  −0.00298   0.00426  −0.00114
C3    1   0.08431   0.35113   0.48454  11.00000   0.04319   0.04251 =
      0.04982  −0.00051   0.00565   0.00340
C4    1   0.17969   0.25920   0.58764  11.00000   0.04459   0.04857 =
      0.04099   0.00430  −0.00508  −0.00432
C5    1   0.33970   0.21325   0.58139  11.00000   0.04747   0.05908 =
      0.05816   0.00481  −0.00035  −0.00207
AFIX 43
H5    2   0.47327   0.22209   0.55521  11.00000  −1.20000
AFIX 0
C6    1   0.30303   0.15481   0.61346  11.00000   0.06740   0.05151 =
      0.07096   0.00513  −0.00564   0.00425
AFIX 43
H6    2   0.40982   0.12399   0.60725  11.00000  −1.20000
AFIX 0
C7    1   0.10916   0.14150   0.65477  11.00000   0.08041   0.05691 =
      0.06422   0.01512  −0.00507  −0.01296
```

```
AFIX 43
H7    2  0.08485   0.10188   0.67668  11.00000  −1.20000
AFIX 0
C8    1  −0.04866   0.18733   0.66341  11.00000   0.06669   0.07522 =
     0.06261   0.01322   0.00984  −0.01721
AFIX 43
H8    2  −0.17815   0.17881   0.69264  11.00000  −1.20000
AFIX 0
C9    1  −0.01625   0.24557   0.62917  11.00000   0.05326   0.06124 =
     0.06158   0.00459   0.00509  −0.00351
AFIX 43
H9    2  −0.12560   0.27588   0.63380  11.00000  −1.20000
AFIX 0
C10   1  −0.10908   0.32283   0.43189  11.00000   0.04671   0.05251 =
     0.04420   0.00027   0.00045   0.00023
C11   1  −0.12049   0.26099   0.40957  11.00000   0.05002   0.06235 =
     0.05891   0.00293  −0.00613  −0.00107
AFIX 43
H11   2  −0.00533   0.23465   0.43001  11.00000  −1.20000
AFIX 0
C12   1  −0.29937   0.23697   0.35738  11.00000   0.06126   0.06120 =
     0.07264  −0.00545  −0.00166  −0.00994
AFIX 43
H12   2  −0.30288   0.19512   0.34249  11.00000  −1.20000
AFIX 0
C13   1  −0.47277   0.27576   0.32758  11.00000   0.05370   0.08541 =
     0.05614   0.00288  −0.00703  −0.01153
AFIX 43
H13   2  −0.59508   0.25983   0.29373  11.00000  −1.20000
AFIX 0
C14   1  −0.46381   0.33783   0.34811  11.00000   0.05198   0.07977 =
     0.05177   0.00622  −0.00326   0.00377
AFIX 43
H14   2  −0.57878   0.36412   0.32732  11.00000  −1.20000
AFIX 0
C15   1  −0.28347   0.36109   0.39974  11.00000   0.05158   0.06109 =
     0.04589   0.00039   0.00136   0.00749
AFIX 43
H15   2  −0.27852   0.40316   0.41328  11.00000  −1.20000
AFIX 0
C16   1   0.93780   0.45812   0.67206  11.00000   0.05638   0.05521 =
     0.05547  −0.00234  −0.00732  −0.00541
```

```
AFIX 43
H16   2   0.94002   0.49535   0.64084  11.00000  -1.20000
AFIX 0
C17   1   1.11498   0.44529   0.74065  11.00000   0.05349   0.04754 =
      0.04661  -0.00213  -0.00032  -0.00167
C18   1   1.12508   0.39093   0.79028  11.00000   0.05679   0.05639 =
      0.06396  -0.00294  -0.00697  -0.00507
AFIX 43
H18   2   1.01849   0.36013   0.77846  11.00000  -1.20000
AFIX 0
C19   1   1.29145   0.38206   0.85696  11.00000   0.06806   0.05696 =
      0.06783   0.00846  -0.00691  -0.00011
AFIX 43
H19   2   1.29513   0.34567   0.89031  11.00000  -1.20000
AFIX 0
C20   1   1.45299   0.42720   0.87435  11.00000   0.05667   0.06206 =
      0.05572  -0.00543  -0.01090   0.00618
C21   1   1.44882   0.48071   0.82603  11.00000   0.06194   0.05952 =
      0.06587  -0.00923  -0.01070  -0.00939
AFIX 43
H21   2   1.55791   0.51094   0.83713  11.00000  -1.20000
AFIX 0
C22   1   1.27842   0.48933   0.75977  11.00000   0.06931   0.04814 =
      0.06232   0.00361  -0.00772  -0.00766
AFIX 43
H22   2   1.27448   0.52606   0.72722  11.00000  -1.20000
AFIX 0
C23   1   1.78566   0.45639   0.96182  11.00000   0.06311   0.10751 =
      0.08569  -0.02149  -0.01883  -0.00220
AFIX 137
H23A   2   1.87234   0.46079   0.91027  11.00000  -1.50000
H23B   2   1.88034   0.44169   1.01138  11.00000  -1.50000
H23C   2   1.72336   0.49584   0.97659  11.00000  -1.50000
HKLF 4
;
_refine_special_details
;
 Refinement of F^2^ against ALL reflections.  The weighted R-factor wR and
 goodness of fit S are based on F^2^,Conventional R-factors R are based
 on F, with F set to zero forNegative F^2^. The threshold expression of
 F^2^ > 2sigma(F^2^) is used only forCalculating R-factors(gt) etc. and is
Not relevant to theChoice of reflections for refinement.  R-factors based
```

on F^2^ are statistically about twice as large as those based on F, and R−
factors based on ALL data will be even larger.
;

```
_refine_ls_structure_factor_coef  Fsqd
_refine_ls_matrix_type           full
_refine_ls_weighting_scheme      Calc
_refine_ls_weighting_details
 'calc w=1/[\s^2^(Fo^2^)+(0.0239P)^2^+0.0000P] where P=(Fo^2^+2Fc^2^)/3'
_atom_sites_solution_primary      direct
_atom_sites_solution_secondary    difmap
_atom_sites_solution_hydrogens    geom
_refine_ls_hydrogen_treatment     mixed
_refine_ls_extinction_method      None
_refine_ls_extinction_coef        ?
_refine_ls_number_reflns          4608
_refine_ls_number_parameters      267
_refine_ls_number_restraints      1
_refine_ls_R_factor_all           0.1988
_refine_ls_R_factor_gt            0.0627
_refine_ls_wR_factor_ref          0.1409
_refine_ls_wR_factor_gt           0.0956
_refine_ls_goodness_of_fit_ref    1.008
_refine_ls_restrained_S_all       1.008
_refine_ls_shift/su_max           0.000
_refine_ls_shift/su_mean          0.000

loop_
 _atom_site_label
 _atom_site_type_symbol
 _atom_site_fract_x
 _atom_site_fract_y
 _atom_site_fract_z
 _atom_site_U_iso_or_equiv
 _atom_site_adp_type
 _atom_site_occupancy
 _atom_site_symetry_multiplicity
 _atom_site_calc_flag
 _atom_site_refinement_flags
 _atom_site_disorder_assembly
 _atom_site_disorder_group
O1 O 1.6087(3) 0.41311(11) 0.94280(13) 0.0811(7) Uani 1 1 d . . .
```

N1N 0.4040(4) 0.35010(11) 0.58890(14) 0.0511(6) Uani 1 1 d . . .
N2N 0.3044(4) 0.43953(11) 0.50049(15) 0.0552(7) Uani 1 1 d . . .
N3N 0.1282(4) 0.40935(12) 0.46127(14) 0.0547(7) Uani 1 1 d . . .
N4N 0.6232(4) 0.44132(13) 0.58885(16) 0.0583(7) Uani 1 1 d D . .
N5N 0.7788(4) 0.41939(11) 0.65357(14) 0.0552(7) Uani 1 1 d . . .
C1C 0.4387(4) 0.40749(14) 0.56001(17) 0.0494(8) Uani 1 1 d . . .
C2C 0.2227(4) 0.32186(13) 0.55276(16) 0.0448(7) Uani 1 1 d . . .
C3C 0.0843(4) 0.35112(13) 0.48455(17) 0.0452(7) Uani 1 1 d . . .
C4C 0.1797(4) 0.25920(13) 0.58764(16) 0.0451(7) Uani 1 1 d . . .
C5C 0.3397(5) 0.21324(15) 0.58139(18) 0.0551(8) Uani 1 1 d . . .
H5H 0.4733 0.2221 0.5552 0.066 Uiso 1 1Calc R . .
C6C 0.3030(5) 0.15481(15) 0.6135(2) 0.0638(9) Uani 1 1 d . . .
H6H 0.4098 0.1240 0.6073 0.077 Uiso 1 1Calc R . .
C7C 0.1092(6) 0.14150(16) 0.6548(2) 0.0676(10) Uani 1 1 d . . .
H7H 0.0849 0.1019 0.6767 0.081 Uiso 1 1Calc R . .
C8C −0.0486(5) 0.18732(17) 0.66341(19) 0.0680(9) Uani 1 1 d . . .
H8H −0.1781 0.1788 0.6926 0.082 Uiso 1 1Calc R . .
C9C −0.0163(5) 0.24557(15) 0.62916(18) 0.0588(8) Uani 1 1 d . . .
H9H −0.1256 0.2759 0.6338 0.071 Uiso 1 1Calc R . .
C10C −0.1091(4) 0.32283(14) 0.43189(16) 0.0480(7) Uani 1 1 d . . .
C11C −0.1205(5) 0.26100(15) 0.40956(18) 0.0576(8) Uani 1 1 d . . .
H11H −0.0053 0.2347 0.4300 0.069 Uiso 1 1Calc R . .
C12C −0.2993(5) 0.23698(15) 0.35737(19) 0.0654(9) Uani 1 1 d . . .
H12H −0.3028 0.1951 0.3425 0.078 Uiso 1 1Calc R . .
C13C −0.4728(5) 0.27577(17) 0.32758(19) 0.0656(9) Uani 1 1 d . . .
H13H −0.5951 0.2598 0.2937 0.079 Uiso 1 1Calc R . .
C14C −0.4638(5) 0.33782(16) 0.34811(18) 0.0615(9) Uani 1 1 d . . .
H14H −0.5788 0.3641 0.3273 0.074 Uiso 1 1Calc R . .
C15C −0.2835(4) 0.36109(14) 0.39973(16) 0.0531(8) Uani 1 1 d . . .
H15H −0.2785 0.4032 0.4133 0.064 Uiso 1 1Calc R . .
C16C 0.9378(5) 0.45811(14) 0.67206(17) 0.0562(8) Uani 1 1 d . . .
H16H 0.9400 0.4954 0.6409 0.067 Uiso 1 1Calc R . .
C17C 1.1149(4) 0.44529(14) 0.74066(17) 0.0495(8) Uani 1 1 d . . .
C18C 1.1251(5) 0.39092(14) 0.79030(18) 0.0596(9) Uani 1 1 d . . .
H18H 1.0185 0.3601 0.7785 0.072 Uiso 1 1Calc R . .
C19C 1.2915(5) 0.38205(15) 0.85695(19) 0.0648(9) Uani 1 1 d . . .
H19H 1.2951 0.3457 0.8903 0.078 Uiso 1 1Calc R . .
C20C 1.4530(5) 0.42719(15) 0.87436(19) 0.0588(9) Uani 1 1 d . . .
C21C 1.4488(5) 0.48071(15) 0.82602(19) 0.0631(9) Uani 1 1 d . . .
H21H 1.5579 0.5109 0.8371 0.076 Uiso 1 1Calc R . .
C22C 1.2784(5) 0.48934(14) 0.75977(18) 0.0605(9) Uani 1 1 d . . .
H22H 1.2745 0.5261 0.7272 0.073 Uiso 1 1Calc R . .

C23C 1.7857(5) 0.45639(17) 0.9618(2) 0.0864(12) Uani 1 1 d . . .
H23AH 1.8724 0.4608 0.9103 0.130 Uiso 1 1Calc R . .
H23BH 1.8803 0.4417 1.0114 0.130 Uiso 1 1Calc R . .
H23CH 1.7234 0.4958 0.9766 0.130 Uiso 1 1Calc R . .
H1H 0.646(5) 0.4783(8) 0.5642(18) 0.081(12) Uiso 1 1 d D . .

loop_
_atom_site_aniso_label
_atom_site_aniso_U_11
_atom_site_aniso_U_22
_atom_site_aniso_U_33
_atom_site_aniso_U_23
_atom_site_aniso_U_13
_atom_site_aniso_U_12
O1 0.0716(14) 0.0875(18) 0.0805(14) 0.0059(13) −0.0267(13) −0.0003(14)
N1 0.0500(14) 0.0498(17) 0.0531(14) 0.0022(13) −0.0004(12) −0.0064(12)
N2 0.0508(14) 0.0490(16) 0.0647(15) 0.0015(13) −0.0052(13) −0.0014(13)
N3 0.0482(14) 0.0552(17) 0.0598(15) 0.0016(14) −0.0041(12) 0.0009(13)
N4 0.0579(16) 0.0533(18) 0.0613(15) 0.0042(16) −0.0160(13) −0.0099(14)
N5 0.0564(15) 0.0552(17) 0.0527(13) −0.0026(13) −0.0087(13) 0.0014(13)
C1 0.0485(17) 0.051(2) 0.0490(16) −0.0040(16) 0.0028(14) −0.0025(16)
C2 0.0428(15) 0.0500(19) 0.0419(14) −0.0030(14) 0.0042(13) −0.0012(15)
C3 0.0433(16) 0.0427(19) 0.0501(16) −0.0005(15) 0.0057(13) 0.0034(14)
C4 0.0447(17) 0.0488(19) 0.0410(15) 0.0043(14) −0.0051(14) −0.0043(15)
C5 0.0476(17) 0.059(2) 0.0583(18) 0.0047(17) −0.0004(14) −0.0020(17)
C6 0.068(2) 0.052(2) 0.071(2) 0.0052(18) −0.0056(18) 0.0042(18)
C7 0.081(2) 0.057(2) 0.064(2) 0.0151(18) −0.0050(19) −0.013(2)
C8 0.067(2) 0.075(3) 0.0627(19) 0.013(2) 0.0099(17) −0.017(2)
C9 0.0534(19) 0.061(2) 0.0617(18) 0.0046(17) 0.0052(16) −0.0035(17)
C10 0.0469(16) 0.052(2) 0.0443(15) 0.0003(15) 0.0005(13) 0.0002(15)
C11 0.0501(18) 0.063(2) 0.0590(18) 0.0030(17) −0.0062(15) −0.0010(16)
C12 0.061(2) 0.061(2) 0.073(2) −0.0055(18) −0.0017(18) −0.0099(18)
C13 0.0538(19) 0.086(3) 0.0563(18) 0.003(2) −0.0070(16) −0.0115(19)
C14 0.0521(19) 0.080(3) 0.0519(18) 0.0062(18) −0.0032(15) 0.0037(18)
C15 0.0516(17) 0.061(2) 0.0460(15) 0.0004(16) 0.0014(14) 0.0075(16)
C16 0.0565(18) 0.055(2) 0.0555(17) −0.0023(16) −0.0072(15) −0.0053(16)
C17 0.0536(17) 0.048(2) 0.0467(15) −0.0022(15) −0.0003(14) −0.0017(15)
C18 0.0570(19) 0.056(2) 0.0641(18) −0.0030(17) −0.0070(16) −0.0051(16)
C19 0.068(2) 0.057(2) 0.068(2) 0.0084(17) −0.0069(17) −0.0002(18)
C20 0.0568(19) 0.062(2) 0.0558(17) −0.0054(17) −0.0109(16) 0.0062(17)
C21 0.062(2) 0.060(2) 0.0659(19) −0.0093(18) −0.0106(17) −0.0094(17)
C22 0.069(2) 0.048(2) 0.0624(19) 0.0035(16) −0.0078(17) −0.0076(17)

C23 0.063(2) 0.107(3) 0.086(2) −0.021(2) −0.0188(19) −0.002(2)

_geom_special_details
;
All esds (except the esd in the dihedral angle between two l.s. planes)
are estimated using the fullCovariance matrix. TheCell esds are taken
into account individually in the estimation of esds in distances, angles
and torsion angles;Correlations between esds inCell parameters are only
used when they are defined byCrystal symmetry. An approximate (isotropic)
treatment ofCell esds is used for estimating esds involving l.s. planes.
;

loop_
_geom_bond_atom_site_label_1
_geom_bond_atom_site_label_2
_geom_bond_distance
_geom_bond_site_symmetry_2
_geom_bond_publ_flag
O1C20 1.377(3) . ?
O1C23 1.426(3) . ?
N1C2 1.330(3) . ?
N1C1 1.332(3) . ?
N2N3 1.343(3) . ?
N2C1 1.353(3) . ?
N3C3 1.334(3) . ?
N4C1 1.371(3) . ?
N4N5 1.385(3) . ?
N4H1 0.894(10) . ?
N5C16 1.283(3) . ?
C2C3 1.423(3) . ?
C2C4 1.477(4) . ?
C3C10 1.490(3) . ?
C4C5 1.385(4) . ?
C4C9 1.390(4) . ?
C5C6 1.371(4) . ?
C5H5 0.9300 . ?
C6C7 1.376(4) . ?
C6H6 0.9300 . ?
C7C8 1.378(4) . ?
C7H7 0.9300 . ?
C8C9 1.375(4) . ?
C8H8 0.9300 . ?

C9H9 0.9300 . ?
C10C11 1.376(4) . ?
C10C15 1.392(3) . ?
C11C12 1.386(3) . ?
C11H11 0.9300 . ?
C12C13 1.385(4) . ?
C12H12 0.9300 . ?
C13C14 1.374(4) . ?
C13H13 0.9300 . ?
C14C15 1.382(3) . ?
C14H14 0.9300 . ?
C15H15 0.9300 . ?
C16C17 1.457(3) . ?
C16H16 0.9300 . ?
C17C22 1.380(4) . ?
C17C18 1.388(4) . ?
C18C19 1.379(3) . ?
C18H18 0.9300 . ?
C19C20 1.385(4) . ?
C19H19 0.9300 . ?
C20C21 1.362(4) . ?
C21C22 1.391(3) . ?
C21H21 0.9300 . ?
C22H22 0.9300 . ?
C23H23A 0.9600 . ?
C23H23B 0.9600 . ?
C23H23C 0.9600 . ?

loop_
_geom_angle_atom_site_label_1
_geom_angle_atom_site_label_2
_geom_angle_atom_site_label_3
_geom_angle
_geom_angle_site_symmetry_1
_geom_angle_site_symmetry_3
_geom_angle_publ_flag
C20 O1C23 117.7(3) . . ?
C2N1C1 115.6(2) . . ?
N3N2C1 117.3(2) . . ?
C3N3N2 120.2(2) . . ?
C1N4N5 122.2(3) . . ?
C1N4H1 118.9(18) . . ?

N5N4H1 118.9(18) . . ?
C16N5N4 112.7(3) . . ?
N1C1N2 126.3(2) . . ?
N1C1N4 122.0(2) . . ?
N2C1N4 111.7(3) . . ?
N1C2C3 120.9(3) . . ?
N1C2C4 115.5(2) . . ?
C3C2C4 123.6(2) . . ?
N3C3C2 119.4(2) . . ?
N3C3C10 113.9(2) . . ?
C2C3C10 126.7(3) . . ?
C5C4C9 118.9(3) . . ?
C5C4C2 119.7(3) . . ?
C9C4C2 121.4(3) . . ?
C6C5C4 120.6(3) . . ?
C6C5H5 119.7 . . ?
C4C5H5 119.7 . . ?
C5C6C7 120.4(3) . . ?
C5C6H6 119.8 . . ?
C7C6H6 119.8 . . ?
C6C7C8 119.5(3) . . ?
C6C7H7 120.3 . . ?
C8C7H7 120.3 . . ?
C9C8C7 120.5(3) . . ?
C9C8H8 119.7 . . ?
C7C8H8 119.7 . . ?
C8C9C4 120.1(3) . . ?
C8C9H9 120.0 . . ?
C4C9H9 120.0 . . ?
C11C10C15 117.7(2) . . ?
C11C10C3 123.5(2) . . ?
C15C10C3 118.7(3) . . ?
C10C11C12 121.7(3) . . ?
C10C11H11 119.2 . . ?
C12C11H11 119.2 . . ?
C13C12C11 119.5(3) . . ?
C13C12H12 120.2 . . ?
C11C12H12 120.2 . . ?
C14C13C12 119.9(3) . . ?
C14C13H13 120.1 . . ?
C12C13H13 120.1 . . ?
C13C14C15 119.8(3) . . ?

C13C14H14 120.1 . . ?
C15C14H14 120.1 . . ?
C14C15C10 121.4(3) . . ?
C14C15H15 119.3 . . ?
C10C15H15 119.3 . . ?
N5C16C17 121.7(3) . . ?
N5C16H16 119.1 . . ?
C17C16H16 119.1 . . ?
C22C17C18 117.6(2) . . ?
C22C17C16 119.4(3) . . ?
C18C17C16 123.0(3) . . ?
C19C18C17 120.8(3) . . ?
C19C18H18 119.6 . . ?
C17C18H18 119.6 . . ?
C18C19C20 120.2(3) . . ?
C18C19H19 119.9 . . ?
C20C19H19 119.9 . . ?
C21C20 O1 125.0(3) . . ?
C21C20C19 120.4(3) . . ?
O1C20C19 114.7(3) . . ?
C20C21C22 118.8(3) . . ?
C20C21H21 120.6 . . ?
C22C21H21 120.6 . . ?
C17C22C21 122.3(3) . . ?
C17C22H22 118.8 . . ?
C21C22H22 118.8 . . ?
O1C23H23A 109.5 . . ?
O1C23H23B 109.5 . . ?
H23AC23H23B 109.5 . . ?
O1C23H23C 109.5 . . ?
H23AC23H23C 109.5 . . ?
H23BC23H23C 109.5 . . ?

loop_
_geom_torsion_atom_site_label_1
_geom_torsion_atom_site_label_2
_geom_torsion_atom_site_label_3
_geom_torsion_atom_site_label_4
_geom_torsion
_geom_torsion_site_symmetry_1
_geom_torsion_site_symmetry_2
_geom_torsion_site_symmetry_3

_geom_torsion_site_symmetry_4
_geom_torsion_publ_flag
C1N2N3C3 3.3(4) . . . . ?
C1N4N5C16 −179.3(3) . . . . ?
C2N1C1N2 2.2(4) . . . . ?
C2N1C1N4 −177.5(3) . . . . ?
N3N2C1N1 −5.5(4) . . . . ?
N3N2C1N4 174.2(2) . . . . ?
N5N4C1N1 −3.7(4) . . . . ?
N5N4C1N2 176.5(2) . . . . ?
C1N1C2C3 3.0(4) . . . . ?
C1N1C2C4 −177.6(3) . . . . ?
N2N3C3C2 1.5(4) . . . . ?
N2N3C3C10 −177.0(2) . . . . ?
N1C2C3N3 −4.9(4) . . . . ?
C4C2C3N3 175.8(3) . . . . ?
N1C2C3C10 173.3(3) . . . . ?
C4C2C3C10 −6.0(4) . . . . ?
N1C2C4C5 −58.7(3) . . . . ?
C3C2C4C5 120.6(3) . . . . ?
N1C2C4C9 119.4(3) . . . . ?
C3C2C4C9 −61.2(4) . . . . ?
C9C4C5C6 1.7(4) . . . . ?
C2C4C5C6 179.9(2) . . . . ?
C4C5C6C7 −1.9(4) . . . . ?
C5C6C7C8 0.2(4) . . . . ?
C6C7C8C9 1.6(4) . . . . ?
C7C8C9C4 −1.8(4) . . . . ?
C5C4C9C8 0.2(4) . . . . ?
C2C4C9C8 −178.0(2) . . . . ?
N3C3C10C11 146.2(3) . . . . ?
C2C3C10C11 −32.2(4) . . . . ?
N3C3C10C15 −30.7(4) . . . . ?
C2C3C10C15 150.9(3) . . . . ?
C15C10C11C12 −0.4(4) . . . . ?
C3C10C11C12 −177.3(3) . . . . ?
C10C11C12C13 −0.8(5) . . . . ?
C11C12C13C14 1.5(5) . . . . ?
C12C13C14C15 −1.1(5) . . . . ?
C13C14C15C10 −0.1(4) . . . . ?
C11C10C15C14 0.8(4) . . . . ?
C3C10C15C14 177.9(2) . . . . ?

N4N5C16C17 177.7(2) . . . . ?
N5C16C17C22 −178.1(3) . . . . ?
N5C16C17C18 0.2(4) . . . . ?
C22C17C18C19 0.9(5) . . . . ?
C16C17C18C19 −177.4(3) . . . . ?
C17C18C19C20 −0.9(5) . . . . ?
C23 O1C20C21 −3.2(4) . . . . ?
C23 O1C20C19 177.4(3) . . . . ?
C18C19C20C21 0.1(5) . . . . ?
C18C19C20 O1 179.5(3) . . . . ?
O1C20C21C22 −178.7(3) . . . . ?
C19C20C21C22 0.7(5) . . . . ?
C18C17C22C21 0.0(4) . . . . ?
C16C17C22C21 178.3(3) . . . . ?
C20C21C22C17 −0.7(5) . . . . ?

_diffrn_measured_fraction_theta_max 0.871
_diffrn_reflns_theta_full 26.00
_diffrn_measured_fraction_theta_full 0.999
_refine_diff_density_max 0.155
_refine_diff_density_min −0.187
_refine_diff_density_rms 0.038

Molecule 3Cif

data_c:\nadeem

_audit_creation_method SHELXL−97
_chemical_name_systematic
;
{4−[(5,6−Diphenyl−[1,2,4]triazin−3−yl)−hydrazonomethyl]−phenyl}−dimethyl−amine
;
_chemical_name_common
;
{4−[(5,6−Diphenyl−[1,2,4]triazin−3−yl)−hydrazonomethyl]−phenyl}−dimethyl−amine
;
_chemical_melting_point ?
_chemical_formula_moiety 'C24H22N6'
_chemical_formula_sum
 'C24H22N6'
_chemical_formula_weight 394.48

loop_

```
 _atom_type_symbol
 _atom_type_description
 _atom_type_scat_dispersion_real
 _atom_type_scat_dispersion_imag
 _atom_type_scat_source
 'C'  'C'   0.0181   0.0091
 'International Tables VolC Tables 4.2.6.8 and 6.1.1.4'
 'H'  'H'   0.0000   0.0000
 'International Tables VolC Tables 4.2.6.8 and 6.1.1.4'
 'N'  'N'   0.0311   0.0180
 'International Tables VolC Tables 4.2.6.8 and 6.1.1.4'

_symmetry_cell_setting            'Triclinic'
_symmetry_space_group_name_H−M    'P −1'
_symmetry_space_group_name_Hall   '−P 1'

loop_
 _symmetry_equiv_pos_as_xyz
 'x, y, z'
 '−x, −y, −z'

_cell_length_a                    8.8933(10)
_cell_length_b                    11.1477(11)
_cell_length_c                    11.7484(13)
_cell_angle_alpha                 91.212(9)
_cell_angle_beta                  106.044(10)
_cell_angle_gamma                 108.519(9)
_cell_volume                      1053.8(2)
_cell_formula_units_Z             2
_cell_measurement_temperature     296(2)
_cell_measurement_reflns_used     2729
_cell_measurement_theta_min       2.8089
_cell_measurement_theta_max       29.3928

_exptl_crystal_description        'prismatic'
_exptl_crystal_colour             'red'
_exptl_crystal_size_max           0.32
_exptl_crystal_size_mid           0.26
_exptl_crystal_size_min           0.17
_exptl_crystal_density_meas       ?
_exptl_crystal_density_diffrn     1.243
_exptl_crystal_density_method     'not measured'
```

_exptl_crystal_F_000 416
_exptl_absorpt_coefficient_mu 0.077
_exptl_absorpt_correction_T_min 0.88996
_exptl_absorpt_correction_T_max 1.00000
_exptl_absorpt_correction_type 'multi−scan'
_exptl_absorpt_process_details
;
CrysAlisPro, Agilent Technologies,
Version 1.171.36.20 (release 27−06−2012CrysAlis171 .NET)
(compiled Jul 11 2012,15:38:31)
Empirical absorptionCorrection using sphericalHarmonics,
implemented in SCALE3 ABSPACK scaling algorithm.
;

_exptl_special_details ?

_diffrn_ambient_temperature 296(2)
_diffrn_radiation_wavelength 0.71073
_diffrn_radiation_type MoK\a
_diffrn_radiation_source 'fine−focus sealed tube'
_diffrn_radiation_monochromator graphite
_diffrn_measurement_device_type 'SuperNova, Dual,Cu at zero, Atlas,CCD'
_diffrn_measurement_method '\w scans'
_diffrn_detector_area_resol_mean ?
_diffrn_standards_number 0
_diffrn_standards_interval_count .
_diffrn_standards_interval_time .
_diffrn_standards_decay_% ?
_diffrn_reflns_number 9379
_diffrn_reflns_av_R_equivalents 0.0252
_diffrn_reflns_av_sigmaI/netI 0.0376
_diffrn_reflns_limit_h_min −11
_diffrn_reflns_limit_h_max 9
_diffrn_reflns_limit_k_min −12
_diffrn_reflns_limit_k_max 14
_diffrn_reflns_limit_l_min −16
_diffrn_reflns_limit_l_max 15
_diffrn_reflns_theta_min 2.81
_diffrn_reflns_theta_max 29.45
_reflns_number_total 5004
_reflns_number_gt 3405
_reflns_threshold_expression >2sigma(I)

_computing_data_collection 'CrysAlis PRO (Agilent, 2012)'
_computing_cell_refinement 'CrysAlis PRO (Agilent, 2012)'
_computing_data_reduction 'CrysAlis PRO (Agilent, 2012)'
_computing_structure_solution 'SHELXS−97 (Sheldrick, 1990)'
_computing_structure_refinement 'SHELXL−97 (Sheldrick, 1997)'
_computing_molecular_graphics ?
_computing_publication_material ?

_iucr_refine_instructions_details
;
TITL 14098 in P−1
CELL 0.71073 8.8933 11.1477 11.7484 91.212 106.044 108.519
ZERR 2.0000 0.0010 0.0011 0.0013 0.009 0.010 0.009
LATT 1
SFACCHN
UNIT 48 44 12
MERG 2
FMAP 2
GRID
PLAN 20
TEMP 23
SIZE 0.17 0.26 0.32
BOND $H
HTAB
CONF
L.S. 20
ACTA
EXTI 0.01392
WGHT 0.04620 0.35700 0.00000 0.00000 0.00000 0.33330
FVAR 6.11470
N1 3 0.23539 0.20313 0.27176 11.00000 0.03926 0.04955 =
 0.03608 −0.00672 0.00955 0.01206
N2 3 −0.00809 0.04621 0.14092 11.00000 0.04349 0.05014 =
 0.03287 −0.00682 0.01177 0.01020
N3 3 −0.09474 0.05073 0.21620 11.00000 0.04139 0.04797 =
 0.03569 −0.00442 0.01054 0.01030
N5 3 0.39670 0.17391 0.10685 11.00000 0.04074 0.04991 =
 0.03995 −0.00179 0.01314 0.01242
N4 3 0.23205 0.10501 0.09231 11.00000 0.04079 0.05465 =
 0.03838 −0.00997 0.01361 0.00774
C16 1 0.44741 0.15251 0.01888 11.00000 0.04600 0.04829 =

```
     0.03846 −0.00230  0.01396  0.01278
AFIX 43
H16   2  0.37164  0.09580 −0.04639 11.00000 −1.20000
AFIX 0
C2    1  0.14505  0.21107  0.34258 11.00000  0.03809  0.04667 =
     0.03442 −0.00394  0.00779  0.01453
C3    1 −0.02206  0.13014  0.31745 11.00000  0.03967  0.04331 =
     0.03105 −0.00018  0.00855  0.01431
C17   1  0.61704  0.21217  0.01568 11.00000  0.04601  0.04384 =
     0.03697  0.00176  0.01341  0.01382
C10   1 −0.12587  0.12870  0.39699 11.00000  0.04057  0.04051 =
     0.03657 −0.00144  0.01135  0.01074
C18   1  0.66255  0.17611 −0.08029 11.00000  0.04693  0.05321 =
     0.04183 −0.00938  0.01271  0.00835
AFIX 43
H18   2  0.58232  0.11706 −0.14211 11.00000 −1.20000
AFIX 0
C1    1  0.15386  0.11962  0.17231 11.00000  0.04059  0.04192 =
     0.03392 −0.00085  0.01035  0.01365
C22   1  0.74240  0.30237  0.10573 11.00000  0.05630  0.04677 =
     0.03490  0.00149  0.01558  0.01370
AFIX 43
H22   2  0.71625  0.32991  0.17072 11.00000 −1.20000
AFIX 0
N6    3  1.10876  0.36140 −0.00191 11.00000  0.04474  0.06378 =
     0.06240 −0.00211  0.01653  0.00591
C20   1  0.94894  0.31381  0.00399 11.00000  0.04504  0.04397 =
     0.04680  0.00482  0.01239  0.01191
C15   1 −0.28761  0.12828  0.34960 11.00000  0.04376  0.05221 =
     0.04616  0.00501  0.01313  0.01377
AFIX 43
H15   2 −0.33189  0.12517  0.26759 11.00000 −1.20000
AFIX 0
C21   1  0.90269  0.35127  0.10086 11.00000  0.05147  0.04538 =
     0.03587 −0.00006  0.00625  0.00803
AFIX 43
H21   2  0.98247  0.41040  0.16282 11.00000 −1.20000
AFIX 0
C12   1 −0.16206  0.12897  0.59179 11.00000  0.07265  0.07116 =
     0.04001  0.00346  0.02346  0.02361
AFIX 43
H12   2 −0.12095  0.12682  0.67333 11.00000 −1.20000
```

```
AFIX 0
C19   1  0.82241  0.22493 −0.08681 11.00000  0.05053  0.05627 =
     0.04823 −0.00880  0.02124  0.00968
AFIX 43
H19   2  0.84716  0.19842 −0.15291 11.00000 −1.20000
AFIX 0
C11   1 −0.06461  0.12784  0.51938 11.00000  0.05270  0.06139 =
     0.03865  0.00041  0.01337  0.02169
AFIX 43
H11   2  0.04264  0.12651  0.55231 11.00000 −1.20000
AFIX 0
C4    1  0.22613  0.31234  0.44614 11.00000  0.03678  0.06339 =
     0.04295 −0.01709  0.01104  0.00797
C14   1 −0.38289  0.13246  0.42355 11.00000  0.04919  0.07764 =
     0.07051  0.01040  0.02504  0.02592
AFIX 43
H14   2 −0.48985  0.13476  0.39158 11.00000 −1.20000
AFIX 0
C7    1  0.38028  0.50445  0.63536 11.00000  0.06037  0.12292 =
     0.09155 −0.07040  0.01549  0.00185
AFIX 43
H7    2  0.43290  0.56910  0.69886 11.00000 −1.20000
AFIX 0
C9    1  0.16233  0.40864  0.45611 11.00000  0.05965  0.05914 =
     0.06400 −0.01718  0.01526  0.01395
AFIX 43
H9    2  0.06610  0.40881  0.39914 11.00000 −1.20000
AFIX 0
C23   1  1.14903  0.32988 −0.10714 11.00000  0.05250  0.08042 =
     0.08300  0.00093  0.03139  0.01305
AFIX 137
H23A  2  1.12849  0.23988 −0.11804 11.00000 −1.50000
H23B  2  1.26410  0.37522 −0.09776 11.00000 −1.50000
H23C  2  1.08127  0.35360 −0.17555 11.00000 −1.50000
AFIX 0
C13   1 −0.31943  0.13322  0.54484 11.00000  0.07096  0.08269 =
     0.06612  0.00793  0.04227  0.02843
AFIX 43
H13   2 −0.38326  0.13662  0.59478 11.00000 −1.20000
AFIX 0
C24   1  1.24198  0.44671  0.09504 11.00000  0.04708  0.06166 =
     0.06690  0.00930  0.00395  0.00483
```

```
AFIX 137
H24A   2   1.21987   0.52442   0.10560  11.00000  -1.50000
H24B   2   1.34510   0.46525   0.07664  11.00000  -1.50000
H24C   2   1.24925   0.40699   0.16715  11.00000  -1.50000
AFIX 0
C8     1   0.24118   0.50521   0.55081  11.00000   0.07780   0.06850 =
       0.09326  -0.03264   0.03254   0.00996
AFIX 43
H8     2   0.19867   0.57058   0.55637  11.00000  -1.20000
AFIX 0
C6     1   0.44222   0.40916   0.62694  11.00000   0.06213   0.17406 =
       0.08734  -0.07994  -0.01848   0.04084
AFIX 43
H6     2   0.53654   0.40824   0.68561  11.00000  -1.20000
AFIX 0
C5     1   0.36685   0.31337   0.53218  11.00000   0.05765   0.13011 =
       0.07237  -0.05112  -0.01061   0.04415
AFIX 43
H5     2   0.41176   0.24941   0.52679  11.00000  -1.20000
H1     2   0.15930   0.04591   0.02181  11.00000   0.06325
HKLF 4
;
_refine_special_details
;
 Refinement of F^2^ against ALL reflections.  The weighted R-factor wR and
 goodness of fit S are based on F^2^,Conventional R-factors R are based
 on F, with F set to zero forNegative F^2^. The threshold expression of
 F^2^ > 2sigma(F^2^) is used only forCalculating R-factors(gt) etc. and is
Not relevant to theChoice of reflections for refinement.  R-factors based
 on F^2^ are statistically about twice as large as those based on F, and R-
 factors based on ALL data will be even larger.
;

_refine_ls_structure_factor_coef  Fsqd
_refine_ls_matrix_type            full
_refine_ls_weighting_scheme       Calc
_refine_ls_weighting_details
 'calc w=1/[\s^2^(Fo^2^)+(0.0462P)^2^+0.3570P] where P=(Fo^2^+2Fc^2^)/3'
_atom_sites_solution_primary      direct
_atom_sites_solution_secondary    difmap
_atom_sites_solution_hydrogens    geom
_refine_ls_hydrogen_treatment     Constr
```

_refine_ls_extinction_method SHELXL
_refine_ls_extinction_coef 0.014(3)
_refine_ls_extinction_expression
'Fc^*^=kFc[1+0.001xFc^2^\l^3^/sin(2\q)]^−1/4^'
_refine_ls_number_reflns 5004
_refine_ls_number_parameters 275
_refine_ls_number_restraints 0
_refine_ls_R_factor_all 0.0836
_refine_ls_R_factor_gt 0.0538
_refine_ls_wR_factor_ref 0.1422
_refine_ls_wR_factor_gt 0.1218
_refine_ls_goodness_of_fit_ref 1.035
_refine_ls_restrained_S_all 1.035
_refine_ls_shift/su_max 0.000
_refine_ls_shift/su_mean 0.000

loop_
_atom_site_label
_atom_site_type_symbol
_atom_site_fract_x
_atom_site_fract_y
_atom_site_fract_z
_atom_site_U_iso_or_equiv
_atom_site_adp_type
_atom_site_occupancy
_atom_site_symetry_multiplicity
_atom_site_calc_flag
_atom_site_refinement_flags
_atom_site_disorder_assembly
_atom_site_disorder_group
N1N 0.23539(17) 0.20313(14) 0.27176(12) 0.0429(4) Uani 1 1 d . . .
N2N −0.00808(18) 0.04621(14) 0.14092(12) 0.0436(4) Uani 1 1 d . . .
N3N −0.09474(17) 0.05074(14) 0.21620(12) 0.0432(4) Uani 1 1 d . . .
N5N 0.39670(18) 0.17391(14) 0.10685(13) 0.0441(4) Uani 1 1 d . . .
N4N 0.23205(18) 0.10501(15) 0.09231(13) 0.0465(4) Uani 1 1 d . . .
C16C 0.4474(2) 0.15251(17) 0.01887(16) 0.0448(4) Uani 1 1 d . . .
H16H 0.3716 0.0958 −0.0464 0.054 Uiso 1 1Calc R . .
C2C 0.1450(2) 0.21106(17) 0.34257(15) 0.0404(4) Uani 1 1 d . . .
C3C −0.0221(2) 0.13015(16) 0.31745(14) 0.0384(4) Uani 1 1 d . . .
C17C 0.6170(2) 0.21217(17) 0.01568(15) 0.0424(4) Uani 1 1 d . . .
C10C −0.1259(2) 0.12870(16) 0.39699(15) 0.0401(4) Uani 1 1 d . . .
C18C 0.6626(2) 0.17612(18) −0.08029(17) 0.0498(5) Uani 1 1 d . . .

H18H 0.5823 0.1171 −0.1421 0.060 Uiso 1 1Calc R . .
C1C 0.1539(2) 0.11962(16) 0.17231(15) 0.0391(4) Uani 1 1 d . . .
C22C 0.7424(2) 0.30237(17) 0.10573(16) 0.0465(4) Uani 1 1 d . . .
H22H 0.7163 0.3299 0.1707 0.056 Uiso 1 1Calc R . .
N6N 1.1088(2) 0.36140(17) −0.00191(16) 0.0601(5) Uani 1 1 d . . .
C20C 0.9489(2) 0.31381(17) 0.00399(17) 0.0463(4) Uani 1 1 d . . .
C15C −0.2876(2) 0.12828(18) 0.34961(17) 0.0480(4) Uani 1 1 d . . .
H15H −0.3319 0.1252 0.2676 0.058 Uiso 1 1Calc R . .
C21C 0.9027(2) 0.35127(17) 0.10085(16) 0.0478(5) Uani 1 1 d . . .
H21H 0.9825 0.4104 0.1628 0.057 Uiso 1 1Calc R . .
C12C −0.1620(3) 0.1290(2) 0.59179(18) 0.0601(6) Uani 1 1 d . . .
H12H −0.1209 0.1268 0.6733 0.072 Uiso 1 1Calc R . .
C19C 0.8224(2) 0.22494(19) −0.08681(17) 0.0526(5) Uani 1 1 d . . .
H19H 0.8472 0.1984 −0.1529 0.063 Uiso 1 1Calc R . .
C11C −0.0646(2) 0.12785(19) 0.51938(16) 0.0504(5) Uani 1 1 d . . .
H11H 0.0426 0.1265 0.5523 0.061 Uiso 1 1Calc R . .
C4C 0.2261(2) 0.31235(19) 0.44614(17) 0.0505(5) Uani 1 1 d . . .
C14C −0.3829(3) 0.1325(2) 0.4236(2) 0.0631(6) Uani 1 1 d . . .
H14H −0.4898 0.1348 0.3916 0.076 Uiso 1 1Calc R . .
C7C 0.3803(3) 0.5044(3) 0.6354(3) 0.1017(11) Uani 1 1 d . . .
H7H 0.4329 0.5691 0.6989 0.122 Uiso 1 1Calc R . .
C9C 0.1623(3) 0.4086(2) 0.4561(2) 0.0635(6) Uani 1 1 d . . .
H9H 0.0661 0.4088 0.3991 0.076 Uiso 1 1Calc R . .
C23C 1.1490(3) 0.3299(2) −0.1071(2) 0.0720(7) Uani 1 1 d . . .
H23AH 1.1285 0.2399 −0.1181 0.108 Uiso 1 1Calc R . .
H23BH 1.2641 0.3752 −0.0978 0.108 Uiso 1 1Calc R . .
H23CH 1.0813 0.3536 −0.1756 0.108 Uiso 1 1Calc R . .
C13C −0.3194(3) 0.1332(2) 0.5448(2) 0.0680(6) Uani 1 1 d . . .
H13H −0.3832 0.1366 0.5948 0.082 Uiso 1 1Calc R . .
C24C 1.2420(2) 0.4467(2) 0.0950(2) 0.0647(6) Uani 1 1 d . . .
H24AH 1.2200 0.5245 0.1055 0.097 Uiso 1 1Calc R . .
H24BH 1.3451 0.4651 0.0767 0.097 Uiso 1 1Calc R . .
H24CH 1.2492 0.4070 0.1672 0.097 Uiso 1 1Calc R . .
C8C 0.2412(3) 0.5052(2) 0.5508(3) 0.0827(8) Uani 1 1 d . . .
H8H 0.1987 0.5706 0.5564 0.099 Uiso 1 1Calc R . .
C6C 0.4422(3) 0.4091(4) 0.6269(3) 0.1178(14) Uani 1 1 d . . .
H6H 0.5365 0.4082 0.6856 0.141 Uiso 1 1Calc R . .
C5C 0.3668(3) 0.3134(3) 0.5322(2) 0.0910(10) Uani 1 1 d . . .
H5H 0.4117 0.2494 0.5268 0.109 Uiso 1 1Calc R . .
H1H 0.1593 0.0459 0.0218 0.063(6) Uiso 1 1 d R . .

loop_

_atom_site_aniso_label
_atom_site_aniso_U_11
_atom_site_aniso_U_22
_atom_site_aniso_U_33
_atom_site_aniso_U_23
_atom_site_aniso_U_13
_atom_site_aniso_U_12
N1 0.0393(8) 0.0495(9) 0.0361(8) −0.0067(6) 0.0096(6) 0.0121(6)
N2 0.0434(8) 0.0502(9) 0.0329(7) −0.0068(6) 0.0118(6) 0.0102(7)
N3 0.0414(8) 0.0480(8) 0.0357(8) −0.0044(6) 0.0105(6) 0.0103(6)
N5 0.0407(8) 0.0499(9) 0.0399(8) −0.0018(7) 0.0132(6) 0.0124(6)
N4 0.0408(8) 0.0546(9) 0.0384(8) −0.0100(7) 0.0136(7) 0.0077(7)
C16 0.0460(10) 0.0483(10) 0.0384(9) −0.0023(8) 0.0139(8) 0.0128(8)
C2 0.0381(9) 0.0467(10) 0.0344(9) −0.0039(7) 0.0078(7) 0.0145(7)
C3 0.0397(9) 0.0433(9) 0.0310(8) −0.0002(7) 0.0086(7) 0.0143(7)
C17 0.0460(10) 0.0438(10) 0.0370(9) 0.0018(7) 0.0134(8) 0.0138(8)
C10 0.0406(9) 0.0405(9) 0.0366(9) −0.0015(7) 0.0114(7) 0.0108(7)
C18 0.0469(11) 0.0532(11) 0.0418(10) −0.0094(8) 0.0127(8) 0.0084(8)
C1 0.0406(9) 0.0419(9) 0.0339(9) −0.0008(7) 0.0104(7) 0.0136(7)
C22 0.0563(11) 0.0468(10) 0.0349(9) 0.0015(8) 0.0156(8) 0.0137(8)
N6 0.0447(9) 0.0638(11) 0.0624(11) −0.0021(9) 0.0165(8) 0.0059(8)
C20 0.0451(10) 0.0440(10) 0.0468(11) 0.0048(8) 0.0124(8) 0.0119(8)
C15 0.0437(10) 0.0522(11) 0.0461(10) 0.0050(8) 0.0131(8) 0.0137(8)
C21 0.0515(11) 0.0454(10) 0.0359(9) −0.0001(8) 0.0063(8) 0.0080(8)
C12 0.0727(15) 0.0712(14) 0.0400(11) 0.0035(10) 0.0235(10) 0.0237(11)
C19 0.0506(11) 0.0563(12) 0.0482(11) −0.0088(9) 0.0212(9) 0.0097(9)
C11 0.0527(11) 0.0614(12) 0.0387(10) 0.0004(9) 0.0134(8) 0.0217(9)
C4 0.0368(9) 0.0634(12) 0.0429(10) −0.0171(9) 0.0110(8) 0.0080(8)
C14 0.0492(12) 0.0776(15) 0.0705(15) 0.0104(12) 0.0250(11) 0.0259(11)
C7 0.0604(15) 0.123(2) 0.092(2) −0.0704(18) 0.0155(15) 0.0018(15)
C9 0.0596(13) 0.0591(13) 0.0640(14) −0.0172(11) 0.0153(11) 0.0140(10)
C23 0.0525(13) 0.0804(16) 0.0830(17) 0.0009(13) 0.0314(12) 0.0130(11)
C13 0.0709(15) 0.0828(16) 0.0662(15) 0.0080(12) 0.0423(12) 0.0284(12)
C24 0.0471(11) 0.0617(13) 0.0669(14) 0.0093(11) 0.0040(10) 0.0048(9)
C8 0.0777(17) 0.0685(15) 0.093(2) −0.0327(14) 0.0325(15) 0.0100(12)
C6 0.0621(16) 0.174(3) 0.087(2) −0.080(2) −0.0185(14) 0.0409(19)
C5 0.0576(14) 0.130(2) 0.0724(16) −0.0511(16) −0.0106(12) 0.0442(15)

_geom_special_details
;
All esds (except the esd in the dihedral angle between two l.s. planes)
are estimated using the fullCovariance matrix. TheCell esds are taken

into account individually in the estimation of esds in distances, angles
and torsion angles;Correlations between esds inCell parameters are only
used when they are defined byCrystal symmetry. An approximate (isotropic)
treatment ofCell esds is used for estimating esds involving l.s. planes.
;

loop_
_geom_bond_atom_site_label_1
_geom_bond_atom_site_label_2
_geom_bond_distance
_geom_bond_site_symmetry_2
_geom_bond_publ_flag
N1C2 1.326(2) . ?
N1C1 1.347(2) . ?
N2N3 1.3335(19) . ?
N2C1 1.351(2) . ?
N3C3 1.335(2) . ?
N5C16 1.282(2) . ?
N5N4 1.380(2) . ?
N4C1 1.349(2) . ?
N4H1 0.9741 . ?
C16C17 1.454(2) . ?
C16H16 0.9300 . ?
C2C3 1.416(2) . ?
C2C4 1.488(2) . ?
C3C10 1.481(2) . ?
C17C18 1.390(2) . ?
C17C22 1.402(2) . ?
C10C15 1.390(2) . ?
C10C11 1.391(2) . ?
C18C19 1.376(3) . ?
C18H18 0.9300 . ?
C22C21 1.373(3) . ?
C22H22 0.9300 . ?
N6C20 1.372(2) . ?
N6C23 1.444(3) . ?
N6C24 1.449(3) . ?
C20C19 1.403(3) . ?
C20C21 1.409(2) . ?
C15C14 1.380(3) . ?
C15H15 0.9300 . ?
C21H21 0.9300 . ?

C12C13 1.372(3) . ?
C12C11 1.374(3) . ?
C12H12 0.9300 . ?
C19H19 0.9300 . ?
C11H11 0.9300 . ?
C4C5 1.371(3) . ?
C4C9 1.381(3) . ?
C14C13 1.379(3) . ?
C14H14 0.9300 . ?
C7C6 1.356(4) . ?
C7C8 1.358(4) . ?
C7H7 0.9300 . ?
C9C8 1.388(3) . ?
C9H9 0.9300 . ?
C23H23A 0.9600 . ?
C23H23B 0.9600 . ?
C23H23C 0.9600 . ?
C13H13 0.9300 . ?
C24H24A 0.9600 . ?
C24H24B 0.9600 . ?
C24H24C 0.9600 . ?
C8H8 0.9300 . ?
C6C5 1.383(3) . ?
C6H6 0.9300 . ?
C5H5 0.9300 . ?

loop_
_geom_angle_atom_site_label_1
_geom_angle_atom_site_label_2
_geom_angle_atom_site_label_3
_geom_angle
_geom_angle_site_symmetry_1
_geom_angle_site_symmetry_3
_geom_angle_publ_flag
C2N1C1 115.30(14) . . ?
N3N2C1 118.32(13) . . ?
N2N3C3 119.70(14) . . ?
C16N5N4 113.87(14) . . ?
C1N4N5 122.56(14) . . ?
C1N4H1 113.5 . . ?
N5N4H1 123.9 . . ?
N5C16C17 123.51(16) . . ?

N5C16H16 118.2 . . ?
C17C16H16 118.2 . . ?
N1C2C3 121.23(15) . . ?
N1C2C4 116.58(15) . . ?
C3C2C4 122.14(14) . . ?
N3C3C2 119.73(14) . . ?
N3C3C10 116.51(14) . . ?
C2C3C10 123.75(14) . . ?
C18C17C22 116.53(16) . . ?
C18C17C16 119.08(16) . . ?
C22C17C16 124.36(16) . . ?
C15C10C11 118.82(16) . . ?
C15C10C3 119.97(16) . . ?
C11C10C3 121.20(16) . . ?
C19C18C17 122.16(17) . . ?
C19C18H18 118.9 . . ?
C17C18H18 118.9 . . ?
N1C1N4 120.68(15) . . ?
N1C1N2 125.48(14) . . ?
N4C1N2 113.83(14) . . ?
C21C22C17 121.96(17) . . ?
C21C22H22 119.0 . . ?
C17C22H22 119.0 . . ?
C20N6C23 120.72(17) . . ?
C20N6C24 121.70(17) . . ?
C23N6C24 117.58(17) . . ?
N6C20C19 121.49(17) . . ?
N6C20C21 122.12(17) . . ?
C19C20C21 116.39(16) . . ?
C14C15C10 120.39(18) . . ?
C14C15H15 119.8 . . ?
C10C15H15 119.8 . . ?
C22C21C20 121.44(16) . . ?
C22C21H21 119.3 . . ?
C20C21H21 119.3 . . ?
C13C12C11 120.6(2) . . ?
C13C12H12 119.7 . . ?
C11C12H12 119.7 . . ?
C18C19C20 121.51(17) . . ?
C18C19H19 119.2 . . ?
C20C19H19 119.2 . . ?
C12C11C10 120.22(19) . . ?

C12C11H11 119.9 . . ?
C10C11H11 119.9 . . ?
C5C4C9 118.71(18) . . ?
C5C4C2 120.87(18) . . ?
C9C4C2 120.41(18) . . ?
C13C14C15 120.0(2) . . ?
C13C14H14 120.0 . . ?
C15C14H14 120.0 . . ?
C6C7C8 119.8(2) . . ?
C6C7H7 120.1 . . ?
C8C7H7 120.1 . . ?
C4C9C8 120.3(2) . . ?
C4C9H9 119.9 . . ?
C8C9H9 119.9 . . ?
N6C23H23A 109.5 . . ?
N6C23H23B 109.5 . . ?
H23AC23H23B 109.5 . . ?
N6C23H23C 109.5 . . ?
H23AC23H23C 109.5 . . ?
H23BC23H23C 109.5 . . ?
C12C13C14 119.96(19) . . ?
C12C13H13 120.0 . . ?
C14C13H13 120.0 . . ?
N6C24H24A 109.5 . . ?
N6C24H24B 109.5 . . ?
H24AC24H24B 109.5 . . ?
N6C24H24C 109.5 . . ?
H24AC24H24C 109.5 . . ?
H24BC24H24C 109.5 . . ?
C7C8C9 120.1(2) . . ?
C7C8H8 119.9 . . ?
C9C8H8 119.9 . . ?
C7C6C5 120.8(3) . . ?
C7C6H6 119.6 . . ?
C5C6H6 119.6 . . ?
C4C5C6 120.2(2) . . ?
C4C5H5 119.9 . . ?
C6C5H5 119.9 . . ?

loop_
_geom_torsion_atom_site_label_1
_geom_torsion_atom_site_label_2

_geom_torsion_atom_site_label_3
_geom_torsion_atom_site_label_4
_geom_torsion
_geom_torsion_site_symmetry_1
_geom_torsion_site_symmetry_2
_geom_torsion_site_symmetry_3
_geom_torsion_site_symmetry_4
_geom_torsion_publ_flag
C1N2N3C3 2.4(2) . . . . ?
C16N5N4C1 −176.36(17) . . . . ?
N4N5C16C17 −178.61(17) . . . . ?
C1N1C2C3 4.1(3) . . . . ?
C1N1C2C4 −173.49(17) . . . . ?
N2N3C3C2 1.6(3) . . . . ?
N2N3C3C10 −179.38(15) . . . . ?
N1C2C3N3 −5.1(3) . . . . ?
C4C2C3N3 172.36(17) . . . . ?
N1C2C3C10 175.93(16) . . . . ?
C4C2C3C10 −6.6(3) . . . . ?
N5C16C17C18 175.66(18) . . . . ?
N5C16C17C22 −2.2(3) . . . . ?
N3C3C10C15 −44.8(2) . . . . ?
C2C3C10C15 134.26(19) . . . . ?
N3C3C10C11 135.29(18) . . . . ?
C2C3C10C11 −45.7(3) . . . . ?
C22C17C18C19 0.6(3) . . . . ?
C16C17C18C19 −177.41(19) . . . . ?
C2N1C1N4 178.75(17) . . . . ?
C2N1C1N2 0.1(3) . . . . ?
N5N4C1N1 −0.4(3) . . . . ?
N5N4C1N2 178.36(16) . . . . ?
N3N2C1N1 −3.5(3) . . . . ?
N3N2C1N4 177.80(15) . . . . ?
C18C17C22C21 −1.1(3) . . . . ?
C16C17C22C21 176.84(18) . . . . ?
C23N6C20C19 5.5(3) . . . . ?
C24N6C20C19 −175.91(19) . . . . ?
C23N6C20C21 −174.41(19) . . . . ?
C24N6C20C21 4.2(3) . . . . ?
C11C10C15C14 2.7(3) . . . . ?
C3C10C15C14 −177.21(17) . . . . ?
C17C22C21C20 0.6(3) . . . . ?

N6C20C21C22 −179.71(18) . . . . ?
C19C20C21C22 0.4(3) . . . . ?
C17C18C19C20 0.3(3) . . . . ?
N6C20C19C18 179.26(19) . . . . ?
C21C20C19C18 −0.8(3) . . . . ?
C13C12C11C10 −1.2(3) . . . . ?
C15C10C11C12 −1.2(3) . . . . ?
C3C10C11C12 178.75(18) . . . . ?
N1C2C4C5 −60.9(3) . . . . ?
C3C2C4C5 121.5(2) . . . . ?
N1C2C4C9 117.9(2) . . . . ?
C3C2C4C9 −59.6(3) . . . . ?
C10C15C14C13 −1.9(3) . . . . ?
C5C4C9C8 0.9(3) . . . . ?
C2C4C9C8 −178.0(2) . . . . ?
C11C12C13C14 2.0(4) . . . . ?
C15C14C13C12 −0.4(4) . . . . ?
C6C7C8C9 0.1(5) . . . . ?
C4C9C8C7 −1.0(4) . . . . ?
C8C7C6C5 1.0(5) . . . . ?
C9C4C5C6 0.2(4) . . . . ?
C2C4C5C6 179.0(3) . . . . ?
C7C6C5C4 −1.1(5) . . . . ?

_diffrn_measured_fraction_theta_max 0.855
_diffrn_reflns_theta_full 26.00
_diffrn_measured_fraction_theta_full 0.998
_refine_diff_density_max 0.202
_refine_diff_density_min −0.160
_refine_diff_density_rms 0.035

Molecule 4Cif

data_c:\nadeem

_audit_creation_method SHELXL−97
_chemical_name_systematic
;
<i>N<i/>−(5,6−Diphenyl−[1,2,4]triazin−3−yl)−<i>N<i/>'−(2−hydroxy−benzylidene)−
hydrazine
;
_chemical_name_common

;
<i>N<i/>−(5,6−Diphenyl−[1,2,4]triazin−3−yl)−<i>N<i/>'−(2−hydroxy−benzylidene)−
hydrazine
;
_chemical_melting_point ?
_chemical_formula_moiety 'C22H17N5 O'
_chemical_formula_sum
 'C22H17N5 O'
_chemical_formula_weight 367.41

loop_
 _atom_type_symbol
 _atom_type_description
 _atom_type_scat_dispersion_real
 _atom_type_scat_dispersion_imag
 _atom_type_scat_source
 'C' 'C' 0.0181 0.0091
 'International Tables VolC Tables 4.2.6.8 and 6.1.1.4'
 'H' 'H' 0.0000 0.0000
 'International Tables VolC Tables 4.2.6.8 and 6.1.1.4'
 'N' 'N' 0.0311 0.0180
 'International Tables VolC Tables 4.2.6.8 and 6.1.1.4'
 'O' 'O' 0.0492 0.0322
 'International Tables VolC Tables 4.2.6.8 and 6.1.1.4'

_symmetry_cell_setting 'Monoclinic'
_symmetry_space_group_name_H−M 'P21/c'
_symmetry_space_group_name_Hall '−P2ybc'

loop_
 _symmetry_equiv_pos_as_xyz
 'x, y, z'
 '−x, y+1/2, −z+1/2'
 '−x, −y, −z'
 'x, −y−1/2, z−1/2'

_cell_length_a 14.9753(17)
_cell_length_b 6.1220(6)
_cell_length_c 21.038(2)
_cell_angle_alpha 90.00
_cell_angle_beta 103.992(11)
_cell_angle_gamma 90.00

```
_cell_volume                  1871.6(3)
_cell_formula_units_Z           4
_cell_measurement_temperature    296(2)
_cell_measurement_reflns_used   1567
_cell_measurement_theta_min     2.7979
_cell_measurement_theta_max     29.5040

_exptl_crystal_description       'needle'
_exptl_crystal_colour            'Yellow'
_exptl_crystal_size_max          0.43
_exptl_crystal_size_mid          0.15
_exptl_crystal_size_min          0.05
_exptl_crystal_density_meas       ?
_exptl_crystal_density_diffrn     1.304
_exptl_crystal_density_method     'not measured'
_exptl_crystal_F_000             768
_exptl_absorpt_coefficient_mu     0.084
_exptl_absorpt_correction_T_min                 0.68408
_exptl_absorpt_correction_T_max                  1.00000
_exptl_absorpt_correction_type           'multi-scan'
_exptl_absorpt_process_details
;
CrysAlisPro, Agilent Technologies,
Version 1.171.36.20 (release 27-06-2012CrysAlis171 .NET)
(compiled Jul 11 2012,15:38:31)
Empirical absorptionCorrection using sphericalHarmonics,
 implemented in SCALE3 ABSPACK scaling algorithm.
;

_exptl_special_details           ?

_diffrn_ambient_temperature       296(2)
_diffrn_radiation_wavelength      0.71073
_diffrn_radiation_type            MoK\a
_diffrn_radiation_source          'fine-focus sealed tube'
_diffrn_radiation_monochromator   graphite
_diffrn_measurement_device_type   'SuperNova, Dual,Cu at zero, Atlas,CCD'
_diffrn_measurement_method        '\w scans'
_diffrn_detector_area_resol_mean  ?
_diffrn_standards_number          0
_diffrn_standards_interval_count  .
_diffrn_standards_interval_time   .
```

```
_diffrn_standards_decay_%         ?
_diffrn_reflns_number            11939
_diffrn_reflns_av_R_equivalents   0.0620
_diffrn_reflns_av_sigmaI/netI     0.0951
_diffrn_reflns_limit_h_min        −19
_diffrn_reflns_limit_h_max        19
_diffrn_reflns_limit_k_min        −8
_diffrn_reflns_limit_k_max        7
_diffrn_reflns_limit_l_min        −29
_diffrn_reflns_limit_l_max        26
_diffrn_reflns_theta_min          2.80
_diffrn_reflns_theta_max          29.57
_reflns_number_total             4597
_reflns_number_gt                1742
_reflns_threshold_expression      >2sigma(I)

_computing_data_collection     'CrysAlis PRO (Agilent, 2012)'
_computing_cell_refinement     'CrysAlis PRO (Agilent, 2012)'
_computing_data_reduction     'CrysAlis PRO (Agilent, 2012)'
_computing_structure_solution     'SHELXS−97 (Sheldrick, 1990)'
_computing_structure_refinement   'SHELXL−97 (Sheldrick, 1997)'
_computing_molecular_graphics     ?
_computing_publication_material   ?

_iucr_refine_instructions_details
;
TITL 14097 in P2(1)/c
CELL 0.71073 5.9756 21.5731 14.9805 90.000 93.447 90.000
ZERR 4.0000 0.0018 0.0056 0.0040 0.000 0.022 0.000
LATT 1
SYMM −X, 0.5+Y, 0.5−Z
SFACCHN O
UNIT 92 76 20 4
MERG 2
FMAP 2
GRID
PLAN 20
TEMP 23
SIZE 0.05 0.08 0.48
BOND $H
HTAB
CONF
```

```
L.S. 20
ACTA 52
WGHT 0.02180 0.00000 0.00000 0.00000 0.00000 0.33330
FVAR 1.69020
O1    4   1.60874   0.41310   0.94281  11.00000   0.07147   0.08726 =
      0.08046   0.00578  −0.02662  −0.00019
N1    3   0.40402   0.35009   0.58890  11.00000   0.04992   0.04967 =
      0.05303   0.00216  −0.00043  −0.00648
N2    3   0.30440   0.43955   0.50047  11.00000   0.05052   0.04896 =
      0.06466   0.00143  −0.00525  −0.00137
N3    3   0.12818   0.40936   0.46126  11.00000   0.04805   0.05515 =
      0.05971   0.00165  −0.00407   0.00092
N4    3   0.62325   0.44122   0.58888  11.00000   0.05869   0.05261 =
      0.06208   0.00461  −0.01612  −0.00987
AFIX 43
H4    2   0.64324   0.47722   0.56601  11.00000  −1.20000
AFIX 0
N5    3   0.77881   0.41939   0.65359  11.00000   0.05624   0.05530 =
      0.05242  −0.00253  −0.00885   0.00159
C1    1   0.43856   0.40747   0.56000  11.00000   0.04820   0.05064 =
      0.04908  −0.00425   0.00275  −0.00286
C2    1   0.22268   0.32186   0.55276  11.00000   0.04271   0.04990 =
      0.04181  −0.00298   0.00426  −0.00114
C3    1   0.08431   0.35113   0.48454  11.00000   0.04319   0.04251 =
      0.04982  −0.00051   0.00565   0.00340
C4    1   0.17969   0.25920   0.58764  11.00000   0.04459   0.04857 =
      0.04099   0.00430  −0.00508  −0.00432
C5    1   0.33970   0.21325   0.58139  11.00000   0.04747   0.05908 =
      0.05816   0.00481  −0.00035  −0.00207
AFIX 43
H5    2   0.47327   0.22209   0.55521  11.00000  −1.20000
AFIX 0
C6    1   0.30303   0.15481   0.61346  11.00000   0.06740   0.05151 =
      0.07096   0.00513  −0.00564   0.00425
AFIX 43
H6    2   0.40982   0.12399   0.60725  11.00000  −1.20000
AFIX 0
C7    1   0.10916   0.14150   0.65477  11.00000   0.08041   0.05691 =
      0.06422   0.01512  −0.00507  −0.01296
AFIX 43
H7    2   0.08485   0.10188   0.67668  11.00000  −1.20000
AFIX 0
```

C8 1 −0.04866 0.18733 0.66341 11.00000 0.06669 0.07522 =
0.06261 0.01322 0.00984 −0.01721
AFIX 43
H8 2 −0.17815 0.17881 0.69264 11.00000 −1.20000
AFIX 0
C9 1 −0.01625 0.24557 0.62917 11.00000 0.05326 0.06124 =
0.06158 0.00459 0.00509 −0.00351
AFIX 43
H9 2 −0.12560 0.27588 0.63380 11.00000 −1.20000
AFIX 0
C10 1 −0.10908 0.32283 0.43189 11.00000 0.04671 0.05251 =
0.04420 0.00027 0.00045 0.00023
C11 1 −0.12049 0.26099 0.40957 11.00000 0.05002 0.06235 =
0.05891 0.00293 −0.00613 −0.00107
AFIX 43
H11 2 −0.00533 0.23465 0.43001 11.00000 −1.20000
AFIX 0
C12 1 −0.29937 0.23697 0.35738 11.00000 0.06126 0.06120 =
0.07264 −0.00545 −0.00166 −0.00994
AFIX 43
H12 2 −0.30288 0.19512 0.34249 11.00000 −1.20000
AFIX 0
C13 1 −0.47277 0.27576 0.32758 11.00000 0.05370 0.08541 =
0.05614 0.00288 −0.00703 −0.01153
AFIX 43
H13 2 −0.59508 0.25983 0.29373 11.00000 −1.20000
AFIX 0
C14 1 −0.46381 0.33783 0.34811 11.00000 0.05198 0.07977 =
0.05177 0.00622 −0.00326 0.00377
AFIX 43
H14 2 −0.57878 0.36412 0.32732 11.00000 −1.20000
AFIX 0
C15 1 −0.28347 0.36109 0.39974 11.00000 0.05158 0.06109 =
0.04589 0.00039 0.00136 0.00749
AFIX 43
H15 2 −0.27852 0.40316 0.41328 11.00000 −1.20000
AFIX 0
C16 1 0.93780 0.45812 0.67206 11.00000 0.05638 0.05521 =
0.05547 −0.00234 −0.00732 −0.00541
AFIX 43
H16 2 0.94002 0.49535 0.64084 11.00000 −1.20000
AFIX 0

```
C17   1   1.11498   0.44529   0.74065  11.00000   0.05349   0.04754 =
      0.04661  -0.00213  -0.00032  -0.00167
C18   1   1.12508   0.39093   0.79028  11.00000   0.05679   0.05639 =
      0.06396  -0.00294  -0.00697  -0.00507
AFIX 43
H18   2   1.01849   0.36013   0.77846  11.00000  -1.20000
AFIX 0
C19   1   1.29145   0.38206   0.85696  11.00000   0.06806   0.05696 =
      0.06783   0.00846  -0.00691  -0.00011
AFIX 43
H19   2   1.29513   0.34567   0.89031  11.00000  -1.20000
AFIX 0
C20   1   1.45299   0.42720   0.87435  11.00000   0.05667   0.06206 =
      0.05572  -0.00543  -0.01090   0.00618
C21   1   1.44882   0.48071   0.82603  11.00000   0.06194   0.05952 =
      0.06587  -0.00923  -0.01070  -0.00939
AFIX 43
H21   2   1.55791   0.51094   0.83713  11.00000  -1.20000
AFIX 0
C22   1   1.27842   0.48933   0.75977  11.00000   0.06931   0.04814 =
      0.06232   0.00361  -0.00772  -0.00766
AFIX 43
H22   2   1.27448   0.52606   0.72722  11.00000  -1.20000
AFIX 0
C23   1   1.78566   0.45639   0.96182  11.00000   0.06311   0.10751 =
      0.08569  -0.02149  -0.01883  -0.00220
AFIX 137
H23A   2   1.87234   0.46079   0.91027  11.00000  -1.50000
H23B   2   1.88034   0.44169   1.01138  11.00000  -1.50000
H23C   2   1.72336   0.49584   0.97659  11.00000  -1.50000
HKLF 4
;
_refine_special_details
;
 Refinement of F^2^ against ALL reflections.  The weighted R-factor wR and
 goodness of fit S are based on F^2^,Conventional R-factors R are based
 on F, with F set to zero forNegative F^2^. The threshold expression of
 F^2^ > 2sigma(F^2^) is used only forCalculating R-factors(gt) etc. and is
Not relevant to theChoice of reflections for refinement.  R-factors based
 on F^2^ are statistically about twice as large as those based on F, and R-
 factors based on ALL data will be even larger.
;
```

```
_refine_ls_structure_factor_coef  Fsqd
_refine_ls_matrix_type           full
_refine_ls_weighting_scheme      Calc
_refine_ls_weighting_details
 'calc w=1/[\s^2^(Fo^2^)+(0.0576P)^2^+0.0000P] where P=(Fo^2^+2Fc^2^)/3'
_atom_sites_solution_primary      direct
_atom_sites_solution_secondary    difmap
_atom_sites_solution_hydrogens    geom
_refine_ls_hydrogen_treatment     mixed
_refine_ls_extinction_method     None
_refine_ls_extinction_coef       ?
_refine_ls_number_reflns         4597
_refine_ls_number_parameters     257
_refine_ls_number_restraints     0
_refine_ls_R_factor_all          0.1878
_refine_ls_R_factor_gt           0.0598
_refine_ls_wR_factor_ref         0.1683
_refine_ls_wR_factor_gt          0.1240
_refine_ls_goodness_of_fit_ref   0.955
_refine_ls_restrained_S_all      0.955
_refine_ls_shift/su_max          0.000
_refine_ls_shift/su_mean         0.000

loop_
 _atom_site_label
 _atom_site_type_symbol
 _atom_site_fract_x
 _atom_site_fract_y
 _atom_site_fract_z
 _atom_site_U_iso_or_equiv
 _atom_site_adp_type
 _atom_site_occupancy
 _atom_site_symetry_multiplicity
 _atom_site_calc_flag
 _atom_site_refinement_flags
 _atom_site_disorder_assembly
 _atom_site_disorder_group
N1N 0.24100(16) 0.9759(3) 0.02249(10) 0.0594(6) Uani 1 1 d . . .
N3N 0.14906(16) 1.2823(4) 0.07838(11) 0.0631(6) Uani 1 1 d . . .
C2C 0.28801(18) 1.1305(4) 0.06162(11) 0.0519(7) Uani 1 1 d . . .
N2N 0.10195(16) 1.1322(4) 0.03740(11) 0.0656(6) Uani 1 1 d . . .
```

C3C 0.24111(18) 1.2833(4) 0.09301(12) 0.0524(7) Uani 1 1 d . . .
N5N 0.13020(17) 0.6597(4) −0.05376(11) 0.0660(6) Uani 1 1 d . . .
C1C 0.1496(2) 0.9805(5) 0.01395(13) 0.0592(7) Uani 1 1 d . . .
C4C 0.38883(19) 1.1287(4) 0.06992(11) 0.0527(7) Uani 1 1 d . . .
N4N 0.09520(19) 0.8212(4) −0.02047(12) 0.0741(7) Uani 1 1 d . . .
C15C 0.2389(2) 1.6421(4) 0.14746(13) 0.0616(7) Uani 1 1 d . . .
H15H 0.1844 1.6726 0.1166 0.074 Uiso 1 1Calc R . .
C10C 0.28417(18) 1.4457(4) 0.14384(12) 0.0505(6) Uani 1 1 d . . .
C9C 0.4370(2) 1.3147(4) 0.06129(13) 0.0619(7) Uani 1 1 d . . .
H9H 0.4059 1.4465 0.0512 0.074 Uiso 1 1Calc R . .
C16C 0.0711(2) 0.5232(4) −0.08416(13) 0.0645(8) Uani 1 1 d . . .
H16H 0.0104 0.5364 −0.0812 0.077 Uiso 1 1Calc R . .
C11C 0.36520(19) 1.4045(5) 0.19072(12) 0.0624(8) Uani 1 1 d . . .
H11H 0.3964 1.2737 0.1896 0.075 Uiso 1 1Calc R . .
C17C 0.0950(2) 0.3477(4) −0.12331(13) 0.0605(7) Uani 1 1 d . . .
C22C 0.0277(2) 0.1973(5) −0.15222(14) 0.0733(9) Uani 1 1 d . . .
H22H −0.0308 0.2092 −0.1448 0.088 Uiso 1 1Calc R . .
C7C 0.5771(2) 1.1122(6) 0.08330(14) 0.0748(9) Uani 1 1 d . . .
H7H 0.6403 1.1065 0.0874 0.090 Uiso 1 1Calc R . .
C14C 0.2736(2) 1.7923(5) 0.19612(15) 0.0711(9) Uani 1 1 d . . .
H14H 0.2425 1.9226 0.1980 0.085 Uiso 1 1Calc R . .
C12C 0.3999(2) 1.5565(6) 0.23906(13) 0.0764(9) Uani 1 1 d . . .
H12H 0.4546 1.5282 0.2699 0.092 Uiso 1 1Calc R . .
O1 O 0.25154(16) 0.4654(4) −0.10809(15) 0.1261(10) Uani 1 1 d . . .
H1H 0.2336 0.5543 −0.0848 0.151 Uiso 1 1Calc R . .
C5C 0.4365(2) 0.9344(5) 0.08544(13) 0.0644(8) Uani 1 1 d . . .
H5H 0.4049 0.8076 0.0909 0.077 Uiso 1 1Calc R . .
C8C 0.5308(2) 1.3060(6) 0.06764(14) 0.0721(9) Uani 1 1 d . . .
H8H 0.5627 1.4313 0.0613 0.086 Uiso 1 1Calc R . .
C13C 0.3538(3) 1.7491(6) 0.24151(15) 0.0804(10) Uani 1 1 d . . .
H13H 0.3772 1.8506 0.2742 0.096 Uiso 1 1Calc R . .
C18C 0.1816(2) 0.3283(6) −0.13547(17) 0.0848(10) Uani 1 1 d . . .
C6C 0.5308(2) 0.9282(5) 0.09282(14) 0.0731(8) Uani 1 1 d . . .
H6H 0.5627 0.7981 0.1043 0.088 Uiso 1 1Calc R . .
C21C 0.0456(3) 0.0312(5) −0.19158(17) 0.0931(11) Uani 1 1 d . . .
H21H 0.0001 −0.0690 −0.2102 0.112 Uiso 1 1Calc R . .
C19C 0.1992(3) 0.1626(7) −0.1761(2) 0.1140(13) Uani 1 1 d . . .
H19H 0.2568 0.1512 −0.1850 0.137 Uiso 1 1Calc R . .
C20C 0.1315(3) 0.0167(7) −0.20273(19) 0.1083(13) Uani 1 1 d . . .
H20H 0.1442 −0.0952 −0.2291 0.130 Uiso 1 1Calc R . .
H10H 0.019(3) 0.810(5) −0.0309(15) 0.107(11) Uiso 1 1 d . . .

```
loop_
 _atom_site_aniso_label
 _atom_site_aniso_U_11
 _atom_site_aniso_U_22
 _atom_site_aniso_U_33
 _atom_site_aniso_U_23
 _atom_site_aniso_U_13
 _atom_site_aniso_U_12
N1 0.0526(16) 0.0672(14) 0.0538(13) −0.0064(11) 0.0040(11) −0.0122(13)
N3 0.0500(16) 0.0739(15) 0.0629(14) −0.0089(12) 0.0088(12) −0.0101(13)
C2 0.0500(18) 0.0617(16) 0.0418(13) 0.0004(13) 0.0069(13) −0.0089(14)
N2 0.0454(15) 0.0769(16) 0.0698(15) −0.0158(14) 0.0047(12) −0.0136(13)
C3 0.0393(17) 0.0662(17) 0.0489(15) −0.0003(13) 0.0054(12) −0.0095(14)
N5 0.0576(16) 0.0661(14) 0.0672(15) −0.0087(13) 0.0016(13) −0.0039(13)
C1 0.0496(19) 0.0700(19) 0.0517(15) −0.0033(14) 0.0003(14) −0.0093(17)
C4 0.0485(18) 0.0649(17) 0.0447(14) −0.0089(13) 0.0110(12) −0.0094(15)
N4 0.0562(18) 0.0772(16) 0.0805(17) −0.0267(14) 0.0001(14) −0.0171(15)
C15 0.0598(19) 0.0673(18) 0.0581(17) −0.0003(15) 0.0147(15) −0.0107(16)
C10 0.0487(17) 0.0616(16) 0.0431(14) −0.0012(13) 0.0147(13) −0.0106(15)
C9 0.059(2) 0.0693(18) 0.0593(16) −0.0039(14) 0.0190(15) −0.0092(16)
C16 0.0554(19) 0.0680(18) 0.0627(17) −0.0115(15) 0.0000(15) −0.0068(16)
C11 0.0519(18) 0.087(2) 0.0456(15) −0.0058(15) 0.0072(13) −0.0060(16)
C17 0.0529(19) 0.0639(17) 0.0611(17) −0.0006(15) 0.0069(14) 0.0039(15)
C22 0.070(2) 0.0732(19) 0.0729(19) −0.0166(17) 0.0089(17) −0.0055(18)
C7 0.055(2) 0.106(3) 0.0648(19) −0.0170(19) 0.0160(16) −0.005(2)
C14 0.082(3) 0.0650(19) 0.071(2) −0.0086(17) 0.029(2) −0.0128(18)
C12 0.066(2) 0.105(2) 0.0530(18) −0.0089(18) 0.0038(15) −0.017(2)
O1 0.0523(16) 0.144(2) 0.179(3) −0.044(2) 0.0222(16) −0.0111(16)
C5 0.059(2) 0.0665(19) 0.0653(18) −0.0097(15) 0.0098(15) −0.0061(16)
C8 0.061(2) 0.090(2) 0.070(2) −0.0072(17) 0.0259(16) −0.0183(19)
C13 0.098(3) 0.088(2) 0.0569(19) −0.0215(18) 0.022(2) −0.030(2)
C18 0.054(2) 0.096(2) 0.097(2) −0.010(2) 0.0039(19) 0.007(2)
C6 0.060(2) 0.083(2) 0.074(2) −0.0125(17) 0.0105(16) 0.0058(18)
C21 0.098(3) 0.081(2) 0.096(3) −0.027(2) 0.015(2) 0.004(2)
C19 0.080(3) 0.130(3) 0.133(3) −0.028(3) 0.028(3) 0.027(3)
C20 0.103(4) 0.111(3) 0.107(3) −0.029(2) 0.018(3) 0.031(3)

_geom_special_details
;
 All esds (except the esd in the dihedral angle between two l.s. planes)
 are estimated using the fullCovariance matrix.  TheCell esds are taken
 into account individually in the estimation of esds in distances, angles
```

and torsion angles;Correlations between esds inCell parameters are only
used when they are defined byCrystal symmetry. An approximate (isotropic)
treatment ofCell esds is used for estimating esds involving l.s. planes.
;

loop_
_geom_bond_atom_site_label_1
_geom_bond_atom_site_label_2
_geom_bond_distance
_geom_bond_site_symmetry_2
_geom_bond_publ_flag
N1C1 1.336(3) . ?
N1C2 1.337(3) . ?
N3N2 1.338(3) . ?
N3C3 1.338(3) . ?
C2C3 1.424(3) . ?
C2C4 1.477(4) . ?
N2C1 1.337(3) . ?
C3C10 1.488(3) . ?
N5C16 1.271(3) . ?
N5N4 1.386(3) . ?
C1N4 1.362(3) . ?
C4C9 1.383(3) . ?
C4C5 1.385(4) . ?
N4H10 1.11(4) . ?
C15C14 1.381(4) . ?
C15C10 1.392(3) . ?
C15H15 0.9300 . ?
C10C11 1.389(4) . ?
C9C8 1.380(4) . ?
C9H9 0.9300 . ?
C16C17 1.450(4) . ?
C16H16 0.9300 . ?
C11C12 1.384(4) . ?
C11H11 0.9300 . ?
C17C18 1.386(4) . ?
C17C22 1.392(4) . ?
C22C21 1.378(4) . ?
C22H22 0.9300 . ?
C7C6 1.362(4) . ?
C7C8 1.373(4) . ?
C7H7 0.9300 . ?

C14C13 1.367(4) . ?
C14H14 0.9300 . ?
C12C13 1.374(4) . ?
C12H12 0.9300 . ?
O1C18 1.357(4) . ?
O1H1 0.8200 . ?
C5C6 1.384(4) . ?
C5H5 0.9300 . ?
C8H8 0.9300 . ?
C13H13 0.9300 . ?
C18C19 1.392(5) . ?
C6H6 0.9300 . ?
C21C20 1.365(5) . ?
C21H21 0.9300 . ?
C19C20 1.366(5) . ?
C19H19 0.9300 . ?
C20H20 0.9300 . ?

loop_
 _geom_angle_atom_site_label_1
 _geom_angle_atom_site_label_2
 _geom_angle_atom_site_label_3
 _geom_angle
 _geom_angle_site_symmetry_1
 _geom_angle_site_symmetry_3
 _geom_angle_publ_flag
C1N1C2 115.9(2) . . ?
N2N3C3 120.3(2) . . ?
N1C2C3 120.3(2) . . ?
N1C2C4 116.0(2) . . ?
C3C2C4 123.7(2) . . ?
C1N2N3 118.0(2) . . ?
N3C3C2 119.1(2) . . ?
N3C3C10 114.3(2) . . ?
C2C3C10 126.5(2) . . ?
C16N5N4 114.9(2) . . ?
N1C1N2 125.9(3) . . ?
N1C1N4 121.2(3) . . ?
N2C1N4 112.9(3) . . ?
C9C4C5 118.9(3) . . ?
C9C4C2 121.9(3) . . ?
C5C4C2 119.2(2) . . ?

C1N4N5 121.9(3) . . ?
C1N4H10 127.2(16) . . ?
N5N4H10 110.5(16) . . ?
C14C15C10 121.0(3) . . ?
C14C15H15 119.5 . . ?
C10C15H15 119.5 . . ?
C11C10C15 118.1(2) . . ?
C11C10C3 122.8(2) . . ?
C15C10C3 119.0(2) . . ?
C8C9C4 120.4(3) . . ?
C8C9H9 119.8 . . ?
C4C9H9 119.8 . . ?
N5C16C17 122.4(3) . . ?
N5C16H16 118.8 . . ?
C17C16H16 118.8 . . ?
C12C11C10 120.5(3) . . ?
C12C11H11 119.7 . . ?
C10C11H11 119.7 . . ?
C18C17C22 118.3(3) . . ?
C18C17C16 122.6(3) . . ?
C22C17C16 119.1(3) . . ?
C21C22C17 121.7(3) . . ?
C21C22H22 119.2 . . ?
C17C22H22 119.2 . . ?
C6C7C8 120.3(3) . . ?
C6C7H7 119.8 . . ?
C8C7H7 119.8 . . ?
C13C14C15 119.9(3) . . ?
C13C14H14 120.0 . . ?
C15C14H14 120.0 . . ?
C13C12C11 120.1(3) . . ?
C13C12H12 119.9 . . ?
C11C12H12 119.9 . . ?
C18 O1H1 109.5 . . ?
C6C5C4 120.2(3) . . ?
C6C5H5 119.9 . . ?
C4C5H5 119.9 . . ?
C7C8C9 120.0(3) . . ?
C7C8H8 120.0 . . ?
C9C8H8 120.0 . . ?
C14C13C12 120.3(3) . . ?
C14C13H13 119.9 . . ?

C12C13H13 119.9 . . ?
O1C18C17 121.9(3) . . ?
O1C18C19 118.0(3) . . ?
C17C18C19 120.0(3) . . ?
C7C6C5 120.2(3) . . ?
C7C6H6 119.9 . . ?
C5C6H6 119.9 . . ?
C20C21C22 118.7(4) . . ?
C20C21H21 120.7 . . ?
C22C21H21 120.7 . . ?
C20C19C18 119.8(4) . . ?
C20C19H19 120.1 . . ?
C18C19H19 120.1 . . ?
C21C20C19 121.6(4) . . ?
C21C20H20 119.2 . . ?
C19C20H20 119.2 . . ?

loop_
 _geom_torsion_atom_site_label_1
 _geom_torsion_atom_site_label_2
 _geom_torsion_atom_site_label_3
 _geom_torsion_atom_site_label_4
 _geom_torsion
 _geom_torsion_site_symmetry_1
 _geom_torsion_site_symmetry_2
 _geom_torsion_site_symmetry_3
 _geom_torsion_site_symmetry_4
 _geom_torsion_publ_flag
C1N1C2C3 1.9(3) . . . . ?
C1N1C2C4 −178.7(2) . . . . ?
C3N3N2C1 2.5(3) . . . . ?
N2N3C3C2 3.5(3) . . . . ?
N2N3C3C10 −174.5(2) . . . . ?
N1C2C3N3 −5.8(3) . . . . ?
C4C2C3N3 174.8(2) . . . . ?
N1C2C3C10 171.9(2) . . . . ?
C4C2C3C10 −7.4(4) . . . . ?
C2N1C1N2 4.5(4) . . . . ?
C2N1C1N4 −174.6(2) . . . . ?
N3N2C1N1 −6.9(4) . . . . ?
N3N2C1N4 172.3(2) . . . . ?
N1C2C4C9 129.3(2) . . . . ?

C3C2C4C9 −51.3(3) . . . . ?
N1C2C4C5 −49.3(3) . . . . ?
C3C2C4C5 130.1(3) . . . . ?
N1C1N4N5 −6.6(4) . . . . ?
N2C1N4N5 174.1(2) . . . . ?
C16N5N4C1 −179.4(2) . . . . ?
C14C15C10C11 0.2(4) . . . . ?
C14C15C10C3 176.3(2) . . . . ?
N3C3C10C11 143.3(2) . . . . ?
C2C3C10C11 −34.6(4) . . . . ?
N3C3C10C15 −32.6(3) . . . . ?
C2C3C10C15 149.5(2) . . . . ?
C5C4C9C8 0.4(4) . . . . ?
C2C4C9C8 −178.2(2) . . . . ?
N4N5C16C17 178.0(2) . . . . ?
C15C10C11C12 −0.7(4) . . . . ?
C3C10C11C12 −176.6(2) . . . . ?
N5C16C17C18 −6.3(4) . . . . ?
N5C16C17C22 176.5(3) . . . . ?
C18C17C22C21 0.6(4) . . . . ?
C16C17C22C21 178.0(3) . . . . ?
C10C15C14C13 0.2(4) . . . . ?
C10C11C12C13 0.7(4) . . . . ?
C9C4C5C6 0.7(4) . . . . ?
C2C4C5C6 179.3(2) . . . . ?
C6C7C8C9 −0.2(4) . . . . ?
C4C9C8C7 −0.7(4) . . . . ?
C15C14C13C12 −0.2(4) . . . . ?
C11C12C13C14 −0.2(4) . . . . ?
C22C17C18 O1 −178.6(3) . . . . ?
C16C17C18 O1 4.1(5) . . . . ?
C22C17C18C19 0.5(5) . . . . ?
C16C17C18C19 −176.8(3) . . . . ?
C8C7C6C5 1.3(4) . . . . ?
C4C5C6C7 −1.6(4) . . . . ?
C17C22C21C20 −0.7(5) . . . . ?
O1C18C19C20 177.7(4) . . . . ?
C17C18C19C20 −1.4(6) . . . . ?
C22C21C20C19 −0.2(6) . . . . ?
C18C19C20C21 1.2(6) . . . . ?

_diffrn_measured_fraction_theta_max   0.874

_diffrn_reflns_theta_full 26.00
_diffrn_measured_fraction_theta_full 0.999
_refine_diff_density_max 0.129
_refine_diff_density_min −0.161
_refine_diff_density_rms 0.035
